# Supplementary material for: What influences cancer treatment service access in Ghana? A critical interpretive synthesis
Source: BMJ Open. 2022 Oct 5;12(10):e065153. doi: 10.1136/bmjopen-2022-065153 (PMC9535186; doi:10.1136/bmjopen-2022-065153)
Supplement: Supplementary data [file bmjopen-2022-065153supp001.pdf]

1

Supplementary Materials

Supplementary Table 1. Systematic search strategy

	**Search topic**		**Ovid MedLine**	**Web of Science**	**CINAHL**	**African Index Medicus**
1	Ghana*		1. exp Ghana/ 2. Ghana.ti,ab. 3. 1 or 2	1. TS=Ghana OR AB=Ghana	1. Ghana	1. Ghana
2	Health service access / patient uptake of services		4. health services accessibility/ or *health services accessibility/ or health equity/ or *healthcare disparities/ 5.*attitude to health/ or exp health knowledge, attitudes, practice/ or	2. TS=(Health service access) 3. AB=((health* NEAR/2 access*) OR (treatment) OR accessibility OR (financ* NEAR/2 (burden* OR impact* OR barrier*)) OR inequalit* OR inequit* OR (social* NEAR/1 economic) OR (social* NEAR/2 (determinant* OR	2. (treatment or therapy or intervention or intervention OR (MH "Health Services Accessibility+"))	-

			*treatment adherence and compliance/ or exp patient acceptance of health care/ or exp patient dropouts/ or exp patient satisfaction/ or exp patient preference/ or *treatment refusal/<br>6. ((financ* adj2 (burden or impact or barrier*)) or (health* adj2 access) or inequit* or inequalit* or (social* adj1 economic*) or (social* adj2 (determinant* or disparit* or barrier*)) or accessibility or	disparit* OR barrier*)) OR (catastrophic NEAR/1 cost*))		

3

			treatment* or (catastrophic adj1 cost*)).ti,ab.<br>7. 4 or 5 or 6			
3	Cancer		8. exp Neoplasms/<br>9. cancer*.ti,ab<br>10. 8 or 9	4. TS=cancer OR AB =cancer*	3. Cancer	2. Cancer
Final search			3 and 7 and 10	1 and (2 or 3) and 4 [Indexes=SCI-EXPANDED, SSCI, A&HCI, CPCI-S, CPCI-SSH, BKCI-S, BKCI-SSH, ESCI, CCR-EXPANDED, IC Timespan=All years]	1 and 2 and 3	1 and 2
Total hits (all time)			145	102	68	10

Total hits in 10 years (since 01/01/2011)		114	92	62 (59 once duplicated removed)	
Date of search		26/03/2021	26/03/2021	26/03/2021	26/03/2021
Repeated search		29/03/2022	29/03/2022	29/03/2022	29/03/2022

Evidence synthesis tables

Supplementary Table 2: Summary of included studies

Author and year	Setting (region)	Study approach	Population and sample	Methods (design, sampling, data analysis)	Phenomenon	Findings	Limitations noted by authors (if any)	Candidacy stage
Binka et al., 2019	Rural community setting and catholic hospital; North Tongu District (Volta region)	Qualitative (deductive)	Sample 1: 15 cervical cancer patients<br>Sample 2: 40 women aged between 30 and 65 registered at the hospital but not screened	Semi-structured interviews and focus group, convenience sampling, deductive thematic analysis	Cervical cancer screening and treatment uptake	- financial burden of treatment due to a lack of government subsidies for those with low household income<br>- expected provision of financial support with husbands' role<br>- Spiritual beliefs about the cause of disease and in the efficacy of traditional medicine led to spiritual and traditional alternatives being used first.<br>Possible institutional-level barriers (privacy, health worker attitudes, potential misdiagnosis) and unavailability of screening and treatment facilities in rural areas	-	Presentation, negotiation, acceptance
Aziato et al., 2015	Tertiary clinic, Accra Region	Qualitative (exploratory descriptive)	12 Ghanaian women who have undergone a mastectomy (speaking English, Twi, or Ewe)	Semi-structured interviews, purposive sampling, thematic analysis	Breast cancer treatment intentions	Treatment decisions are based on knowledge and perceptions and influenced by husbands and family; without their financial and emotion support may led to delays<br>Patients believed that alternative treatment could treat breast cancer, and sought them after diagnosis, which was influenced by husbands' views<br>Patients perceived health systems reason for treatment delays were:	-	Negotiation, acceptance

5

						- incorrect diagnosis at district level<br>- waiting for biopsy results		
Asoogo et al., 2015	Tertiary clinic, Ashanti Region	Qualitative descriptive	30 women diagnosed with breast cancer who presented with Stage II and Stage III	semi-structured interviews, convenience sampling, content data analysis	Breast cancer presentation	- financial barriers, including leading to alternative use<br>- misinformation, considering abnormalities normal development<br>- fear of treatment and outcomes influenced by supports and others in their community's experiences<br>- prioritising care of other<br>- belief in the efficacy of alternative medicines led to seeking first, influenced by affordability<br>- fear of losing female identity and capacity for motherhood	-	Presentation
Martei et al., 2018	Tertiary clinic, Accra Region	Qualitative (inductive)	31 women with breast cancer	Semi structured interviews, purposive sampling, grounded theory	Breast cancer presentation	- financial barriers including prioritisation costs of family and frustration on poor coverage by NHIS<br>- negative husband views<br>- loss of gender identity<br>- misinformation, considering abnormalities harmless, misconceptions that treatment was deadly<br>The church played a supportive role for presentation.	Bias in sample representation (age, religion Christian)<br>Sampling bias	Presentation, acceptance
Agbokey et al., 2019	Tertiary clinic, Ashanti Region	Qualitative descriptive/exploratory	20 patients / 8 caregivers with breast cancer<br>5 health workers<br>2 herbalists	Semi structured in-depth interviews, purposive sampling, thematic analysis	Breast cancer health seeking behaviours	- financial barriers, including prioritising family costs and frustration at poor coverage by NHIS<br>- family support to secure funds<br>- lack of information<br>- beliefs influenced by husbands and family<br>- husbands role in financial decisions<br>- seeking alternative medicines, influenced by affordability and family and friends	Clinic setting may cause participants reluctance to speak openly	Presentation, negotiation, acceptance
Salifu et al., 2021	Tertiary clinic, urban region	Qualitative at 2 time points	Men with advanced prostate cancer (n = 23), family caregivers (n = 23), healthcare professionals (n = 12).	Semi structured interviews at 2 time points, purposive sampling, thematic analysis, Social constructivist theory and interpretivism	Home based palliative care for prostate cancer	- selection of alternative medicines influenced by challenges in accessing medicines and perceived poor supervision by health professionals	Limited sample location<br>Researcher interpretation bias	Acceptance

Bonsu et al., 2019	Tertiary clinic, Ashanti Region	Qualitative descriptive/exploratory	11 patients with breast cancer diagnosed at stage II/IV	Semi-structured interviews, purposive sampling, thematic analysis<br>Applying adapted model of health seeking behaviour (Andersen model of total patient delay)	Breast cancer presentation	- poor symptom recognition, considering changes to breasts normal<br>- lack of knowledge on where to seek support<br>-fears and fatalistic perceptions<br>- husbands views<br>- prioritisation of family and work obligations<br>- Misrecognition by health professionals*<br>- Lack of trust in health sector due to perceived mismanagement<br>- spiritual and community held beliefs about cancer led to alternative use (acting at all stages)*<br>- religious leaders supported earlier presentation<br>- some religious messages caused delays<br>- influence of trusted social network (husband, family, religious leaders, health workers in their community, colleagues)	Sample site bias<br>Recall bias	Presentation (predominantly - exceptions*)
Agbeko et al., 2020	Tertiary clinic, Ashanti Region	Qualitative descriptive (deductive)	15 women presenting with stage III/IV breast cancer	Semi-structured interviews, purposively sampling, deductive thematic analysis with priori themes (Andersen Behavioural Model of Health care utilisation)	Breast cancer presentation	- financial barriers to referral process<br>- poor symptom recognition, not regarded as serious, influenced by community beliefs<br>- prioritisation of family roles and finances, influenced by gender identity and roles beliefs<br>- referral delays when first contact not a specialist	Recall bias<br>Unable to follow-up participants for respondent validation<br>Researcher and interviewer bias	Presentation, negotiation, acceptance
Sanuade et al., 2021	Tertiary clinic, Accra Region	Qualitative descriptive (deductive and inductive - exploratory)	20 women who have commenced treatment	4 focus group discussions, purposive sampling following survey, deductive and inductive thematic analysis	Breast cancer treatment delays	- high cost of treatment regardless of social economic status (leading using alternatives)<br>- fear of losing female identity<br>- fear of treatment centre trauma (lack of institution trust)<br>- husbands opinion<br>- impolite treatment by health professionals and perceived corruption<br>- health professionals advising alternative treatments<br>- patients perceived health facility delays including waiting for biopsy results, medicines and workforce shortages, machines breaking<br>- spiritual beliefs led to seeking alternatives, influenced by cost and others (religious leaders, health workers)<br>- community network opinions	Low number of participants	Negotiation and acceptance

7

Atobrah et al., 2012	Tertiary clinic, Accra Region	Qualitative (descriptive)	9 young Ga adults (25-35) with chronic diseases; cancer of the breast, cancer of the ovaries, cancer of the nasopharynx, cancer of the cervix, stroke and chronic renal failure	Multiple narrative interviews and observations (12 each) Purposive sampling (on socio-economic status) Thematic analysis using symbolic interactionism and grief frameworks	Early onset of chronic diseases	- poor symptom recognition given young age<br>- advise to seek alternative therapies from friends/family<br>- misdiagnosis<br>- community beliefs (including of spiritual causes) led to seeking alternative treatments<br>- social networks and community members influenced beliefs and choices	-	Presentation, negotiation, acceptance
Asobayire et al., 2015	(District hospital, unclear) Kassena-Nankana (Navrongo, Upper East Region)	Qualitative (descriptive, inductive)	10 community women (six farmers, two traders and two teachers - aged 25 -56)	Focus group interviews and documentary analysis of current practices, purposive sampling (not stated) for 10 settlements and differing community and socioeconomic status, thematic analysis following an inductive analytical framework	Breast cancer perceptions	- local dialect disease name translation causes misunderstanding<br>- husbands and community leader roles in decision making<br>- community beliefs about the efficacy of traditional medicines and spiritual beliefs lead to alternatives being sought first<br>- potential for positive influence of religious and community leaders and female groups, if engaged	-	Presentation (predominantly)
Iddrisu et al., 2021	Three hospitals in Accra (regional, military and university)	Qualitative (descriptive exploratory)	12 young patients (15-49 year) with breast cancer	Semi structured interviews, purposive and snowball sampling, thematic analysis	Breast cancer socio economic impact	- financial barriers to treatment (including as NHIS did not cover costs)<br>- Negative misbeliefs about treatment, influenced by friends, family and community gossip<br>- spiritual beliefs held in the community led to seeking alternatives, influenced by friends and family	-	Acceptance
Ayandipo et al., 2020	With 3 country analysis: 3 tertiary clinics, four regional, four district facilities (1 private)	Mixed methods cross sectional study	Stakeholders in NCD programme, MoH and CSOs Heads of the selected facilities or units and cancer specialists (120 health workers in Ghana, 10%	Standardised questionnaire, desk based literature review, Thematic analysis Gap analysis using WHO framework for health system strengthening with the 6 pillars	Gaps in cancer control	- patient unaffordability of treatment due to lack of coverage on the NHIS<br>- neglect by families<br>- misdiagnosis, improper documentation and staff shortages, particularly outside of major tertiary centres<br>- shortages of medicines, commodities, and machine breakdown<br>- patient preference for traditional medicines	-	Negotiation and acceptance

			oncology qualified)					
Nartey et al., 2018	Tertiary clinics in Accra and Ashanti region	Quantitative (retrospective observational)	1725 confirmed invasive cervical cancer cases	Retrospective review of patient record, hospital population sampling, χ2 and logistic (multivariate)regression of factors influencing stage of diagnosis	Cervical cancer stage at diagnosis	- treatment non acceptance for financial reasons<br>- marital status (widowed, divorced, other), ethnicity and (increased) age associated with late diagnosis	Inconsistency, errors and missing data in record keeping<br>Difficulties following up patients/family	Presentation (predominantly)
Scherber et al., 2014	Tertiary clinic in Ashanti region	Quantitative (retrospective observational)	597 breast cancer patients	Review of patient records hospital population sampling, uni and multivariate logistic regression of factors influencing complete treatment follow up	Breast cancer incomplete follow up	- weak positive trend between age and stage diagnosis<br>- no differences in presentation stage whether attended clinic directly or referred from elsewhere<br>- 12.2% patients sought alternative therapies although this does not explain high rates of treatment interruptions/incompletion (73.1% loss to follow up)<br>- presence of hormone status report associated with complete treatment follow up, inferred to reflect the capacity to pay for tests and thus treatment	Inconsistencies with record keeping and missing data Reported barriers only known from those who return for treatment (those who report barriers were more likely to complete treatment)	Acceptance (predominantly, also presentation)
Asamoah et al., 2018	Tertiary clinic in Accra region	Quantitative (retrospective cohort)	1,074 patients with prostate cancer	Retrospective review of records, hospital population sampling, descriptive statistics (χ2 and Fisher's exact tests, Mann-Whitney U tests) and survival analysis	Prostate cancer treatment	- higher median age for presenting with advanced disease	Missing data<br>Hospital records excluded those not able to access	Presentation
Yamoah et al., 2013	Tertiary clinic in Accra region	Quantitative (retrospective cohort)	379 patients with prostate cancer	Retrospective review of patient history, hospital population sampling, descriptive statistics, non-parametric significance tests (due to data skew), survival analysis	Prostate cancer treatment	- treatment non acceptance due to direct and indirect costs being unaffordable, fear of radiation therapy and spiritual beliefs<br>- age was not significantly associated with diagnosis stage	-	Acceptance

9

Dedey et al., 2016	Tertiary clinic in Accra region	Quantitative (retrospective observational)	205 treated breast cancer patients	Retrospective review of records and patient questionnaire, all patients treated (May - Dec 2013), descriptive statistics, non-parametric tests, Poisson multivariate regression on wait time	Breast cancer waiting time	In a multivariate Poisson regression wait time increased with:<br>- low-income low education, low income, Akan ethnicity, aged 50 or over, marital status (single) but not religion<br>- biopsy time<br>- being insured on NHIS (inferred to be due to delays in recouping funds)<br>- Perceived receipt of adequate information, other perceptions of health workers and service were not significant	Sample representation bias<br>Recall bias	Negotiation
Brinton et al., 2017	Three tertiary clinics in Accra and Ashanti region	Quantitative (retrospective observational)	1,184 patients with malignant breast cancer	Retrospective review of case control study data through structured patient interview, factors influencing tumour mass assessed in multivariate parametric model	Breast cancer diagnosis	In a multivariate model, larger mass at diagnosis correlated with:<br>- low education<br>- marital status (widowed/divorced)<br>- seeking assistance from someone other than a doctor or nurse<br>- use of traditional medicines<br>travel and financial difficulties in seeking care were not significant	Limited sampling catchment may limit possible impact of treatment distance	Presentation
Obrist et al., 2014	Tertiary clinic in Ashanti region	Quantitative (Retrospective case control)	141 women/next of kin with breast cancer	Retrospective review of medical records and semi structured interviews, purposive sampling, statistical analysis by parametric t-tests and non-parametric univariate analysis, multivariate model accounting for confounders using logistic regression	Breast cancer treatment incompletion	- non completing patients were more likely to believe they will not respond, but this was not significant in the LR model<br>- practicing Islam was a significantly higher in non-completing group<br>- age was not significant<br>- explored health systems factors were not significant predictors of completion<br>- seeing a traditional healer after any visit was a significant predictor of not completing after the combined analysis<br>- more non completers believed a traditional healer was better at managing cancer (significant at 10% level)<br>- Understanding what the NHIS cover for breast cancer included was significant predictor of completing treatment	Missing contact information and patient follow up<br>Recall bias by next of kin (more likely in DNC group)	Acceptance
Twahir et al., 2021	Two tertiary clinics in Accra region (as part of a study across	Quantitative (retrospective observational)	299 breast cancer patients undergoing treatment	Retrospective review of patient records in standard collection framework, hospital population sampling, descriptive statistical analysis	Breast cancer access to care	Paying out of pocket costs was associated with receiving more treatment cycles	-	Negotiation and acceptance

	Ghana, Kenya and Nigeria)							
O'Brien et al., 2011	Community setting, Ashanti region	Quantitative (descriptive)	42 Traditional Medicine Practice Council (TMPC) registered traditional practitioner	Semi-structured interviews, convenience sampling, descriptive analysis	Traditional herbalists' role in cancer management	Traditional herbalists perceive patient barriers to access include:<br>- cost<br>- lack of knowledge of cancer and fear of treatment<br>- distance and insufficient numbers of health centres<br>- stigma<br>Traditional herbalists poor knowledge of cancer and treatment, and reluctance to refer to other services prevent patients receiving timely care<br>This is influenced by other health professionals' reluctance to collaborate and lack of integration into the health system	-	Presentation, negotiation, acceptance (not specified)
Dadzie et al., 2017	Tertiary clinic in Accra region	Quantitative (retrospective observational)	70 vulva cancer cases	Retrospective review all hospital cases, hospital population sampling, descriptive statistics	Vulva cancer treatment	Cost influenced patient choice (other access barriers were inferred)	-	Acceptance (other access barriers were inferred)
Dunyo et al., 2018	Oncology clinic, Christian hospital (Battor), Volta Region	Quantitative (retrospective observational – cross-sectional analytical)	157 cervical cancer patients	Retrospective review all hospital cases, hospital population sampling, descriptive statistics, non-parametric significance tests, logistic regression of influences on late presentation	Cervical cancer presentation	Education (low) was a significant predictor of late presentation<br>Age was not significant	Missing data, retrospective nature prevented follow up	Presentation
Yarney et al., 2013	Tertiary clinic in Accra region	Quantitative (cross-sectional descriptive)	98 cancer patients	Questionnaire, convenience sampling, descriptive analysis	CAM use in cancer patients	Comparatively high cost of conventional treatment meant only CAM treatment was feasible<br>Lower age, decreased education and being married made it more likely to use CAM in logistic regression model, CAM use influenced by friends and family	Non probabilistic sampling<br>Single institution sample<br>Low response rate<br>Sample bias: excluded those who use CAM but not attending hospital	Acceptance

11

Mburu 2021	Tertiary clinic, Ashanti Region	Qualitative (empircal phenomenological approach)	31 women with breast cancer	Purposive sampling, semi-structured interviews, deductive coding approach	Breast cancer pathway	Misinterpretation of symptoms<br>Initial symptom management with OTC medicines or waiting to see if it improves<br>Women disclosed to friends and relatives, most advised to go to the hospital, but some advised to wait and monitor or see a traditional healer<br>Misdiagnosis and Complex multiple stage referral process led to delays<br>Initial and later stage management through traditional and faith-based healing	Selection bias (not able to include those who did not attend hospital)<br>Remembering of participants and recall bias	Access, negotiation and acceptance
Hobenu and Naab 2022	Teriary clinic, Accra Region	Qualitative (exploratory)	15 Women with cervical cancer	Purposive sampling, in depth interviews, thematic analysis	Cervical cancer from diagnosis to accessing treatment	Misinterpretation of symptoms led to delays in identifying at services<br>Husbands, relatives and friends advised to use herbal treatments<br>Misdiagnosis and Delays due to waiting a long time to get results, having to go to many hospitals to get diagnosed and rescheduling of appointments	Small sample and purposive sampling leading to selection bias	Access and negotiation
Agyemang 2021	Tertiary clinic, Ashanti Region	Qualitative (ethnographic approach)	31 breast cancer patients (up to stage 3), relatives, nurses and doctors observed and 29 took part in interviews	Purposive (max variation) sampling, 2 time point observations, interviews, thematic analysis	Breast cancer formal diagnosis to treatment	Deliberate miscommunication to stop drop out<br>Lack of honest discussion about fertility concerns meant patients fears were not addressed and consequently led to loss to follow up<br>Lack of support around financial issues<br>Costs were barrier to adherence in HCP opinion	Selection bias (not able to include those who did not attend hospital)<br>Small sample	Acceptance

Supplementary Table 3: Evidence map using socio ecological model and candidacy as a framework of access to care

Author and year	Design	Location	Setting	Cancer	Intrapersonal	Interpersonal	Institutional	Community	Policy
Agbeko et al., 2020	Qualitative	Kumasi	Clinic	Breast	A, B	A, C	A, B	A	
Agbokey et al., 2019	Qualitative	Kumasi	Clinic	Breast	A, B, C	A, C	B, C	A	
Agyemang 2021	Qualitative	Kumasi	Clinic	Breast			C		C
Asamoah et al., 2018	Quantitative	Accra	Clinic	Prostate	A				
Asobayire et al., 2015	Qualitative	Upper East	Community	Breast	A	A	B	A	
Asoogo et al., 2015	Qualitative	Kumasi	Clinic	Breast	A	A, B		A	
Atobrah et al., 2012	Qualitative	Accra	Clinic	Multi	A, C	A	B	A, C	
Ayandipo et al., 2020	Mixed methods	Multi-site	Multi-site (health facility)	Multi	B, C	B, C	B, C		C
Aziato et al., 2015	Qualitative	Accra	Clinic	Breast	C	C	B	C	
Binka et al., 2019	Qualitative	Volta	Community	Cervical	A	A, C	A, B, C	A	A, C
Bonsu et al., 2019	Qualitative	Kumasi	Clinic	Breast	A, C	A	A, B, C	A, B, C	

Brinton et al., 2017	Quantitative	Accra / Kumasi	Clinic	Breast	A	A	A	A	
Dadzie et al., 2017	Quantitative	Accra	Clinic	Vulva	C		C		
Dedey et al., 2016	Quantitative	Accra	Clinic	Breast	B		B		
Dunyo et al., 2018	Quantitative	Volta	Clinic	Cervical	A				
Hobenu and Naab 2021	Qualitative	Accra	Clinic	Cervical	A	A	B		
Iddrisu et al., 2021	Qualitative	Accra	Clinic	Breast	C	A	C	C	C
Martei et al., 2018	Qualitative	Accra	Clinic	Breast	A, C	A, C		A, C	C
Mburu 2021	Qualitative	Kumasi	Clinic	Breast	A	A	B	A,C	
Nartey et al., 2018	Quantitative	Accra / Kumasi	Clinic	Cervical	A	A		A	
O'Brien et al., 2011	Quantitative	Kumasi	Community	Multi	A, B, C		B, C	C	C
Obrist et al., 2014	Quantitative	Kumasi	Clinic	Breast	C	C	C	C	C
Salifu et al., 2021	Qualitative	Kumasi	Clinic	Prostate			C	C	
Sanuade et al., 2021	Qualitative	Accra	Clinic	Breast	C	C	B, C	C	
Scherber et al., 2014	Quantitative	Kumasi	Clinic	Breast	A, C		A, C	C	
Twahir et al., 2021	Quantitative	Accra	Clinic	Breast			B		C

Yamoah et al., 2013	Quantitative	Accra	Clinic	Prostate	A, C				
Yarney et al., 2013	Quantitative	Accra	Clinic	Multi	C		C	C	

Key: A= candidacy stage ACCESS, B= candidacy stage NEGOTIATION, C= candidacy stage ACCEPTANCE
