# Supplementary material for: What influences cancer treatment service access in Ghana? A critical interpretive synthesis
Source: BMJ Open. 2022 Oct 5;12(10):e065153. doi: 10.1136/bmjopen-2022-065153 (PMC9535186; doi:10.1136/bmjopen-2022-065153)
Supplement: Author's manuscript [file bmjopen-2022-065153.draft_revisions.pdf]

BMJ Open

BMJ Open is committed to open peer review. As part of this commitment we make the peer review history of every article we publish publicly available.

When an article is published we post the peer reviewers' comments and the authors' responses online. We also post the versions of the paper that were used during peer review. These are the versions that the peer review comments apply to.

The versions of the paper that follow are the versions that were submitted during the peer review process. They are not the versions of record or the final published versions. They should not be cited or distributed as the published version of this manuscript.

BMJ Open is an open access journal and the full, final, typeset and author-corrected version of record of the manuscript is available on our site with no access controls, subscription charges or pay-per-view fees (http://bmjopen.bmj.com).

If you have any questions on BMJ Open's open peer review process please email info.bmjopen@bmj.com

BMJ Open

What influences cancer treatment service access in Ghana? A critical interpretive synthesis

Journal:	*BMJ Open*
Manuscript ID	bmjopen-2022-065153
Article Type:	Original research
Date Submitted by the Author:	30-May-2022

Complete List of Authors:	Tuck, Chloe; The University of Sheffield, School of Health and Related Research (ScHARR)<br>Akparibo, Robert; The University of Sheffield, School of Health and Related Research (ScHARR)<br>Gray, Laura; The University of Sheffield, School of Health and Related Research (SCHARR)<br>Aryeetey, Richmond; University of Ghana, Population Family and Reproductive Health<br>Cooper, Richard; The University of Sheffield, School of Health and Related Research (ScHARR)

Keywords:	Health economics < HEALTH SERVICES ADMINISTRATION & MANAGEMENT, PUBLIC HEALTH, Adult oncology < ONCOLOGY, Health policy < HEALTH SERVICES ADMINISTRATION & MANAGEMENT

SCHOLARONE™
Manuscripts

I, the Submitting Author has the right to grant and does grant on behalf of all authors of the Work (as defined in the below author licence), an exclusive licence and/or a non-exclusive licence for contributions from authors who are: i) UK Crown employees; ii) where BMJ has agreed a CC-BY licence shall apply, and/or iii) in accordance with the terms applicable for US Federal Government officers or employees acting as part of their official duties; on a worldwide, perpetual, irrevocable, royalty-free basis to BMJ Publishing Group Ltd ("BMJ") its licensees and where the relevant Journal is co-owned by BMJ to the co-owners of the Journal, to publish the Work in this journal and any other BMJ products and to exploit all rights, as set out in our licence.

The Submitting Author accepts and understands that any supply made under these terms is made by BMJ to the Submitting Author unless you are acting as an employee on behalf of your employer or a postgraduate student of an affiliated institution which is paying any applicable article publishing charge ("APC") for Open Access articles. Where the Submitting Author wishes to make the Work available on an Open Access basis (and intends to pay the relevant APC), the terms of reuse of such Open Access shall be governed by a Creative Commons licence – details of these licences and which Creative Commons licence will apply to this Work are set out in our licence referred to above.

Other than as permitted in any relevant BMJ Author's Self Archiving Policies, I confirm this Work has not been accepted for publication elsewhere, is not being considered for publication elsewhere and does not duplicate material already published. I confirm all authors consent to publication of this Work and authorise the granting of this licence.

For peer review only

What influences cancer treatment service access in Ghana? A critical interpretive synthesis

Chloe Tuck[1], Robert Akparibo[1], Laura Gray[1], Richmond Aryeetey[2] , and Richard Cooper[1]

1. School of Health and Related Research, University of Sheffield, Sheffield, UK
2. School of Public Health, University of Ghana, Legon, Accra, Ghana

Corresponding author:

Chloe Tuck, School of Health and Related Research, University of Sheffield, 30 Regent Street, Sheffield, S1 4DA, UK

Chloeztuck@gmail.com

Cztuck1@sheffield.ac.uk

Phone: +447402110248

Word count: 4787

Keywords: critical interpretive synthesis, Ghana, access to cancer treatment, socio-ecological model, health policy

For peer review only

ABSTRACT

Objectives: Multiple social-cultural and contextual factors influence access to and acceptance of cancer treatment in Ghana. The aim of this research was to assess existing literature on how these factors interplay and could be susceptible to local and national policy changes.

Design: This study uses a critical interpretive synthesis approach to review qualitative and quantitative evidence about access to adult cancer treatment services in Ghana, applying the socio-ecological model and candidacy framework.

Results: Our findings highlighted barriers to accessing cancer services within each level of the socio-ecological model that are dynamic and interacting. Evidence was lacking in relation to determinants of treatment non-acceptance across all cancers and in the most vulnerable societal groups due to methodological limitations.

Conclusions: Future policy should prioritise multi-level approaches to improve the quality and affordability of medical care while harmonising linkages with traditional and complementary care systems. Research should seek to overcome methodological limitations to understand the determinants of accessing treatment in the most vulnerable populations.

Strengths and limitations of this study

- A strength of this study is the combination of purposive and systematic searches, and the reflexive approach to developing the search strategy which enabled it to cover a wide range of articles.
- Additionally, the critical interpretive synthesis involved a critique of the literature to identify methodological limitations and research gaps.
- However, as only published academic articles are included in this study, it may overlook other forms of evidence, including locally generated and day-to-day working understandings.
- The interpretation of the evidence will reflect the inherent biases in world view of the lead author.

KEY QUESTIONS

What is already known on this topic

Multiple social-cultural and contextual factors influence access to and acceptance of cancer treatment in Ghana. How these factors interplay and could be susceptible to policy changes is unknown.

What this study adds

This study highlights barriers to accessing cancer services within each level of the social-ecological model that are dynamic and interacting. There is a gap in understanding determinants of treatment non-acceptance across all cancers and in the most vulnerable societal groups.

How this study might affect research, practice or policy

Findings of this review have potential to impact future policy formulation, which should prioritise multi-level approaches to improve the quality and affordability of medical care whilst harmonising linkages with traditional and complementary care systems.

For peer review only - http://bmjopen.bmj.com/site/about/guidelines.xhtml

INTRODUCTION

Cancer is a growing burden in low and middle income countries (LMICs)(1,2). Despite efforts by the World Health Organisation (WHO) to prioritise tackling cancer inequity, hurdles remain due to the limited evidence to inform cost-effective decision-making and the high expense of cancer control(1). In Ghana, cancer treatment is focused in large referral centres in major cities, with disparity in resources and health worker expertise in rural areas, and limited coverage of the National Health Insurance Scheme (NHIS)(3–7). Policy efforts to expand cancer services are further hindered when patients prioritise traditional alternatives over orthodox cancer services(8). Multiple social-cultural, economic and health system factors can influence how patients access, navigate, and choose suitable cancer care in Ghana(3). In addressing this, there is a relative lack of public health surveillance data. There have been recent attempts to reconcile this(9) but a comprehensive understanding remains elusive. One alternative approach is to consider the relevance of theory.

Socio-economic model and candidacy framework perspectives on cancer treatment access

One important way of understanding the complexity and various factors previous described in relation to cancer treatment service access is to consider this in terms of the socio-ecological model (10). This has been used in many settings to map barriers to healthcare engagement from a systems perspective(11,12). The socio-ecological model considers the individual within an ecosystem of intrapersonal, interpersonal, community, health organisational, and policy influences. This has been applied extensively to map systems factors since it was developed by Bronfenbrenner(10), including health behaviours in several African settings(11,12). The process of an individual accessing cancer services is dynamic and delays in access can occur at multiple stages. Patients may experience barriers presenting at services, negotiating the care pathway, being offered and accepting treatment. A Critical interpretive synthesis (CIS) exploring health access in the UK(13) highlighted that this involves dynamic interactions between the individual, the health environment and health professionals. The term 'access' often overlooks this dynamism, whilst terms such as 'uptake' provide a narrow view that overlooks patient demand and service navigation afterwards.

The candidacy framework provides a multi-stage interacting process of patient access holistically. 'Service use' may overlook the complex process of navigating care and those who do not seek, choose or are refused services(14). For example, negotiation of services is overlooked in asylum seekers and refugees(14). Van der Boor and colleagues describe candidacy in two broad stages 'access' (identification of eligibility, navigation of services) and 'negotiation' (permeation of services, appearance, adjudications, offers and resistance, and dynamic interactions with local services)(14). Candidacy has been applied to understand patient-doctor interactions influencing cancer health seeking behaviours(15) and in an African context(16).

This paper builds on the issue of factors relating to cancer treatment service access in LMIC settings such as Ghana by presenting the findings of an evidence review that was informed by theory. The primary aim was to systematically review and critique literature from a systems perspective to understand factors influencing cancer treatment service access in Ghana. A further aim was to assess the strengths and limitations of methods associated with existing research relating to this topic.

METHODS

Using the RETREAT framework(17), critical interpretive synthesis (CIS) was considered to be the most suitable approach(13). CIS has been used in a variety of policy and health service settings(18–20) and

For peer review only - http://bmjopen.bmj.com/site/about/guidelines.xhtml

combines systematic and purposive approaches to identify multiple types of evidence and identify themes following an evidence critique. This involves considering how the problem has been constructed, underlying assumptions and epistemology, and how this has influenced the methodology and conclusions(13).

Search strategy

The search strategy to find articles on access of adult cancer treatment in Ghana was developed using the question framework PerSPECTiF(21). Access was defined holistically through the candidacy framework(13). The setting included all levels of the socio-ecological model to provide a systems perspective. This was tested through pilot searches of each potential source and involved three data sources: firstly, primary systematic searches were undertaken in Medline (via OVID), Web of Science, CINAHL and African Index Medicus. Secondly, additional relevant individual journals were identified, and targeted searches performed. Thirdly, reference and citation searches of articles identified through primary searches were performed. Search terms were composed of multiple equivalent thesaurus terms and phrases to cover three elements: Ghana, health service access/uptake of services, and cancer. The lead author (CT) screened all titles and abstracts using an agreed inclusion criteria whilst other authors conducted quality checks for consistency. Selected abstracts were read in full; data was extracted alongside to facilitate decision making and an audit trail (See data extraction in supplementary material). Initial screening highlighted ambiguity in the screening criteria, which was further refined to ensure consistency prior to formal selection. Inclusion criteria included only primary research conducted with a 10-year time frame to align with Ghana's increased policy interest in efficiently expand national health insurance packages(6). Initial screening highlighted the need for a focused exclusion criteria which was again informed by the question framework PerSPECTiF(21).

Critical appraisal and data synthesis

In line with the CIS, articles were not favoured based on quality alone but contribution of rich insights. The CIS deals with weak evidence through including a critique of methods and approach. A streamline critical appraisal for major and methodological flaws was conducted similar to Dixon Woods et al.,(13). The quality of quantitative and qualitative findings was assessed in terms of reliability and trustworthiness(22). A framework was developed to extract data on study characteristics whilst cross-tabulating study findings against the socio-ecological model(10) and the candidacy framework(13). The data extraction template was reviewed and refined before full extraction. Data was collected as line of arguments(13). First order constructs (taken directly from the data in articles) and second order constructs (author reports from articles) were extracted and separately noted within the framework. It was noted where study authors made further (secondary) inferences and assumptions from data that were not primary findings, but relevant to the themes. Researchers' limitations were recorded. The data were segregated into qualitative, quantitative, and mixed methods studies and each interpreted qualitatively. A synthesising argument(13) was applied to map first and second order findings across studies to interpret the evidence and create new concepts that draw on the whole body of evidence (third order/synthetic constructs). Inferences were mapped across the studies to enable the body of evidence to be critiqued by research question construction, methods, and conclusions and how these fit into the general findings, to identify trends in the literature and limitations with current approaches. To bring together themes in candidacy, the candidacy framework was summarised into three main stages. Van der Boor use two stages,(14) however, as themes were identified, it was noted that treatment acceptance and the interactions around this over time play a pertinent role in the patient pathway in Ghana. Thus, the adapted three stage model also notes the

For peer review only - http://bmjopen.bmj.com/site/about/guidelines.xhtml

importance of the dynamics of treatment acceptance. As part of the synthesis process, the primary literature from the data extraction were revisited and reinterpreted with emerging evidence to ensure critical details or limitations were not missed.

Patient and Public Involvement statement

No members of the public or patients were involved in this research.

RESULTS

Search results

Systematic searches on 4 databases and 6 journal searches identified 312 citations in March 2021. After duplicate removal, 203 potentially relevant abstracts were screened, subsequently 78 articles were identified for full text screening. A further 16 abstracts were identified for full text screening following citation and reference searching. The four database searches were repeated to identify new publications in March 2022. Twenty-eight articles were selected for inclusion as shown in PRISMA diagram (Figure 1).

A summary of the articles reviewed is included in Supplementary Material Table 1. Applying the candidacy framework and socio-ecological model, key themes were identified and are presented below. The evidence mapping table (Supplementary Material, Table 2) highlight the candidacy stage and level within the socio-ecological model that the articles addressed, as judged by lead author.

Accessibility defined through a 'candidacy' lens

The candidacy framework proved valuable in assessing how healthcare access has been approached, from a holistic perspective. Treatment acceptance was a key emerging issue where there was a gap in understanding. Within treatment acceptance there were multiple aspects – delays accepting, interruptions, choosing alternatives and incompletion or loss to follow up, non-compliance, refusal for referral and non-acceptance of diagnosis. This was a dynamic process.

Although the full candidacy process has been considered in research, there were key gaps in how it had been approached and some aspects warranted further exploration. Seven studies aimed to explore delays with initial contact with cancer services on identification of need (presentation), yet the reasons for eventual non-presentation could not be explored in most studies that were clinic based, as all patients surveyed eventually reached treatment centres. Two studies quantified acceptance as having complete follow up and treatment completion. A further four noted delays and high non-acceptance/loss to follow up but did not formally explore them at an individual psychosocial level as this was not within the research aims. Several qualitative studies explored individual barriers to accepting care. In some this was not the primary focus but emerges in the findings. As these were sampled from a clinic, they represented patients who eventually (despite delays or interruptions) accessed treatment, so the enablers and barriers in those who ultimately dropped out was unknown. Therefore, in depth qualitative psychosocial information on treatment incompletion was not collected. Although the literature on acceptance was predominantly breast cancer related, there was some limited evidence it occurred in other cancers, but the extent and reasons for this were not explored.

Barriers and enablers of cancer service access interpreted through the socio-ecological model

The findings on enablers and barriers to candidacy for cancer treatment were mapped using the socio-ecological model to consider the Ghanaian health ecosystem. These are summarised in Figure 2.

For peer review only - http://bmjopen.bmj.com/site/about/guidelines.xhtml

Intrapersonal

Financial barriers

Inability to afford treatment was reported as a barrier leading to delays in, and non-acceptance of care. This was also noted by traditional herbalists(23) and health workers(3). Yamoah(24) found it encompassed socioeconomic factors, travel costs and lost work. Although two studies indicated it was a greater barrier in those from a low income,(25,26) Sanuade(27) suggested this was regardless of socioeconomic status. Four studies(26–29) demonstrated that high cost of medical treatment led to use of herbal and traditional alternatives. Prioritisation of finances on family led to delays in accepting treatment (29–31). Financial barriers impacted negotiation of care(30); those from a lower income were more likely to experience longer wait times(32).

Fears and beliefs about treatment and its outcomes

Obrist(33) found patients who believed in the efficacy of treatment were more likely to complete treatment in single variable analysis, although this was not significant in multivariate models where potential confounding factors were controlled for. Fears and beliefs pose individual level barriers from qualitative finding. This included fear of treatment, medicines, and the outcomes(8,26,27,31,34). For example for breast cancer, loosing breasts, womanhood, female identity(26,27,31). Fears around institutional trauma suggested lack of trust health facilities(27). Healthcare professions deliberately miscommunicated to avoid patient fear and drop out(35).

Misunderstanding about cancer signs and symptoms

Lack of knowledge about cancer signs and symptoms led to delays in seeking medical treatment(8,26,30,31,36–38). With breast cancer, lumps were not regarded as serious when painless and sometimes considered part of normal tissue(8,26,30,31). This was influenced by beliefs held in the community and within patients' social networks(8,30,36). Lay beliefs were influenced by the terminology used for breast cancer in the local dialect, which led to poor understanding(39).

Social demographics associations

Evidence was conflicting as to whether age, religion and ethnicity impacted stage of diagnosis, wait times and treatment completion, which may associate differently in different cancers given different demographics and natural histories(24,32,33,40–43). As ethnicities often cluster predominantly in different regions, the potential confounding of local health system and environmental should be checked in future studies. Some evidence indicates that lower education is associated with the presenting of larger tumour masses(43,44) and waiting longer for treatment(32), but this was not a consistent influencer, and whether it was associated with treatment acceptance was not explored. One study exploring treatment pathways for young people with chronic diseases suggested community beliefs were more influential than educational status(36). Another found that community beliefs and norms influenced the perceptions of breast cancer regardless of socio-economic status(39). Despite clear financial barriers to treatment; there was no evidence that income status and occupation were associated with presentation and acceptance of treatment, but low income status was associated with increased wait times in one study(32).

For peer review only - http://bmjopen.bmj.com/site/about/guidelines.xhtml

Interpersonal

Marital relationships influence treatment seeking

Qualitative evidence in female cancer suggests husbands influence their wives' treatment seeking behaviours and acceptance,(8,25,27,30,31,38,39,45) by controlling financial decisions about treatment.

Family prioritisation delayed treatment

Women prioritised other activities linked to their economic, family and social roles, such as working for more money, treating children, and paying school fees. These lead to delays presenting for, negotiating and accepting treatment(8,26,29–31). Caring for others meant patients put the needs of others first, neglecting their own health. This aligned with findings on the unaffordability of treatment.

Close support networks influence treatment access

Patients understanding about cancer, its causes, and how they engage with care was influenced by close friends and family(8,26,29,34,36–38,45). Misinformation could lead to late presentation, delay help seeking, and use of alternatives(8,29,36,37). For women, lack of husband, family and friend support delayed treatment seeking(25,45). Familial financial support was an enabler for some to seek treatment(29,45) and neglect without it may impede access(3).

Institutional

Healthcare personnel as gatekeepers to medical and alternative care

Poor detection at primary health facilities, community pharmacy and private settings, may have delayed diagnosis(3,8,29,30,36,44–46). Some women sought OTC meds for pain management(37). Seeking assistance from someone other than a nurse or doctor was associated with a larger mass at diagnosis for breast cancer, which could include a diverse mix of pluralist and community supports(44). Some health professionals also advised herbal alternatives, delaying medical cancer treatment(27). A mixed method study found an inability for facilities to diagnose cancer, improper documentation and filing of patient folders and workload – likely exacerbated by a shortage of healthcare workers trained in oncology outside of major tertiary centres(3). In agreement, qualitative studies with patients found misdiagnosis was common(37,38). There were delays due to the complex referral process, waiting a long time to get results, having to go to many hospitals and laboratories to be diagnosed, and consultant rescheduling(37,38). Delays between referral and starting radiotherapy were suggested to be due to resource availability, whilst irregular medicine supply meant patients had to source medicine outside hospitals at high cost(29,47). Patients showed negative perceptions of the care system and professionals(25,27,27,45). Patients perceived treatment delays due to workforce shortage, hospital machines breaking, and medicines shortages(27). These beliefs appear to contribute to a lack of confidence and trust in the health system. Non-completing patients were more likely to harbour negative views such as that the unavailability of cancer medicines delayed their treatment(33). Doubts in the efficacy and disappointment with conventional treatment created barriers to seeking treatment(25) and influenced use of pluralistic treatments(28). Fear of radiation led some not to receive clinically recommended treatment(28).

Community

The body of literature showed the strong role community beliefs and norms played in shaping access to cancer care. These were interconnected with personal perceptions and health system factors.

For peer review only - http://bmjopen.bmj.com/site/about/guidelines.xhtml

Spiritual and traditional beliefs about cancer causes and treatment

In an overwhelming majority of the literature, patients assigned their cancer diagnosis to spiritual causes, which led patients to seek traditional herbal and spiritual treatments, delaying presentation and interrupting medical treatment at multiple stages. This was associated with financial barriers to conventional treatment,(26,27,29) advice from supports such as spouse,(45) health workers,(27) religious messages,(8,27) community networks and beliefs(8,27,30,34,36,39). Additionally alternative therapies were often perceived as more available and acceptable, seen as efficacious(25,26,39,47).

Religiosity plays a diachronous role

Religious beliefs, messages and leaders influenced alternative therapy use(8,27) and caused delays(8,27,37). Yet, religious leaders were identified within patients' trusted support networks(8) and their advice facilitated medical presentation(8,31). Some studies found the church played a supportive role, encouraging women to present at services and providing financial assistance to low-income families(31).

Gender and identity norms

For women with breast cancer, mastectomy was associated with a fear of 'diminished sexuality and femininity' which led many women to delay treatment after seeing an oncologist(26,27,30,31). However, the barriers around identity may differ in other tumours and population groups. Unaddressed fears about fertility loss may have increased drop out (35).

Community networks influenced beliefs and norms

Common misconceptions, beliefs and behaviours held by patients were reinforced by community and social network beliefs(8,27,30,36). Interlinked with community beliefs about cancer is self and socially experienced stigma due to the cause of the disease being spiritual: a curse, mis-endeavour, or the patient being a witch(25,31,39). This led to patients seeking traditional herbal and spiritual treatments, whilst creating shame and secrecy. A retrospective survey found patients who did not complete treatment were more likely to answer they do not know if they were fearful of their community response. However, this likely reflects uncertainty by next of kin respondents, who were substantially higher in 'non-completing treatment' groups(33).

Policy

National Health Insurance Scheme (NHIS) inclusion of cancer care

Based on the financial barriers to treatment reported by patients and healthcare workers, lack of cancer care inclusion within the NHIS was inferred to lead to treatment refusal and delays(3). This was the case for cancers not covered by the scheme(24,42,48), as well as breast(29,31) and cervical cancer(25,40). Breast cancer patients were unanimously frustrated that the NHIS did not cover substantial amounts of treatment and discussed the huge financial burden, especially of chemotherapy drugs(29,31). This meant some women could not start treatment on time(34). This was aggravated by medicines stock-outs,(3,27,29,47) requiring purchase elsewhere at additional cost(29,47). Not being insured was significantly associated with a shorter wait time for breast cancer treatment,(32) which could reflect preferential treatment to those paying upfront due to delays in the administrative process of reimburse NHIS funding. Knowledge of hormone receptor status predicted complete treatment follow up, as this service is offered at a cost(41). Healthcare professionals acknowledged costs were barriers for patients but struggled to broach such topics(35).

For peer review only - http://bmjopen.bmj.com/site/about/guidelines.xhtml

For peer review only

Integration of pluralistic care approaches

Given the prominent roles of traditional, herbal and spiritual care, improved integration with the orthodox health system could improve patient access(23,26).

Thirty eight percent of clinical workers surveyed in Ghana attribute treatment disruptions to traditional medicines use(3). Reported use of traditional healers was a significant predictor of late presentation after other variables were controlled for(44). An assessment of factors associated with treatment completion found visiting a traditional healer was a significant predictor of not completing treatment(33). Although alternative support could be concurrent to orthodox medical intervention, over 50% of complementary and alternative medicine practitioners surveyed indicated they did not let patients seek other care alongside(23) and 63% of customers said they declined orthodox therapy while using such therapies(28). Nevertheless, one study found that although 12.2% seek alternative therapies, this only partially explains high rates(73.1%) of loss to follow up(41). Whilst traditional herbalists are considered health professionals, with some services integrated into the Ghanaian health system, poor knowledge of cancer causes and symptoms, and treatment and reluctance to refer to other services are barriers to providing their patients with timely appropriate care(23). This is influenced by a perceived reluctance collaborate from other health system components(23). Mburu suggests the interaction between treatment approaches is non-linear as acts at multiple pathway stages (37).

Critique of the evidence

As part of the CIS approach, a critique of the literature was conducted to identify themes in methods, assumptions, theories, and analysis to identify methodological limitations and research gaps for further studies. A summary of the studies characteristics are displayed in Figure 3.

This review accurately represents evidence in Ghana, which has a high breast cancer contribution. Most studies focus either on presentation delays or treatment interruptions, barriers and treatment delays. Although many studies note treatment incompletion and loss of follow up, only two assess this directly and they note challenges in data collection,(33,41) there are no qualitative studies exploring this. Eighty eight percent (25/28) of studies were based in tertiary treatment clinics in the Greater Accra and Ashanti region, so may not reflect those in other regions. As 89% of qualitative studies sampled purposively from tertiary clinics, this led to biased sampling. They omit those who did not attend, those who dropped out without contact or cannot reach treatment centres. Sampling patients in clinics represents those who eventually presented for and accepted treatment. Barriers could be experienced differently in the under-represented population. As some studies had a small sample and were predominantly Christian, there was limited ethnographic diversity, which may influence generalisability. Findings on the social demographic traits linked to barriers accessing treatment were inconclusive.

Hypothetical inquiry was found to be common across studies. This may lead to error if an individual is not able to accurately predict their behaviour in an unknown situation(49), which may be the case as cancer is stigmatised and not talked about openly(50). Further work should explore and seek to understand the impact cognitive bias may have when using hypothetical situations. The gaps in research identified are summarised in the box below.

For peer review only - http://bmjopen.bmj.com/site/about/guidelines.xhtml

Critiquing the evidence body identified several themes and research gaps:

- Most studies are situated at tertiary clinics, so may not represent rural regions
- Sampling from tertiary clinics means populations who do not present, negotiate the referral pathway from local services and eventually choose treatment are not represented. Understanding what influences access in these missing populations warrants further study
- Treatment drop-out is frequently observed but the reasons for this are not sufficiently explored.
- As most studies focus on breast cancer, there is need to understand the extent of treatment drop out across all cancers and which factors influence this

DISCUSSION

This study used CIS to review multiple types of qualitative and quantitative evidence from literature on access to cancer treatment services in Ghana. Applying 'candidacy' enabled the dynamic and continuous process of accessing, negotiating, and accepting treatment to be explored within the Ghanaian social, economic and policy environment. It highlighted determinants of cancer treatment service access in Ghana are interlinked and within each stage of the socio-ecological model. There is a research gap in understanding the determinants of accessing treatment in the most vulnerable populations due to methodological limitations.

Through this approach, we were able to critique the literature, highlighting trends in methodology and gaps in evidence for future study. The CIS enabled detailed context-specific insights as well as identifying limitations in research approaches, data collection and acquisition challenges to inform future research. A reflective and iterative approach to broaden the breadth of evidence, interpretation and assimilation, was taken. This was particularly valuable to for integrity in research in West African, due to epistemic injustice in how knowledge is perceived and interpreted(51). This is the first study to have explored the applicability of the candidacy framework of healthcare access to a Ghanaian setting. Although this has proven valuable in other African settings,(16) this model was developed in a UK setting, therefore it was important to acknowledge pre-existing bias in perspective and thus to critically assess this framing and how it might impact the interpretation of results(51). Selecting globalised frameworks (over those locally synthesised) can lead to interpretive marginalisation, which we sought to avoid. Only published academic articles were included in this study, which may overlook other forms of evidence, including locally generated and day-to-day working understandings. However, this was minimised through an iterative approach to include multiple databases, targeting local journals and supplementary searches. Another challenge was the vague and broad nature of terms relating to 'accessing' healthcare. There was a need to balance breadth of search with pragmatism to formulate a multi-step search strategy. The interpretation of the evidence was primarily conducted by the lead author, so will reflect their inherent biases in world view(22).

The evidence highlighted financial barriers to cancer treatment access, which interacted with cultural factors and societal influences, such as norms around managing household finances, prioritisation and cultural acceptance of alternative medicines. Globally, catastrophic costs (defined as greater than 40% household effective income) due to non-communicable disease health expenditure are prevalent in LMICs, leading to individuals not taking medicines and impoverishment(52). This exacerbates inequities, having the greatest impact on the poor and leads to detrimental coping strategies(53).

For peer review only - http://bmjopen.bmj.com/site/about/guidelines.xhtml

Poor patient clinician relationships have been found to lead people to seek traditional medicines alternatives in Ghana(62). Traditional medicines practitioners were seen to offer more patient-centric, holistic care which was more comforting. A lack of trusted was noted for orthodox facilities, which may reflect cultural beliefs as well as past healthcare experiences. An evidence synthesis across LMICs found modern medicines viewed to be harmful and ineffective; suspicion and mistrust of biomedicine lengthened delay and led to alternative use, and the impact of this may be exacerbated in the most vulnerable(50). A qualitative exploration of influences on traditional medicines use in Ghana found their 'pull' by accessibility and alignment with cultural beliefs, whereas scepticism of biomedicine may push people from orthodox healthcare(62).

At a community level, spiritual beliefs about the origin of cancer - notions and beliefs typically held in the community can be ascribed as lay explanatory models of disease. In accordance with Kleinman(65), lay models of disease can differ from biomedical models based on social experiences and impact how individuals interpret and act on their condition. Community factors influenced explanatory models for hypertension in rural northern Ghana and impact treatment access(66). Similarly, in this review, explanatory models created stigma leading to secrecy and selection of traditional medicines over biomedical intervention.

At a policy level, two key themes where reforms could improve cancer treatment uptake stood out: 1) greater inclusion of cancer treatments within the NHIS, 2) enhanced integration of traditional medicines to provide complementary options to medical care for cancer.

Despite the NHIS aim of achieving universal health coverage for all, there has been notable disparity, with the lowest coverage concentrated in the poor (73,74). Multidimensional barriers due to poverty, dissatisfaction and distrust of the health service and staff may prohibit enrolment(75). Furthermore, catastrophic spending due to out of pocket costs remains high(76). The NHIS plan seeks to cover a considerable amount of the local disease burden and since its nascence the inclusion list has been revised in response to transitions in disease burden and advancing treatments(77,78). Despite breast and cervical cancer in theory being covered(79), it is widely noted women still face considerable financial burden, which this study further highlights. The burden in men however remains less clearly mapped. The high expense of cancer medicines poses a challenge to decision-makers, who must weight costs and benefits when deciding on how to invest health budgets. Approaches such as the health technology assessment platform recently established in Ghana could help prioritise high-cost medicines(80,81).

Another policy area highlighted was coordination between medical services and traditional medicines. Studies in Ghana have shown traditional medicines users are more likely to be poor and not insured on the NHIS(82). The NHIS currently provides services through a plural system of public, faith-based, governmental and private facilities, and includes some traditional medicines(73). Although lack of harmonisation of traditional medicines with the healthcare system(83) was reported in this study, there have been multiple reforms to this end since the Traditional and Alternative Medicine Directorate was established under the Ghanaian Ministry of Health in 2001(84). Still coordination is hindered by a lack of professional respect from other health professionals(83). Co-current use of alternative forms of care has been found in pregnant women in Ghana. It was highlighted the individual psychosocial and emotional support they provide, which this study found can be lacking

For peer review only - http://bmjopen.bmj.com/site/about/guidelines.xhtml

patient interactions with orthodox medicine, despite being key for candidacy in cancer treatment(15,85).

The African Union has calls to recognise the importance of traditional medicines and a Global Centre for Traditional Medicines has been established in India(86,87). However, there remain challenges in how traditional and complementary medicines are perceived and deemed efficacious. Barry(88) suggests differently constructed modes of evidence are needed for traditional medicines, as scientific evidence through clinical trials offers a reductionist, narrowly defined view of evidence, that overlooks the role of lived-in social experiences, advocating for the 'expanded epistemology of science'(89). Future policies should seek to improve the affordability and quality of publicly provided medical care whilst harmonising with complementary treatments that align with community beliefs.

Future research is needed to address the research gaps identified. First, to understand the extent of treatment non-adherence across call cancers are what individual, social and health system factors impact this. Second, there are methodological limitations in understanding the views of those who do not attend clinics, which may represent the most vulnerable. Researchers should seek approaches to overcome this which are suitable within the local context.

Acknowledgements
This research was funded by the Wellcome [108903/B/15/Z]. Additionally, the authors would like to thank the School of Health and Related Research Library team for their expertise in designing the search protocol, as well as all researcher staff and graduate at the Universities of Sheffield and Ghana who commented or provided feedback on preliminary results.

Funding

This research was funded by Wellcome [108903/B/15/Z].

Competing interests

The authors declare no conflicts of interest in undertaking this research.

Data sharing statement

Data sharing not applicable as this is a review of literature, and no datasets were generated.

Contributorship statement

CT, RAk, LG and RC conceptualised the study. CT led the in designing the study, conducted literature search to collect data, perform the data analysis and wrote the first draft. RAk and LG contributed to selecting literature for inclusion in the study and advised on data collection and analysis. RC, RAk, RAr and LG reviewed the initial and final draft manuscript and made intellectual inputs to improve quality. All authors read and approved the final manuscript.

CT is responsible for the overall content as guarantor and accepts full responsibility for the work and controlled the decision to publish.

The corresponding author (CT) attests that all listed authors meet authorship criteria and that no others meeting the criteria have been omitted.

For peer review only

Ethical approval

This study does not involve human participants and did not require ethical approval

Figure legends / captions

Figure 1: PRISMA

*Italicised numbers are from search update conducted March 2022 to identify newly published literature. Diagram adapted from From: Page MJ, McKenzie JE, Bossuyt PM, Boutron I, Hoffmann TC, Mulrow CD, et al. The PRISMA 2020 statement: an updated guideline for reporting systematic reviews. BMJ 2021;372:n71. doi: 10.1136/bmj.n71. For more information, visit: http://www.prisma-statement.org/

Figure 2: Key influences on candidacy for cancer treatment mapped against the socio-ecological model(10–12)

Figure 3: Summary of characteristics of included studies

References

1. World Health Organization. WHO report on cancer: setting priorities, investing wisely and providing care for all [Internet]. World Health Organization; 2020 [cited 2020 Mar 28]. Available from: https://apps.who.int/iris/handle/10665/330745

2. Bray F, Ferlay J, Soerjomataram I, Siegel RL, Torre LA, Jemal A. Global cancer statistics 2018: GLOBOCAN estimates of incidence and mortality worldwide for 36 cancers in 185 countries. CA Cancer J Clin. 2018;68(6):394–424.

3. Ayandipo O, Wone I, Kenu E, Fasehun LK, Ayandipo O, Gaye F, et al. Cancer ecosystem assessment in West Africa: health systems gaps to prevent and control cancers in three countries: Ghana, Nigeria and Senegal. Pan Afr Med J. 2020;35(101517926):90.

4. Adanu K. Household treatment cost of breast cancer disease and cost coping strategy of women, presenting at the Korle Bu Teaching Hospital [Dissertation]. [Accra]: University of Ghana; 2019.

5. Bonsu AB, Aziato L, Clegg-Lamptey JNA. Living with Advanced Breast Cancer among Ghanaian Women: Emotional and Psychosocial Experiences [Internet]. International Journal of Palliative Care. 2014 [cited 2019 Dec 2]. Available from: https://www.hindawi.com/journals/ijpc/2014/403473/abs/

6. Wang H, Otoo N, Dsane-Selby L. Ghana National Health Insurance Scheme. :71.

7. Blanchet NJ, Fink G, Osei-Akoto I. The Effect of Ghana’s National Health Insurance Scheme on Health Care Utilisation. Ghana Med J. 2012 Jun;46(2):76–84.

8. Bonsu AB, Ncama BP. Recognizing and appraising symptoms of breast cancer as a reason for delayed presentation in Ghanaian women: A qualitative study. PloS One. 2019;14(1):e0208773.

9. Yarney J, Ohene Oti NO, Calys-Tagoe BNL, Gyasi RK, Agyeman Duah I, Akoto-Aidoo C, et al. Establishing a Cancer Registry in a Resource-Constrained Region: Process Experience From Ghana. JCO Glob Oncol. 2020 Apr 17;6:JGO.19.00387.

For peer review only - http://bmjopen.bmj.com/site/about/guidelines.xhtml

10. Bronfenbrenner U. Ecological systems theory. In: Six theories of child development: Revised formulations and current issues. London, England: Jessica Kingsley Publishers; 1992. p. 187–249.

11. The Socioecological Model as a Framework for Exploring Factors Influencing Childhood Immunization Uptake in Lagos State, Nigeria. 2021 Jan 8 [cited 2021 Apr 20]; Available from: https://www.researchsquare.com

12. Ngwenya N, Nkosi B, Mchunu LS, Ferguson J, Seeley J, Doyle AM. Behavioural and socio-ecological factors that influence access and utilisation of health services by young people living in rural KwaZulu-Natal, South Africa: Implications for intervention. PLoS ONE [Internet]. 2020 Apr 14 [cited 2021 Apr 20];15(4). Available from: https://www.ncbi.nlm.nih.gov/pmc/articles/PMC7156071/

13. Dixon-Woods M, Cavers D, Agarwal S, Annandale E, Arthur A, Harvey J, et al. Conducting a critical interpretive synthesis of the literature on access to healthcare by vulnerable groups. BMC Med Res Methodol. 2006 Jul 26;6(1):35.

14. van der Boor CF, White R. Barriers to Accessing and Negotiating Mental Health Services in Asylum Seeking and Refugee Populations: The Application of the Candidacy Framework. J Immigr Minor Health. 2020 Feb;22(1):156–74.

15. Tookey S, Renzi C, Waller J, von Wagner C, Whitaker KL. Using the candidacy framework to understand how doctor-patient interactions influence perceived eligibility to seek help for cancer alarm symptoms: a qualitative interview study. BMC Health Serv Res. 2018 Dec 4;18:937.

16. Adeagbo O, Herbst C, Blandford A, McKendry R, Estcourt C, Seeley J, et al. Exploring People's Candidacy for Mobile Health–Supported HIV Testing and Care Services in Rural KwaZulu-Natal, South Africa: Qualitative Study. J Med Internet Res. 2019 Nov 18;21(11):e15681.

17. Booth A, Noyes J, Flemming K, Gerhardus A, Wahlster P, van der Wilt GJ, et al. Structured methodology review identified seven (RETREAT) criteria for selecting qualitative evidence synthesis approaches. J Clin Epidemiol. 2018 Jul;99:41–52.

18. Vélez CM, Wilson MG, Lavis JN, Abelson J, Florez ID. A framework for explaining the role of values in health policy decision-making in Latin America: a critical interpretive synthesis. Health Res Policy Syst. 2020 Sep 7;18(1):100.

19. Khalid AF, Lavis JN, El-Jardali F, Vanstone M. Supporting the use of research evidence in decision-making in crisis zones in low- and middle-income countries: a critical interpretive synthesis. Health Res Policy Syst. 2020 Feb 18;18(1):21.

20. Jarvis T, Scott F, El-Jardali F, Alvarez E. Defining and classifying public health systems: a critical interpretive synthesis. Health Res Policy Syst. 2020 Jun 16;18(1):68.

21. Booth A, Noyes J, Flemming K, Moore G, Tunçalp Ö, Shakibazadeh E. Formulating questions to explore complex interventions within qualitative evidence synthesis. BMJ Glob Health. 2019 Jan 1;4(Suppl 1):e001107.

22. Bryman A. Social research methods. 4th ed. Oxford ; New York: Oxford University Press; 2012. 766 p.

For peer review only - http://bmjopen.bmj.com/site/about/guidelines.xhtml

23. O'Brien KS, Soliman AS, Annan K, Lartey RN, Awuah B, Merajver SD. Traditional herbalists and cancer management in Kumasi, Ghana. J Cancer Educ Off J Am Assoc Cancer Educ. 2012;27(3):573–9.

24. Yamoah K, Beecham K, Hegarty SE, Hyslop T, Showalter T, Yarney J. Early results of prostate cancer radiation therapy: an analysis with emphasis on research strategies to improve treatment delivery and outcomes. BMC Cancer. 2013;13(100967800):23.

25. Binka C, Nyarko SH, Awusabo-Asare K, Doku DT. Barriers to the Uptake of Cervical Cancer Screening and Treatment among Rural Women in Ghana. BioMed Res Int. 2019;2019(101600173):6320938.

26. Asoogo C, Duma SE. Factors contributing to late breast cancer presentation for health care amongst women in Kumasi, Ghana. Curationis. 2015;38(1).

27. Sanuade OA, Ayettey H, Hewlett S, Dedey F, Wu L, Akingbola T, et al. Understanding the causes of breast cancer treatment delays at a teaching hospital in Ghana. J Health Psychol. 2021;26(3):357–66.

28. Yarney J, Donkor A, Opoku SY, Yarney L, Agyeman-Duah I, Abakah AC, et al. Characteristics of users and implications for the use of complementary and alternative medicine in Ghanaian cancer patients undergoing radiotherapy and chemotherapy: a cross- sectional study. BMC Complement Altern Med. 2013;13(101088661):16.

29. Agbokey F, Kudzawu E, Dzodzomenyo M, Ae-Ngibise KA, Owusu-Agyei S, Asante KP. Knowledge and Health Seeking Behaviour of Breast Cancer Patients in Ghana. Int J Breast Cancer. 2019;2019:5239840.

30. Agbeko AE, Arthur J, Bayuo J, Kaburi BB, Kyei I. Seeking healthcare at their 'right' time; the iterative decision process for women with breast cancer. BMC Cancer. 2020 Oct 19;20(1):1011.

31. Martei YM, Vanderpuye V, Jones BA. Fear of Mastectomy Associated with Delayed Breast Cancer Presentation Among Ghanaian Women. The oncologist. 2018;23(12):1446–52.

32. Dedey F, Wu L, Ayettey H, Sanuade OA, Akingbola TS, Hewlett SA, et al. Factors Associated With Waiting Time for Breast Cancer Treatment in a Teaching Hospital in Ghana. Health Educ Behav Off Publ Soc Public Health Educ. 2016;43(4):420–7.

33. Obrist M, Osei-Bonsu E, Awuah B, Watanabe-Galloway S, Merajver SD, Schmid K, et al. Factors related to incomplete treatment of breast cancer in Kumasi, Ghana. Breast Edinb Scotl. 2014;23(6):821–8.

34. Iddrisu M, Aziato L, Ohene LA. Socioeconomic impact of breast cancer on young women in Ghana: A qualitative study. Nurs Open. 2021 Jan;8(1):29–38.

35. Agyemang LS, Foster C, McLean C, Fenlon D, Wagland R. The cultural and structural influences that 'hide' information from women diagnosed with breast cancer in Ghana: an ethnography. BMC Womens Health. 2021 Oct 15;21(1):364.

36. Atobrah D. When darkness falls at mid-day: young patients' perceptions and meanings of chronic illness and their implications for medical care. Ghana Med J. 2012;46(2 Suppl):46–53.

For peer review only

37. Mburu W, BOAMAH MENSAH AB, Virnig B, Amuasi J, Awuah B, Porta C, et al. Pathways to Breast Cancer Diagnosis and Treatment Among Women in Ghana: A Qualitative Study. Womens Health Rep. 2021 Jul 1;2:234–44.

38. Hobenu KA, Naab F. Accessing specialist healthcare: experiences of women diagnosed with advanced cervical cancer in Ghana. Br J Healthc Manag. 2022 Feb 2;28(2):1–11.

39. Asobayire A, Barley R. Women's cultural perceptions and attitudes towards breast cancer: Northern Ghana. Health Promot Int. 2015;30(3):647–57.

40. Nartey Y, Hill PC, Amo-Antwi K, Nyarko KM, Yarney J, Cox B. Characteristics of Women Diagnosed with Invasive CervicalCancer in Ghana. Asian Pac J Cancer Prev APJCP. 2018 Feb 26;19(2):357–63.

41. Scherber S, Soliman AS, Awuah B, Osei-Bonsu E, Adjei E, Abantanga F, et al. Characterizing breast cancer treatment pathways in Kumasi, Ghana from onset of symptoms to final outcome: Outlook towards cancer control. Breast Dis. 2014 Apr 2;34(4):139–49.

42. Asamoah F a., Yarney J, Vanderpuye V, Awasthi S, Venkat P s., Dadzie M a., et al. Definitive Radiation Treatment Patterns and Outcomes for Low Intermediate Risk Prostate Cancer - A Cross Continental Comparative Study between Ghana and the United States of America. Int J Radiat Oncol Biol Phys. 2018 Nov 2;102:e97–e97.

43. Dunyo P, Effah K, Udofia EA. Factors associated with late presentation of cervical cancer cases at a district hospital: a retrospective study. BMC Public Health. 2018 Oct 3;18(1):1156.

44. Brinton L, Figueroa J, Adjei E, Ansong D, Biritwum R, Edusei L, et al. Factors contributing to delays in diagnosis of breast cancers in Ghana, West Africa. Breast Cancer Res Treat. 2017;162(1):105–14.

45. Aziato L, Clegg-Lamptey JNA. Breast cancer diagnosis and factors influencing treatment decisions in Ghana. Health Care Women Int. 2015;36(5):543–57.

46. Twahir M, Oyesegun R, Yarney J, Gachii A, Edusa C, Nwogu C, et al. Real-world challenges for patients with breast cancer in sub-Saharan Africa: a retrospective observational study of access to care in Ghana, Kenya and Nigeria. BMJ Open. 2021 Mar;11(3):e041900.

47. Salifu Y, Almack K, Caswell G. 'My wife is my doctor at home': A qualitative study exploring the challenges of home-based palliative care in a resource-poor setting. Palliat Med. 2021;35(1):97–108.

48. Dadzie MA, Aidoo CA, Vanderpuye V. Vulva cancer in Ghana - Review of a hospital based data. Gynecol Oncol Rep. 2017;20(101652231):108–11.

49. Fowler, F.J. Jr. Improving Survey Questions: Design and Evaluation [Internet]. Thousand Oaks, CA: Sage; 1995 [cited 2021 Sep 30]. Available from: https://us.sagepub.com/en-us/nam/improving-survey-questions/book4994

50. McCutchan G, Weiss B, Quinn-Scoggins H, Dao A, Downs T, Deng Y, et al. Psychosocial influences on help-seeking behaviour for cancer in low-income and lower middle-income countries: a mixed-methods systematic review. BMJ Glob Health. 2021 Feb;6(2).

For peer review only - http://bmjopen.bmj.com/site/about/guidelines.xhtml

51. Bhakuni H, Abimbola S. Epistemic injustice in academic global health. Lancet Glob Health. 2021 Oct 1;9(10):e1465–70.

52. Murphy A, Palafox B, Walli-Attaei M, Powell-Jackson T, Rangarajan S, Alhabib KF, et al. The household economic burden of non-communicable diseases in 18 countries. BMJ Glob Health [Internet]. 2020 Feb 11 [cited 2020 Apr 22];5(2). Available from: https://www.ncbi.nlm.nih.gov/pmc/articles/PMC7042605/

53. Murphy A, McGowan C, McKee M, Suhrcke M, Hanson K. Coping with healthcare costs for chronic illness in low-income and middle-income countries: a systematic literature review. BMJ Glob Health. 2019 Aug 1;4(4):e001475.

54. Pruitt L, Mumuni T, Raikhel E, Ademola A, Ogundiran T, Adenipekun A, et al. Social barriers to diagnosis and treatment of breast cancer in patients presenting at a teaching hospital in Ibadan, Nigeria. Glob Public Health. 2015;10(3):331–44.

55. Elzawawy AM, Elbahaie AM, Dawood SM, Elbahaie HM, Badran A. Delay in Seeking Medical Advice and Late Presentation of Female Breast Cancer Patients in Most of the World. Could We Make Changes? The Experience of 23 Years in Port Said, Egypt. Breast Care. 2008 Mar;3(1):37–41.

56. Powe BD, Finnie R. Cancer Fatalism: The State of the Science. Cancer Nurs. 2003 Dec;26(6):454???467.

57. Freeman HP. Cancer in the socioeconomically disadvantaged. CA Cancer J Clin. 1989;39(5):266–88.

58. Straughan PT. Fatalism reconceptualized: A concept to predict health screening behavior. J Gend Cult Health. 1998;3(2):85–100.

59. Kruk ME, Gage AD, Arsenault C, Jordan K, Leslie HH, Roder-DeWan S, et al. High-quality health systems in the Sustainable Development Goals era: time for a revolution. Lancet Glob Health. 2018 Nov;6(11):e1196–252.

60. Ozawa S, Sripad P. How do you measure trust in the health system? A systematic review of the literature. Soc Sci Med. 2013 Aug 1;91:10–4.

61. Roder-DeWan S, Gage A, Hirschhorn LR, Twum-Danso NAY, Liljestrand J, Asante-Shongwe K, et al. Level of confidence in and endorsement of the health system among internet users in 12 low-income and middle-income countries. BMJ Glob Health. 2020;5(8).

62. Gyasi RM, Asante F, Yeboah JY, Abass K, Mensah CM, Siaw LP. Pulled in or pushed out? Understanding the complexities of motivation for alternative therapies use in Ghana. Int J Qual Stud Health Well-Being. 2016;11:29667.

63. Oystacher T, Blasco D, He E, Huang D, Schear R, McGoldrick D, et al. Understanding stigma as a barrier to accessing cancer treatment in South Africa: implications for public health campaigns. Pan Afr Med J [Internet]. 2018 Jan 24 [cited 2020 Mar 13];29. Available from: https://www.ncbi.nlm.nih.gov/pmc/articles/PMC5987085/

64. Meacham E, Orem J, Nakigudde G, Zujewski JA, Rao D. Exploring stigma as a barrier to cancer service engagement with breast cancer survivors in Kampala, Uganda. Psychooncology. 2016;25(10):1206–11.

For peer review only - http://bmjopen.bmj.com/site/about/guidelines.xhtml

For peer review only

65. Kleinman A. Patients and Healers in the Context of Culture : an exploration of the borderland between anthropology, medicine, and psychiatry [Internet]. Univ of California Press; 1980 [cited 2020 May 19]. Available from: https://www.ucpress.edu/book/9780520045118/patients-and-healers-in-the-context-of-culture

66. Nyaaba GN, Masana L, Aikins A de-Graft, Stronks K, Agyemang C. Lay community perceptions and treatment options for hypertension in rural northern Ghana: a qualitative analysis. BMJ Open. 2018 28;8(11):e023451.

67. van Brakel WH, Cataldo J, Grover S, Kohrt BA, Nyblade L, Stockton M, et al. Out of the silos: identifying cross-cutting features of health-related stigma to advance measurement and intervention. BMC Med [Internet]. 2019 Feb 15 [cited 2020 Mar 13];17. Available from: https://www.ncbi.nlm.nih.gov/pmc/articles/PMC6376667/

68. Stangl AL, Earnshaw VA, Logie CH, van Brakel W, C. Simbayi L, Barré I, et al. The Health Stigma and Discrimination Framework: a global, crosscutting framework to inform research, intervention development, and policy on health-related stigmas. BMC Med [Internet]. 2019 Feb 15 [cited 2020 Mar 13];17. Available from: https://www.ncbi.nlm.nih.gov/pmc/articles/PMC6376797/

69. Pachankis JE, Hatzenbuehler ML, Wang K, Burton CL, Crawford FW, Phelan JC, et al. The Burden of Stigma on Health and Wellbeing: A Taxonomy of Concealment, Course, Disruptiveness, Aesthetics, Origin, and Peril across 93 Stigmas. Pers Soc Psychol Bull. 2018 Apr;44(4):451–74.

70. Turan JM, Elafros MA, Logie CH, Banik S, Turan B, Crockett KB, et al. Challenges and opportunities in examining and addressing intersectional stigma and health. BMC Med. 2019 Feb 15;17(1):7.

71. Kane JC, Elafros MA, Murray SM, Mitchell EMH, Augustinavicius JL, Causevic S, et al. A scoping review of health-related stigma outcomes for high-burden diseases in low- and middle-income countries. BMC Med. 2019 Feb 15;17(1):17.

72. Chase E, Walker R. The Co-construction of Shame in the Context of Poverty: Beyond a Threat to the Social Bond. Sociology. 2013 Aug 1;47(4):739–54.

73. Dake FAA. Examining equity in health insurance coverage: an analysis of Ghana's National Health Insurance Scheme. Int J Equity Health. 2018 Jun 18;17(1):85.

74. Dixon J, Tenkorang EY, Luginaah I. Ghana's National Health Insurance Scheme: Helping the Poor or Leaving Them Behind? Environ Plan C Gov Policy. 2011 Dec 1;29(6):1102–15.

75. Millicent Kotoh A, Aryeetey GC, der Geest SV. Factors That Influence Enrolment and Retention in Ghana' National Health Insurance Scheme. Int J Health Policy Manag. 2017 Oct 17;7(5):443–54.

76. Okoroh J, Essoun S, Seddoh A, Harris H, Weissman JS, Dsane-Selby L, et al. Evaluating the impact of the national health insurance scheme of Ghana on out of pocket expenditures: a systematic review. BMC Health Serv Res. 2018 Jun 7;18(1):426.

77. Ghana NHIS Medicines list.

For peer review only - http://bmjopen.bmj.com/site/about/guidelines.xhtml

78. NHIS Review [Internet]. [cited 2020 Dec 23]. Available from: http://www.nhis.gov.gh/nhisreview.aspx

79. Ministry of Health. NHI Policy Framework for Ghana (Revised Version August 2004) [Internet]. 2004. Available from: https://www.ghanahealthservice.org/downloads/NHI_policy%20framework.pdf

80. Ghana's Minister of Health launches the National HTA Steering Committee and calls for HTA institutionalisation in the country [Internet]. iDSI. 2019 [cited 2019 Nov 12]. Available from: https://www.idsihealth.org/blog/ghanas-minister-of-health-launches-the-national-hta-steering-committee-and-calls-for-hta-institutionalisation-in-the-country/

81. Hollingworth S, Gad M. Health technology assessment case study: Ghana. F1000Research [Internet]. 2018 Jul 2 [cited 2019 Nov 12];7. Available from: https://f1000research.com/slides/7-979

82. Amegbor PM, Kuuire VZ, Bisung E, Braimah JA. Modern or traditional health care? Understanding the role of insurance in health-seeking behaviours among older Ghanaians. Prim Health Care Res Dev. 2019;1:e71.

83. Appiah B, Amponsah IK, Poudyal A, Mensah MLK. Identifying strengths and weaknesses of the integration of biomedical and herbal medicine units in Ghana using the WHO Health Systems Framework: a qualitative study. BMC Complement Altern Med [Internet]. 2018 Oct 22 [cited 2020 Mar 27];18. Available from: https://www.ncbi.nlm.nih.gov/pmc/articles/PMC6196414/

84. Ghana Ministry of Health. Traditional Medicines Policy. 2005;15.

85. Dako-Gyeke P, Aikins M, Aryeetey R, McCough L, Adongo PB. The influence of socio-cultural interpretations of pregnancy threats on health-seeking behavior among pregnant women in urban Accra, Ghana. BMC Pregnancy Childbirth. 2013 Nov 19;13:211.

86. WHO, Africa CDC in joint push for COVID-19 traditional medicine research in Africa [Internet]. Africa CDC. [cited 2021 Sep 28]. Available from: https://africacdc.org/news-item/who-africa-cdc-in-joint-push-for-covid-19-traditional-medicine-research-in-africa/

87. WHO establishes the Global Centre for Traditional Medicine in India [Internet]. [cited 2022 Apr 1]. Available from: https://www.who.int/news/item/25-03-2022-who-establishes-the-global-centre-for-traditional-medicine-in-india

88. Barry CA. The role of evidence in alternative medicine: Contrasting biomedical and anthropological approaches. Soc Sci Med. 2006 Jun 1;62(11):2646–57.

89. Baer HA. Toward an Integrative Medicine: Merging Alternative Therapies with Biomedicine. Walnut Creek, CA: Altamira Press; 2004. 224 p.

For peer review only

For peer review only - http://bmjopen.bmj.com/site/about/guidelines.xhtml

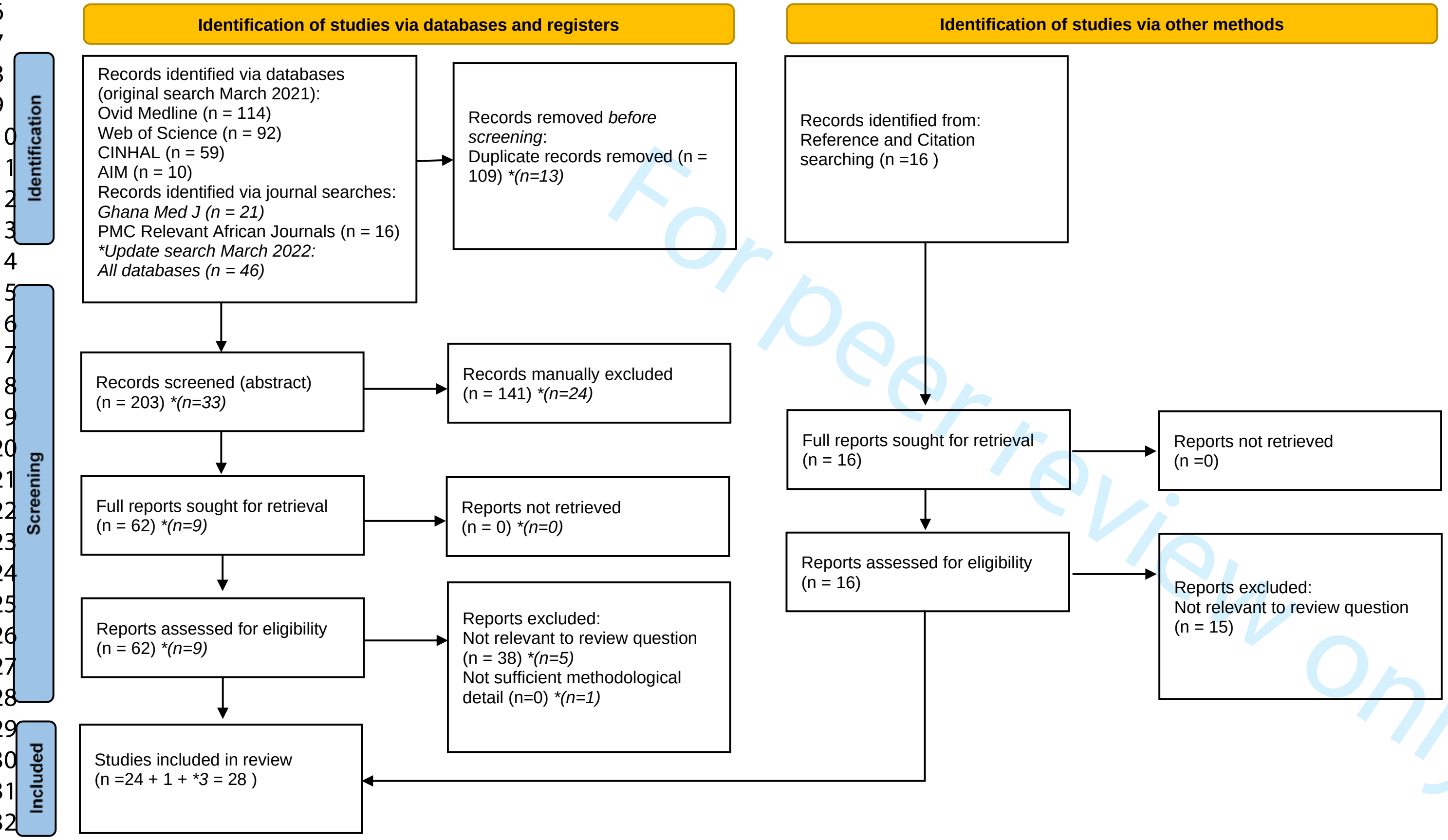

For peer review only

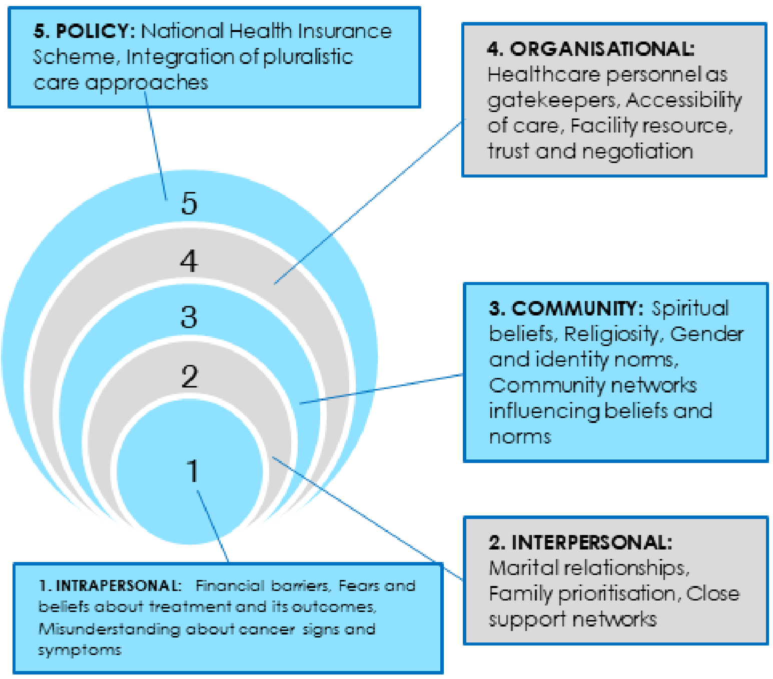

For peer review only

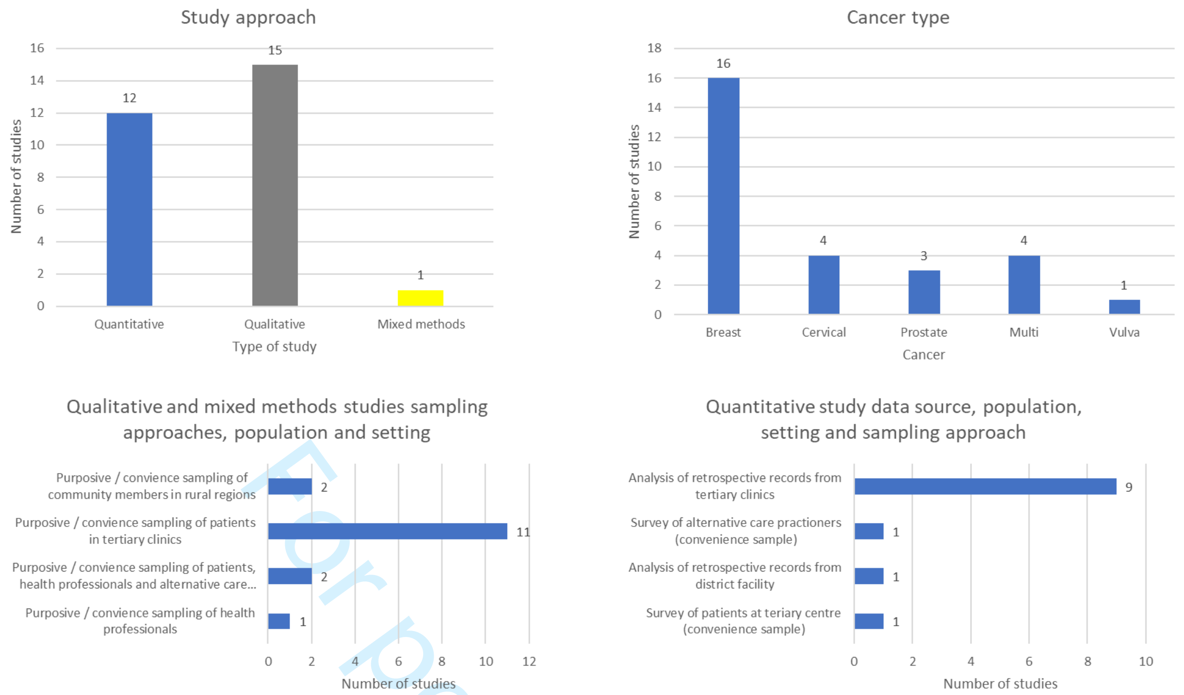

For peer review only

Supplementary information: Evidence synthesis tables

Supplementary Table 1: Summary of included studies

Author and year	Setting (region)	Study approach	Population and sample	Methods (design, sampling, data analysis)	Phenomenon	Findings	Limitations noted	Candidacy stage
Binka et al., 2019	Rural community setting and catholic hospital; North Tongu District (Volta region)	Qualitative (deductive)	Sample 1: 15 cervical cancer patients<br>Sample 2: 40 women aged between 30 and 65 registered at the hospital but not screened	Semi-structured interviews and focus group, convenience sampling, deductive thematic analysis	Cervical cancer screening and treatment uptake	- financial burden of treatment due to a lack of government subsidies for those with low household income<br>- expected provision of financial support with husbands' role<br>- Spiritual beliefs about the cause of disease and in the efficacy of traditional medicine led to spiritual and traditional alternatives being used first.<br>Possible institutional-level barriers (privacy, health worker attitudes, potential misdiagnosis) and unavailability of screening and treatment facilities in rural areas	-	Presentation, negotiation, acceptance
Aziato et al., 2015	Tertiary clinic, Accra Region	Qualitative (exploratory descriptive)	12 Ghanaian women who have undergone a mastectomy (speaking English, Twi, or Ewe)	Semi-structured interviews, purposive sampling, thematic analysis	Breast cancer treatment intentions	Treatment decisions are based on knowledge and perceptions and influenced by husbands and family; without their financial and emotion support may led to delays<br>Patients believed that alternative treatment could treat breast cancer, and sought them after diagnosis, which was influenced by husbands' views<br>Patients perceived health systems reason for treatment delays were:<br>- incorrect diagnosis at district level<br>- waiting for biopsy results	-	Negotiation, acceptance
Asoogo et al., 2015	Tertiary clinic, Ashanti Region	Qualitative descriptive	30 women diagnosed with breast cancer who presented with Stage II and Stage III	semi-structured interviews, convenience sampling, content data analysis	Breast cancer presentation	- financial barriers, including leading to alternative use<br>- misinformation, considering abnormalities normal development<br>- fear of treatment and outcomes influenced by supports and others in their community's experiences<br>- prioritising care of other<br>- belief in the efficacy of alternative medicines led to seeking first, influenced by affordability<br>- fear of losing female identity and capacity for motherhood	-	Presentation

For peer review only

Martei et al., 2018	Tertiary clinic, Accra Region	Qualitative (inductive)	31 women with breast cancer	Semi structured interviews, purposive sampling, grounded theory	Breast cancer presentation	- financial barriers including prioritisation costs of family and frustration on poor coverage by NHIS<br>- negative husband views<br>- loss of gender identity<br>- misinformation, considering abnormalities harmless, misconceptions that treatment was deadly<br>The church played a supportive role for presentation.	Bias in sample representation (age, religion Christian)<br>Sampling bias	Presentation, acceptance
Agbokey et al., 2019	Tertiary clinic, Ashanti Region	Qualitative descriptive/exploratory	20 patients / 8 caregivers with breast cancer<br>5 health workers<br>2 herbalists	Semi structured in-depth interviews, purposive sampling, thematic analysis	Breast cancer health seeking behaviours	- financial barriers, including prioritising family costs and frustration at poor coverage by NHIS<br>- family support to secure funds<br>- lack of information<br>- beliefs influenced by husbands and family<br>- husbands role in financial decisions<br>- seeking alternative medicines, influenced by affordability and family and friends	Clinic setting may cause participants reluctance to speak openly	Presentation, negotiation, acceptance
Salifu et al., 2021	Tertiary clinic, urban region	Qualitative at 2 time points	Men with advanced prostate cancer (n = 23), family caregivers (n = 23), healthcare professionals (n = 12).	Semi structured interviews at 2 time points, purposive sampling, thematic analysis, Social constructivist theory and interpretivism	Home based palliative care for prostate cancer	- selection of alternative medicines influenced by challenges in accessing medicines and perceived poor supervision by health professionals	Limited sample location<br>Researcher interpretation bias	Acceptance
Bonsu et al., 2019	Tertiary clinic, Ashanti Region	Qualitative descriptive/exploratory	11 patients with breast cancer diagnosed at stage II/IV	Semi-structured interviews, purposive sampling, thematic analysis<br>Applying adapted model of health seeking behaviour (Andersen model of total patient delay)	Breast cancer presentation	- poor symptom recognition, considering changes to breasts normal<br>- lack of knowledge on where to seek support<br>-fears and fatalistic perceptions<br>- husbands views<br>- prioritisation of family and work obligations<br>- Misrecognition by health professionals*<br>- Lack of trust in health sector due to perceived mismanagement<br>- spiritual and community held beliefs about cancer led to alternative use (acting at all stages)*<br>- religious leaders supported earlier presentation	Sample site bias<br>Recall bias	Presentation (predominantly - exceptions*)

For peer review only

						- some religious messages caused delays<br>- influence of trusted social network (husband, family, religious leaders, health workers in their community, colleagues)		
Agbeko et al., 2020	Tertiary clinic, Ashanti Region	Qualitative descriptive (deductive)	15 women presenting with stage III/IV breast cancer	Semi-structured interviews, purposively sampling, deductive thematic analysis with priori themes (Andersen Behavioural Model of Health care utilisation)	Breast cancer presentation	- financial barriers to referral process<br>- poor symptom recognition, not regarded as serious, influenced by community beliefs<br>- prioritisation of family roles and finances, influenced by gender identity and roles beliefs<br>- referral delays when first contact not a specialist	Recall bias<br>Unable to follow-up participants for respondent validation<br>Researcher and interviewer bias	Presentation, negotiation, acceptance
Sanuade et al., 2021	Tertiary clinic, Accra Region	Qualitative descriptive (deductive and inductive - exploratory)	20 women who have commenced treatment	4 focus group discussions, purposive sampling following survey, deductive and inductive thematic analysis	Breast cancer treatment delays	- high cost of treatment regardless of social economic status (leading using alternatives)<br>- fear of losing female identity<br>- fear of treatment centre trauma (lack of institution trust)<br>- husbands opinion<br>- impolite treatment by health professionals and perceived corruption<br>- health professionals advising alternative treatments<br>- patients perceived health facility delays including waiting for biopsy results, medicines and workforce shortages, machines breaking<br>- spiritual beliefs led to seeking alternatives, influenced by cost and others (religious leaders, health workers)<br>- community network opinions	Low number of participants	Negotiation and acceptance
Atobrah et al., 2012	Tertiary clinic, Accra Region	Qualitative (descriptive)	9 young Ga adults (25-35) with chronic diseases; cancer of the breast, cancer of the ovaries, cancer of the nasopharynx, cancer of the cervix, stroke and chronic renal failure	Multiple narrative interviews and observations (12 each) Purposive sampling (on socio-economic status) Thematic analysis using symbolic interactionism and grief frameworks	Early onset of chronic diseases	- poor symptom recognition given young age<br>- advise to seek alternative therapies from friends/family<br>- misdiagnosis<br>- community beliefs (including of spiritual causes) led to seeking alternative treatments<br>- social networks and community members influenced beliefs and choices	-	Presentation, negotiation, acceptance

For peer review only

Asobayire et al., 2015	(District hospital, unclear) Kassena-Nankana (Navrongo, Upper East Region)	Qualitative (descriptive, inductive)	10 community women (six farmers, two traders and two teachers - aged 25 -56)	Focus group interviews and documentary analysis of current practices, purposive sampling (not stated) for 10 settlements and differing community and socioeconomic status, thematic analysis following an inductive analytical framework	Breast cancer perceptions	- local dialect disease name translation causes misunderstanding<br>- husbands and community leader roles in decision making<br>- community beliefs about the efficacy of traditional medicines and spiritual beliefs lead to alternatives being sought first<br>- potential for positive influence of religious and community leaders and female groups, if engaged	-	Presentation (predominantly)
Iddrisu et al., 2021	Three hospitals in Accra (regional, military and university)	Qualitative (descriptive exploratory)	12 young patients (15-49 year) with breast cancer	Semi structured interviews, purposive and snowball sampling, thematic analysis	Breast cancer socio economic impact	- financial barriers to treatment (including as NHIS did not cover costs)<br>- Negative misbeliefs about treatment, influenced by friends, family and community gossip<br>- spiritual beliefs held in the community led to seeking alternatives, influenced by friends and family	-	Acceptance
Ayandipo et al., 2020	With 3 country analysis: 3 tertiary clinics, four regional, four district facilities (1 private)	Mixed methods cross sectional study	Stakeholders in NCD programme, MoH and CSOs Heads of the selected facilities or units and cancer specialists (120 health workers in Ghana, 10% oncology qualified)	Standardised questionnaire, desk based literature review, Thematic analysis Gap analysis using WHO framework for health system strengthening with the 6 pillars	Gaps in cancer control	- patient unaffordability of treatment due to lack of coverage on the NHIS<br>- neglect by families<br>- misdiagnosis, improper documentation and staff shortages, particularly outside of major tertiary centres<br>- shortages of medicines, commodities, and machine breakdown<br>- patient preference for traditional medicines	-	Negotiation and acceptance
Nartey et al., 2018	Tertiary clinics in Accra and Ashanti region	Quantitative (retrospective observational)	1725 confirmed invasive cervical cancer cases	Retrospective review of patient record, hospital population sampling, $\chi2$ and logistic (multivariate)regression of factors influencing stage of diagnosis	Cervical cancer stage at diagnosis	- treatment non acceptance for financial reasons<br>- marital status (widowed, divorced, other), ethnicity and (increased) age associated with late diagnosis	Inconsistency, errors and missing data in record keeping<br>Difficulties following up patients/family	Presentation (predominantly)

For peer review only

Scherber et al., 2014	Tertiary clinic in Ashanti region	Quantitative (retrospective observational)	597 breast cancer patients	Review of patient records hospital population sampling, uni and multivariate logistic regression of factors influencing complete treatment follow up	Breast cancer incomplete follow up	- weak positive trend between age and stage diagnosis<br>- no differences in presentation stage whether attended clinic directly or referred from elsewhere<br>- 12.2% patients sought alternative therapies although this does not explain high rates of treatment interruptions/incompletion (73.1% loss to follow up)<br>- presence of hormone status report associated with complete treatment follow up, inferred to reflect the capacity to pay for tests and thus treatment	Inconsistencies with record keeping and missing data Reported barriers only known from those who return for treatment (those who report barriers were more likely to complete treatment)	Acceptance (predominantly, also presentation)
Asamoah et al., 2018	Tertiary clinic in Accra region	Quantitative (retrospective cohort)	1,074 patients with prostate cancer	Retrospective review of records, hospital population sampling, descriptive statistics ($\chi 2$ and Fisher's exact tests, Mann-Whitney U tests) and survival analysis	Prostate cancer treatment	- higher median age for presenting with advanced disease	Missing data<br>Hospital records excluded those not able to access	Presentation
Yamoah et al., 2013	Tertiary clinic in Accra region	Quantitative (retrospective cohort)	379 patients with prostate cancer	Retrospective review of patient history, hospital population sampling, descriptive statistics, non-parametric significance tests (due to data skew), survival analysis	Prostate cancer treatment	- treatment non acceptance due to direct and indirect costs being unaffordable, fear of radiation therapy and spiritual beliefs<br>- age was not significantly associated with diagnosis stage	-	Acceptance
Dedey et al., 2016	Tertiary clinic in Accra region	Quantitative (retrospective observational)	205 treated breast cancer patients	Retrospective review of records and patient questionnaire, all patients treated (May - Dec 2013), descriptive statistics, non-parametric tests, Poisson multivariate regression on wait time	Breast cancer waiting time	In a multivariate Poisson regression wait time increased with:<br>- low-income low education, low income, Akan ethnicity, aged 50 or over, marital status (single) but not religion<br>- biopsy time<br>- being insured on NHIS (inferred to be due to delays in recouping funds)<br>- Perceived receipt of adequate information, other perceptions of health workers and service were not significant	Sample representation bias<br>Recall bias	Negotiation

For peer review only

Brinton et al., 2017	Three tertiary clinics in Accra and Ashanti region	Quantitative (retrospective observational)	1,184 patients with malignant breast cancer	Retrospective review of case control study data through structured patient interview, factors influencing tumour mass assessed in multivariate parametric model	Breast cancer diagnosis	In a multivariate model, larger mass at diagnosis correlated with:<br>- low education<br>- marital status (widowed/divorced)<br>- seeking assistance from someone other than a doctor or nurse<br>- use of traditional medicines<br>travel and financial difficulties in seeking care were not significant	Limited sampling catchment may limit possible impact of treatment distance	Presentation
Obrist et al., 2014	Tertiary clinic in Ashanti region	Quantitative (Retrospective case control)	141 women/next of kin with breast cancer	Retrospective review of medical records and semi structured interviews, purposive sampling, statistical analysis by parametric t-tests and non-parametric univariate analysis, multivariate model accounting for confounders using logistic regression	Breast cancer treatment incompletion	- non completing patients were more likely to believe they will not respond, but this was not significant in the LR model<br>- practicing Islam was a significantly higher in non-completing group<br>- age was not significant<br>- explored health systems factors were not significant predictors of completion<br>- seeing a traditional healer after any visit was a significant predictor of not completing after the combined analysis<br>- more non completers believed a traditional healer was better at managing cancer (significant at 10% level)<br>- Understanding what the NHIS cover for breast cancer included was significant predictor of completing treatment	Missing contact information and patient follow up<br>Recall bias by next of kin (more likely in DNC group)	Acceptance
Twahir et al., 2021	Two tertiary clinics in Accra region (as part of a study across Ghana, Kenya and Nigeria)	Quantitative (retrospective observational)	299 breast cancer patients undergoing treatment	Retrospective review of patient records in standard collection framework, hospital population sampling, descriptive statistical analysis	Breast cancer access to care	Paying out of pocket costs was associated with receiving more treatment cycles	-	Negotiation and acceptance

For peer review only

O'Brien et al., 2011	Community setting, Ashanti region	Quantitative (descriptive)	42 Traditional Medicine Practice Council (TMPC) registered traditional practitioner	Semi-structured interviews, convenience sampling, descriptive analysis	Traditional herbalists' role in cancer management	Traditional herbalists perceive patient barriers to access include:<br>- cost<br>- lack of knowledge of cancer and fear of treatment<br>- distance and insufficient numbers of health centres<br>- stigma<br>Traditional herbalists poor knowledge of cancer and treatment, and reluctance to refer to other services prevent patients receiving timely care<br>This is influenced by other health professionals' reluctance to collaborate and lack of integration into the health system	-	Presentation, negotiation, acceptance (not specified)
Dadzie et al., 2017	Tertiary clinic in Accra region	Quantitative (retrospective observational)	70 vulva cancer cases	Retrospective review all hospital cases, hospital population sampling, descriptive statistics	Vulva cancer treatment	Cost influenced patient choice (other access barriers were inferred)	-	Acceptance (other access barriers were inferred)
Dunyo et al., 2018	Oncology clinic, Christian hospital (Battor), Volta Region	Quantitative (retrospective observational – cross-sectional analytical)	157 cervical cancer patients	Retrospective review all hospital cases, hospital population sampling, descriptive statistics, non-parametric significance tests, logistic regression of influences on late presentation	Cervical cancer presentation	Education (low) was a significant predictor of late presentation<br>Age was not significant	Missing data, retrospective nature prevented follow up	Presentation
Yarney et al., 2013	Tertiary clinic in Accra region	Quantitative (cross-sectional descriptive)	98 cancer patients	Questionnaire, convenience sampling, descriptive analysis	CAM use in cancer patients	Comparatively high cost of conventional treatment meant only CAM treatment was feasible<br>Lower age, decreased education and being married made it more likely to use CAM in logistic regression model, CAM use influenced by friends and family	Non probabilistic sampling<br>Single institution sample<br>Low response rate<br>Sample bias: excluded those who use CAM but not attending hospital	Acceptance
Mburu 2021	Tertiary clinic, Ashanti Region	Qualitative (empircal phenomenological approach)	31 women with breast cancer	Purposive sampling, semi-structured interviews, deductive coding approach	Breast cancer pathway	Misinterpretation of symptoms<br>Initial symptom management with OTC medicines or waiting to see if it improves<br>Women disclosed to friends and relatives, most advised to go to the hospital, but some advised to wait and monitor or see a traditional healer	Selection bias (not able to include those who did not attend hospital)<br>Remembering of participants and recall bias	Access, negotiation and acceptance

For peer review only

						Misdiagnosis and Complex multiple stage referral process led to delays<br>Initial and later stage management through traditional and faith-based healing		
Hobenu and Naab 2022	Teriary clinic, Accra Region	Qualitative (exploratory)	15 Women with cervical cancer	Purposive sampling, in depth interviews, thematic analysis	Cervical cancer from diagnosis to accessing treatment	Misinterpretation of symptoms led to delays in identifying at services<br>Husbands, relatives and friends advised to use herbal treatments<br>Misdiagnosis and Delays due to waiting a long time to get results, having to go to many hospitals to get diagnosed and rescheduling of appointments	Small sample and purposive sampling leading to selection bias	Access and negotiation
Agyemang 2021	Tertiary clinic, Ashanti Region	Qualitative (ethnographic approach)	31 breast cancer patients (up to stage 3), relatives, nurses and doctors observed and 29 took part in interviews	Purposive (max variation) sampling, 2 time point observations, interviews, thematic analysis	Breast cancer formal diagnosis to treatment	Deliberate miscommunication to stop drop out<br>Lack of honest discussion about fertility concerns meant patients fears were not addressed and consequently led to loss to follow up<br>Lack of support around financial issues<br>Costs were barrier to adherence in HCP opinion	Selection bias (not able to include those who did not attend hospital)<br>Small sample	Acceptance

Supplementary Table 2: Evidence map using socio ecological model and candidacy as a framework of access to care

Author and year	Design	Location	Setting	Cancer	Intrapersonal	Interpersonal	Institutional	Community	Policy
Agbeko et al., 2020	Qualitative	Kumasi	Clinic	Breast	A, B	A, C	A, B	A	
Agbokey et al., 2019	Qualitative	Kumasi	Clinic	Breast	A, B, C	A, C	B, C	A	
Agyemang 2021	Qualitative	Kumasi	Clinic	Breast			C		C
Asamoah et al., 2018	Quantitative	Accra	Clinic	Prostate	A				
Asobayire et al., 2015	Qualitative	Upper East	Community	Breast	A	A	B	A	
Asoogo et al., 2015	Qualitative	Kumasi	Clinic	Breast	A	A, B		A	
Atobrah et al., 2012	Qualitative	Accra	Clinic	Multi	A, C	A	B	A, C	
Ayandipo et al., 2020	Mixed methods	Multi-site	Multi-site (health facility)	Multi	B, C	B, C	B, C		C
Aziato et al., 2015	Qualitative	Accra	Clinic	Breast	C	C	B	C	
Binka et al., 2019	Qualitative	Volta	Community	Cervical	A	A, C	A, B, C	A	A, C
Bonsu et al., 2019	Qualitative	Kumasi	Clinic	Breast	A, C	A	A, B, C	A, B, C	
Brinton et al., 2017	Quantitative	Accra / Kumasi	Clinic	Breast	A	A	A	A	

Dadzie et al., 2017	Quantitative	Accra	Clinic	Vulva	C		C		
Dedey et al., 2016	Quantitative	Accra	Clinic	Breast	B		B		
Dunyo et al., 2018	Quantitative	Volta	Clinic	Cervical	A				
Hobenu and Naab 2021	Qualitative	Accra	Clinic	Cervical	A	A	B		
Iddrisu et al., 2021	Qualitative	Accra	Clinic	Breast	C	A	C	C	C
Martei et al., 2018	Qualitative	Accra	Clinic	Breast	A, C	A, C		A, C	C
Mburu 2021	Qualitative	Kumasi	Clinic	Breast	A	A	B	A,C	
Nartey et al., 2018	Quantitative	Accra / Kumasi	Clinic	Cervical	A	A		A	
O'Brien et al., 2011	Quantitative	Kumasi	Community	Multi	A, B, C		B, C	C	C
Obrist et al., 2014	Quantitative	Kumasi	Clinic	Breast	C	C	C	C	C
Salifu et al., 2021	Qualitative	Kumasi	Clinic	Prostate			C	C	
Sanuade et al., 2021	Qualitative	Accra	Clinic	Breast	C	C	B, C	C	
Scherber et al., 2014	Quantitative	Kumasi	Clinic	Breast	A, C		A, C	C	
Twahir et al., 2021	Quantitative	Accra	Clinic	Breast			B		C

Yamoah et al., 2013	Quantitative	Accra	Clinic	Prostate	A, C				
Yarney et al., 2013	Quantitative	Accra	Clinic	Multi	C		C	C	

Key: A= candidacy stage ACCESS, B= candidacy stage NEGOTIATION, C= candidacy stage ACCEPTANCE

For peer review only

BMJ Open

What influences cancer treatment service access in Ghana? A critical interpretive synthesis

Journal:	*BMJ Open*
Manuscript ID	bmjopen-2022-065153.R1
Article Type:	Original research
Date Submitted by the Author:	17-Aug-2022

Complete List of Authors:	Tuck, Chloe; The University of Sheffield, School of Health and Related Research (ScHARR)<br>Akparibo, Robert; The University of Sheffield, School of Health and Related Research (ScHARR)<br>Gray, Laura; The University of Sheffield, School of Health and Related Research (SCHARR)<br>Aryeetey, Richmond; University of Ghana, Population Family and Reproductive Health<br>Cooper, Richard; The University of Sheffield, School of Health and Related Research (ScHARR)

**Primary Subject Heading**:	Global health
Secondary Subject Heading:	Oncology, Health policy, Health services research
Keywords:	Health economics < HEALTH SERVICES ADMINISTRATION & MANAGEMENT, PUBLIC HEALTH, Adult oncology < ONCOLOGY, Health policy < HEALTH SERVICES ADMINISTRATION & MANAGEMENT

SCHOLARONE™
Manuscripts

I, the Submitting Author has the right to grant and does grant on behalf of all authors of the Work (as defined in the below author licence), an exclusive licence and/or a non-exclusive licence for contributions from authors who are: i) UK Crown employees; ii) where BMJ has agreed a CC-BY licence shall apply, and/or iii) in accordance with the terms applicable for US Federal Government officers or employees acting as part of their official duties; on a worldwide, perpetual, irrevocable, royalty-free basis to BMJ Publishing Group Ltd ("BMJ") its licensees and where the relevant Journal is co-owned by BMJ to the co-owners of the Journal, to publish the Work in this journal and any other BMJ products and to exploit all rights, as set out in our licence.

The Submitting Author accepts and understands that any supply made under these terms is made by BMJ to the Submitting Author unless you are acting as an employee on behalf of your employer or a postgraduate student of an affiliated institution which is paying any applicable article publishing charge ("APC") for Open Access articles. Where the Submitting Author wishes to make the Work available on an Open Access basis (and intends to pay the relevant APC), the terms of reuse of such Open Access shall be governed by a Creative Commons licence – details of these licences and which Creative Commons licence will apply to this Work are set out in our licence referred to above.

Other than as permitted in any relevant BMJ Author's Self Archiving Policies, I confirm this Work has not been accepted for publication elsewhere, is not being considered for publication elsewhere and does not duplicate material already published. I confirm all authors consent to publication of this Work and authorise the granting of this licence.

For peer review only

What influences cancer treatment service access in Ghana? A critical interpretive synthesis

Chloe Tuck[1], Robert Akparibo[1], Laura Gray[1], Richmond Aryeetey[2], and Richard Cooper[1]

1. School of Health and Related Research, University of Sheffield, Sheffield, UK
2. School of Public Health, University of Ghana, Legon, Accra, Ghana

Corresponding author:

Chloe Tuck, School of Health and Related Research, University of Sheffield, 30 Regent Street, Sheffield, S1 4DA, UK

Chloeztuck@gmail.com

Cztuck1@sheffield.ac.uk

Phone: +447402110248

Word count: 5082

Keywords: critical interpretive synthesis, Ghana, access to cancer treatment, socio-ecological model, health policy

For peer review only

ABSTRACT

Objectives: Multiple social-cultural and contextual factors influence access to and acceptance of cancer treatment in Ghana. The aim of this research was to assess existing literature on how these factors interplay and could be susceptible to local and national policy changes.

Design: This study uses a critical interpretive synthesis approach to review qualitative and quantitative evidence about access to adult cancer treatment services in Ghana, applying the socio-ecological model and candidacy framework.

Results: Our findings highlighted barriers to accessing cancer services within each level of the socio-ecological model (intrapersonal, interpersonal community, organisational and policy levels), that are dynamic and interacting, e.g, community level factors influenced individual perceptions and how they managed financial barriers. Evidence was lacking in relation to determinants of treatment non-acceptance across all cancers and in the most vulnerable societal groups due to methodological limitations.

Conclusions: Future policy should prioritise multi-level approaches, for example improving the quality and affordability of medical care while also providing collaboration with traditional and complementary care systems to refer patients. Research should seek to overcome methodological limitations to understand the determinants of accessing treatment in the most vulnerable populations.

Strengths and limitations of this study

- A strength of this study is the combination of purposive and systematic searches, and the reflexive approach to developing the search strategy which enabled it to cover a wide range of articles.
- Additionally, the critical interpretive synthesis involved a critique of the literature to identify methodological limitations and research gaps.
- However, as only published academic articles are included in this study, it may overlook other forms of evidence, including locally generated and day-to-day working understandings.
- The interpretation of the evidence will reflect the inherent biases in world view of the lead author.

KEY QUESTIONS

What is already known on this topic

Multiple social-cultural and contextual factors influence access to and acceptance of cancer treatment in Ghana. How these factors interplay and could be susceptible to policy changes is unknown.

What this study adds

This study highlights barriers to accessing cancer services within each level of the socio-ecological model that are dynamic and interacting. There is a gap in understanding determinants of treatment non-acceptance across all cancers and in the most vulnerable societal groups.

How this study might affect research, practice or policy

For peer review only - http://bmjopen.bmj.com/site/about/guidelines.xhtml

Findings of this review have potential to impact future policy formulation, which should prioritise multi-level approaches to improve the quality and affordability of medical care whilst harmonising linkages with traditional and complementary care systems.

INTRODUCTION

Cancer is a growing burden in low and middle income countries (LMICs)(1,2). Despite efforts by the World Health Organisation (WHO) to prioritise tackling cancer inequity, hurdles remain due to the limited evidence to inform cost-effective decision-making and the high expense of cancer control(1). In Ghana, cancer treatment is focused in large referral centres in major cities, with disparity in resources and health worker expertise in rural areas, and limited coverage of the National Health Insurance Scheme (NHIS)(3–7). Policy efforts to expand cancer services are further hindered when patients prioritise traditional alternatives over orthodox cancer services(8). Multiple social-cultural, economic and health system factors can influence how patients access, navigate, and choose suitable cancer care in Ghana(3). In addressing this, there is a relative lack of public health surveillance data. There have been recent attempts to reconcile this(9), but a comprehensive understanding remains elusive. One alternative approach is to consider the relevance of theory.

Socio-economic model and candidacy framework perspectives on cancer treatment access

One important way of understanding the complexity and various factors previous described in relation to cancer treatment service access is to consider this in terms of the socio-ecological model (10). This has been used in many settings to map barriers to healthcare engagement from a systems perspective (11,12). The socio-ecological model considers the individual within an ecosystem of intrapersonal, interpersonal, community, health organisational, and policy influences. This has been applied extensively to map systems factors since it was developed by Bronfenbrenner (10), including health behaviours in several African settings (11,12). The process of an individual accessing cancer services is dynamic and delays in access can occur at multiple stages. Patients may experience barriers presenting at services, negotiating the care pathway, being offered and accepting treatment. A Critical interpretive synthesis (CIS) exploring health access in the UK (13) highlighted that this involves dynamic interactions between the individual, the health environment and health professionals. The term 'access' often overlooks this dynamism, whilst terms such as 'uptake' provide a narrow view that overlooks patient demand and service navigation afterwards.

The candidacy framework provides a multi-stage interacting process of patient access holistically. For example, negotiation of services is overlooked in asylum seekers and refugees(14). Van der Boor and colleagues describe candidacy in two broad stages 'access' (identification of eligibility, navigation of services) and 'negotiation' (permeation of services, appearance, adjudications, offers and resistance, and dynamic interactions with local services)(14). Candidacy has been applied to understand patient-doctor interactions influencing cancer health seeking behaviours(15) and in an African context(16).

This paper builds on the issue of factors relating to cancer treatment service access in LMIC settings such as Ghana by presenting the findings of an evidence review that was informed by theory. The primary aim was to systematically review and critique literature from a systems perspective to understand factors influencing cancer treatment service access in Ghana. A further aim was to assess the strengths and limitations of methods associated with existing research relating to this topic.

For peer review only - http://bmjopen.bmj.com/site/about/guidelines.xhtml

METHODS

Using the RETREAT (Review question, Epistemology, Timescale, Resources, Expertise, Audience/purpose, Type of data) framework(17), critical interpretive synthesis (CIS) was considered to be the most suitable approach (13). CIS has been used in a variety of policy and health service settings(18–20) and combines systematic and purposive approaches to identify multiple types of evidence and identify themes following an evidence critique. This involves considering how the problem has been constructed, underlying assumptions and epistemology, and how this has influenced the methodology and conclusions (13).

Search strategy and literature search

The search strategy to find articles on access of adult cancer treatment in Ghana was developed using the question framework PerSPECTiF (Perspective, Setting, Phenomenon of interest, Environment (optional Comparison) Timing, Findings) (21) in consultation with an information specialist. Firstly, primary systematic searches were undertaken. This was tested and refined through pilot searches, before conducting comprehensive searches in Medline (via OVID), Web of Science, CINAHL and African Index Medicus. These databases were chosen following University of Sheffield librarian advice, and after the initial database scoping exercise in Medline (via OVID) and Google Scholar. The database search strategy and terms used can be found in Supplementary Table 1. Initial searches were conducted on 26/03/2021 and the databases were searched for updates on 29/03/2022. Search terms were composed of multiple equivalent thesaurus terms and phrases to cover three elements: Ghana, health service access/uptake of services, and cancer. Hand searches were performed using citation follow-up, identified relevant individual journals, and in the reference listed of included papers.

Study selection

The lead author (CT) screened all titles and abstracts using an agreed inclusion criteria whilst two other authors (RA, RC) conducted quality checks of 10% of the sample screened. Any disagreement was discussed and settled among authors. Initial screening highlighted ambiguity in the screening criteria, which was further refined to ensure consistency prior to formal selection. Inclusion criteria included only primary research conducted with a 10-year time frame to align with Ghana's increased policy interest in efficiently expand national health insurance packages (6). Initial screening highlighted the need for a focused exclusion criteria which was again informed by the question framework PerSPECTiF (21). Applying the PerSPECTiF framework , the phenomenon of interest ('access') was defined holistically through the candidacy framework (13). Thus, article screening sought to include articles relating to access throughout the entire patient pathway. The setting included all levels of the socio-ecological model to provide a systems perspective. Potentially qualifying abstracts were read in full, and only the full texts papers that meet the review inclusion criteria were included and reviewed.

Data extraction and synthesis

Data was extracted from included papers to facilitate decision making and an audit trail (See data extraction in Supplementary Material). The lead author (CT) used a standardised data collection form to extract data from the included studies. To eliminate data extraction bias, two reviewers (RA and RC) two other authors (RA, RC) checked 10% of the extraction. There was no discrepancy observed between the lead author extraction and the sample reviewed. Key data extracted were setting, approach, population and sample, methods design, sampling, data analysis, cancer and stage studied, and the corresponding texts cross-tabulated against the socio-ecological model (10) and the candidacy

For peer review only - http://bmjopen.bmj.com/site/about/guidelines.xhtml

framework. Data was collected as line of arguments (13). First order constructs (taken directly from the data in articles) and second order constructs (author reports from articles) were extracted and separately noted within the framework. It was noted where study authors made further (secondary) inferences and assumptions from data that were not primary findings, but relevant to the themes. Researchers' limitations were recorded. The data were segregated into qualitative, quantitative, and mixed methods studies and each interpreted qualitatively. A synthesising argument(13) was applied to map first and second order findings across studies to interpret the evidence and create new concepts that draw on the whole body of evidence (third order/synthetic constructs). Inferences were mapped across the studies to enable the body of evidence to be critiqued by research question construction, methods, and conclusions and how these fit into the general findings, to identify trends in the literature and limitations with current approaches. To bring together themes in candidacy, the candidacy framework was summarised into three main stages. Van der Boor use two stages(14), however, as themes were identified, it was noted that treatment acceptance and the interactions around this over time play a pertinent role in the patient pathway in Ghana. Thus, the adapted three stage model also notes the importance of the dynamics of treatment acceptance. As part of the synthesis process, the primary literature from the data extraction were revisited and reinterpreted with emerging evidence to ensure critical details or limitations were not missed.

Critical appraisal

A streamline critical appraisal for major and methodological flaws was conducted using the critical appraisal checklist published in Dixon Woods et al.,(13). The quality of quantitative and qualitative findings was assessed in terms of reliability and trustworthiness (22). In line with the CIS, articles were not favoured based on quality alone but contribution of rich insights. The CIS deals with weak evidence through including a critique of methods and approach. Lead author (CT) appraised all the included papers with 10% of the sample being crossed checked by RC.

Patient and Public Involvement statement

No members of the public or patients were involved in this research.

RESULTS

Search results

Systematic searches in 4 databases and in 6 journals performed in March 2021 (updated in March 2022) identified 312 citation. After duplicate removal, 203 potentially relevant abstracts were screened, subsequently 78 articles were identified for full text screening. A further 16 abstracts were identified for full text screening following citation and reference searching. Twenty-eight articles were selected for inclusion. These comprised 15 qualitative, 12 quantitative studies and 1 mixed methods study (see PRISMA flow diagram in Figure 1 and Figure 3)

A summary of the articles reviewed is included in Supplementary Material (Table 2). Applying the candidacy framework and socio-ecological model, key themes were identified and are presented below. The evidence mapping table (Supplementary Material, Table 3) highlight the candidacy stage and level within the socio-ecological model that the articles addressed, as judged by lead author.

Accessibility defined through a 'candidacy' lens

The candidacy framework (13) proved valuable in assessing how healthcare access has been approached, from a holistic perspective. Treatment acceptance was a key emerging issue where there

For peer review only - http://bmjopen.bmj.com/site/about/guidelines.xhtml
For peer review only

was a gap in understanding. Within treatment acceptance there were multiple aspects – delays accepting, interruptions, choosing alternatives and incompletion or loss to follow up, non-compliance, refusal for referral and non-acceptance of diagnosis. This was a dynamic process.

Although the full candidacy process has been considered in research, there were key gaps in how it had been approached and some aspects warranted further exploration. Seven studies aimed to explore delays with initial contact with cancer services on identification of need (presentation), yet the reasons for eventual non-presentation could not be explored in most studies that were clinic based, as all patients surveyed eventually reached treatment centres. Two studies quantified acceptance as having complete follow up and treatment completion. A further four noted delays and high non-acceptance/loss to follow up but did not formally explore them at an individual psychosocial level as this was not within the research aims. Twelve qualitative studies explored individual barriers to accepting care. However, this was not always the primary focus but emerged in the findings (23,24). As these were sampled from a clinic, they represented patients who eventually (despite delays or interruptions) accessed treatment, so the enablers and barriers in those who ultimately dropped out was unknown. Therefore, in depth qualitative psychosocial information on treatment incompletion was not collected. Although the literature on acceptance was predominantly breast cancer related, there was some limited evidence it occurred in other cancers, but the extent and reasons for this were not explored.

Barriers and enablers of cancer service access interpreted through the socio-ecological model

The findings on enablers and barriers to candidacy for cancer treatment were mapped using the socio-ecological model to consider the Ghanaian health ecosystem. These are summarised in Figure 2.

Intrapersonal

Financial barriers

Inability to afford treatment was reported as a barrier leading to delays in, and non-acceptance of care. This was also noted by traditional herbalists (25) and health workers (3). Yamoah (26) found that it encompassed socioeconomic factors, travel costs and lost work. Although two studies indicated it was a greater barrier in those from a low income,(27,28) Sanuade (29) suggested this was regardless of socioeconomic status. Four studies(23,28–30) demonstrated that high cost of medical treatment led to use of herbal and traditional alternatives. Prioritisation of finances on family led to delays in accepting treatment (23,24,31). Financial barriers impacted negotiation of care(31); those from a lower income were more likely to experience longer wait times (32).

Fears and beliefs about treatment and its outcomes

Obrist(33) found patients who believed in the efficacy of treatment were more likely to complete treatment in single variable analysis, although this was not significant in multivariate models where potential confounding factors were controlled for. Fears and beliefs pose individual level barriers from qualitative finding. This included fear of treatment, medicines, and the outcomes (8,24,28,29,34). For example for breast cancer, loosing breasts, womanhood, female identity (24,28,29). Fears around institutional trauma suggested lack of trust health facilities(29). Healthcare professions deliberately miscommunicated to avoid patient fear and drop out (35).

For peer review only - http://bmjopen.bmj.com/site/about/guidelines.xhtml

Misunderstanding about cancer signs and symptoms

Lack of knowledge about cancer signs and symptoms led to delays in seeking medical treatment (8,24,28,31,36–38). With breast cancer, lumps were not regarded as serious when painless and sometimes considered part of normal tissue (8,24,28,31). This was influenced by beliefs held in the community and within patients' social networks (8,31,36). Lay beliefs were influenced by the terminology used for breast cancer in the local dialect, which led to poor understanding (39).

Social demographics associations

Evidence was conflicting as to whether age, religion and ethnicity impacted stage of diagnosis, wait times and treatment completion, which may associate differently in different cancers given different demographics and natural histories (26,32,33,40–43). As ethnicities often cluster predominantly in different regions, the potential confounding of local health system and environmental should be checked in future studies. Some evidence indicates that lower education is associated with the presenting of larger tumour masses(43,44) and waiting longer for treatment(32), but this was not a consistent influencer, and whether it was associated with treatment acceptance was not explored. One study exploring treatment pathways for young people with chronic diseases suggested community beliefs were more influential than educational status (36). Another found that community beliefs and norms influenced the perceptions of breast cancer regardless of socio-economic status (39). Despite clear financial barriers to treatment; there was no evidence that income status and occupation were associated with presentation and acceptance of treatment, but low income status was associated with increased wait times in one study (32).

Interpersonal

Marital relationships influence treatment seeking

Qualitative evidence in female cancer suggests husbands influence their wives' treatment seeking behaviours and acceptance(8,24,27,29,31,38,39,45), by controlling financial decisions about treatment.

Family prioritisation delayed treatment

Women prioritised other activities linked to their economic, family and social roles, such as working for more money, treating children, and paying school fees. These lead to delays presenting for, negotiating and accepting treatment (8,23,24,28,31). Caring for others meant patients put the needs of others first, neglecting their own health. This aligned with findings on the unaffordability of treatment.

Close support networks influence treatment access

Patients understanding about cancer, its causes, and how they engage with care was influenced by close friends and family (8,23,28,34,36–38,45). Misinformation could lead to late presentation, delay help seeking, and use of alternatives (8,23,36,37). For women, lack of husband, family and friend support delayed treatment seeking (27,45). Familial financial support was an enabler for some to seek treatment(23,45) whereas family neglect may impede access (3).

Institutional

Healthcare personnel as gatekeepers to medical and alternative care

Poor detection at primary health facilities, community pharmacy and private settings, may have delayed diagnosis (3,8,23,31,36,44–46). Some women sought over the counter (OTC) medications for

For peer review only - http://bmjopen.bmj.com/site/about/guidelines.xhtml

pain management (37). Seeking assistance from someone other than a nurse or doctor was associated with a larger mass at diagnosis for breast cancer, which could include a diverse mix of pluralist and community supports (44). Some health professionals also advised herbal alternatives, delaying medical cancer treatment (29). A mixed method study found an inability for facilities to diagnose cancer, improper documentation and filing of patient folders and workload – likely exacerbated by a shortage of healthcare workers trained in oncology outside of major tertiary centres (3). In agreement, qualitative studies with patients found misdiagnosis was common (37,38). There were delays due to: the complex referral process, waiting a long time to get results, having to go to many hospitals and laboratories to be diagnosed, and consultant rescheduling (37,38). Delays between referral and starting radiotherapy were suggested to be due to resource availability, whilst irregular medicine supply also meant patients had to source medicine outside hospitals at high cost (23,47). Patients showed negative perceptions of the care system and professionals (27,29,29,45). Patients perceived treatment delays due to workforce shortage, hospital machines breaking, and medicines shortages (29). These beliefs appear to contribute to a lack of confidence and trust in the health system. Non-completing patients were more likely to harbour negative views such as that the unavailability of cancer medicines delayed their treatment (33). Doubts in the efficacy and disappointment with conventional treatment created barriers to seeking treatment (27) and influenced use of pluralistic treatments (30). Fear of radiation led some not to receive clinically recommended treatment (30).

Community

The body of literature showed the strong role community beliefs and norms played in shaping access to cancer care. These were interconnected with personal perceptions and health system factors.

Spiritual and traditional beliefs about cancer causes and treatment

In an overwhelming majority of the literature, patients assigned their cancer diagnosis to spiritual causes, which led patients to seek traditional herbal and spiritual treatments, delaying presentation and interrupting medical treatment at multiple stages. This was associated with financial barriers to conventional treatment,(23,28,29) advice from supports such as spouse(45), health workers(29), religious messages(8,29), community networks and beliefs (8,29,31,34,36,39). Additionally alternative therapies were often perceived as more available and acceptable, seen as efficacious (27,28,39,47).

Religiosity plays a diachronous role

Religious beliefs, messages and leaders influenced alternative therapy use(8,29) and caused delays (8,29,37). Yet, religious leaders were identified within patients' trusted support networks(8) and their advice facilitated medical presentation(8,24). Some studies found the church played a supportive role, encouraging women to present at services and providing financial assistance to low-income families (24).

Gender and identity norms

For women with breast cancer, mastectomy was associated with a fear of 'diminished sexuality and femininity' which led many women to delay treatment after seeing an oncologist (24,28,29,31). Unaddressed fears about fertility loss may have increased drop out (35). However, the barriers around identity may differ in other tumours and population groups.

Community networks influenced beliefs and norms

Common misconceptions, beliefs and behaviours held by patients were reinforced by community and social network beliefs (8,29,31,36). Interlinked with community beliefs about cancer is self and socially

For peer review only - http://bmjopen.bmj.com/site/about/guidelines.xhtml

experienced stigma due to the cause of the disease being spiritual: a curse, mis-endeavour, or the patient being a witch(24,27,39). This led to patients seeking traditional herbal and spiritual treatments, whilst creating shame and secrecy. A retrospective survey found patients who did not complete treatment were more likely to answer they do not know if they were fearful of their community response. However, this likely reflects uncertainty by next of kin respondents, who were substantially higher in 'non-completing treatment' groups (33).

Policy

National Health Insurance Scheme (NHIS) inclusion of cancer care

Based on the financial barriers to treatment reported by patients and healthcare workers, lack of cancer care inclusion within the NHIS was inferred to lead to treatment refusal and delays (3). This was the case for cancers not covered by the scheme(26,42,48), as well as breast(23,24) and cervical cancer (27,40). Breast cancer patients were unanimously frustrated that the NHIS did not cover substantial amounts of treatment and discussed the huge financial burden, especially of chemotherapy drugs (23,24). This meant some women could not start treatment on time (34). This was aggravated by medicines stock-outs(3,23,29,47), requiring purchase elsewhere at additional cost (23,47). Not being insured was significantly associated with a shorter wait time for breast cancer treatment(32), which could reflect preferential treatment to those paying upfront due to delays in the administrative process of reimburse NHIS funding. Knowledge of hormone receptor status predicted complete treatment follow up, as this service is offered at a cost (41). Healthcare professionals acknowledged costs were barriers for patients but struggled to broach such topics (35).

Integration of pluralistic care approaches

Given the prominent roles of traditional, herbal and spiritual care, improved integration with the orthodox health system could improve patient access (25,28).

Thirty eight percent of clinical workers surveyed in Ghana attribute treatment disruptions to traditional medicines use (3). Reported use of traditional healers was a significant predictor of late presentation after other variables were controlled for (44). An assessment of factors associated with treatment completion found visiting a traditional healer was a significant predictor of not completing treatment (33). Although alternative support could be concurrent to orthodox medical intervention, over 50% of complementary and alternative medicine practitioners surveyed indicated they did not let patients seek other care alongside(25) and 63% of customers said they declined orthodox therapy while using such therapies (30). Nevertheless, one study found that although 12.2% seek alternative therapies, this only partially explains high rates(73.1%) of loss to follow up (41). Whilst traditional herbalists are considered health professionals, with some services integrated into the Ghanaian health system, poor knowledge of cancer causes and symptoms, and treatment and reluctance to refer to other services are barriers to providing their patients with timely appropriate care (25). This is influenced by a perceived reluctance collaborate from other health system components (25). Mburu suggests the interaction between treatment approaches is non-linear as acts at multiple pathway stages (37).

Critique of the evidence

As part of the CIS approach, a critique of the literature was conducted to identify themes in methods, assumptions, theories, and analysis to identify methodological limitations and research gaps for further studies. A summary of the studies characteristics is displayed in Figure 3.

For peer review only - http://bmjopen.bmj.com/site/about/guidelines.xhtml

This review accurately represents evidence in Ghana, which has a high breast cancer contribution. Most studies focus either on presentation delays or treatment interruptions, barriers and treatment delays. Although many studies note treatment incompletion and loss of follow up, only two assess this directly and they note challenges in data collection,(33,41) there are no qualitative studies exploring definitive incompletion. Eighty eight percent (25/28) of studies were based in tertiary treatment clinics in the Greater Accra and Ashanti region, so may not reflect those in other regions. As 89% of qualitative studies sampled purposively from tertiary clinics, this led to biased sampling. They omit those who did not attend, those who dropped out without contact or cannot reach treatment centres. Sampling patients in clinics represents those who eventually presented for and accepted treatment. Barriers could be experienced differently in the under-represented population. As some studies had a small sample and were predominantly Christian, there was limited ethnographic diversity, which may influence generalisability. Findings on the social demographic traits linked to barriers accessing treatment were inconclusive.

Hypothetical inquiry was found to be common across studies. This may lead to error if an individual is not able to accurately predict their behaviour in an unknown situation(49), which may be the case as cancer is stigmatised and not talked about openly (50). Further work should explore and seek to understand the impact cognitive bias may have when using hypothetical situations. The gaps in research identified are summarised in the box below.

Critiquing the evidence body identified several themes and research gaps:

- Most studies are situated at tertiary clinics, so may not represent rural regions
- Sampling from tertiary clinics means populations who do not present, negotiate the referral pathway from local services and eventually choose treatment are not represented. Understanding what influences access in these missing populations warrants further study
- Treatment drop-out is frequently observed but the reasons for this are not sufficiently explored.
- As most studies focus on breast cancer, there is need to understand the extent of treatment drop out across all cancers and which factors influence this

DISCUSSION

This study used CIS to review multiple types of qualitative and quantitative evidence from literature on access to cancer treatment services in Ghana. Applying 'candidacy' enabled the dynamic and continuous process of accessing, negotiating, and accepting treatment to be explored within the Ghanaian social, economic and policy environment. It highlighted determinants of cancer treatment service access in Ghana are interlinked and within each stage of the socio-ecological model. There is a research gap in understanding the determinants of accessing treatment in the most vulnerable populations due to methodological limitations.

Through this approach, we were able to critique the literature, highlighting trends in methodology and gaps in evidence for future study. The CIS enabled detailed context-specific insights as well as identifying limitations in research approaches, data collection and acquisition challenges to inform future research. A reflective and iterative approach to broaden the breadth of evidence, interpretation and assimilation, was taken. This was particularly valuable to for integrity in research in West African,

For peer review only - http://bmjopen.bmj.com/site/about/guidelines.xhtml

due to epistemic injustice in how knowledge is perceived and interpreted (51). This is the first study to have explored the applicability of the candidacy framework of healthcare access to a Ghanaian setting. Although this has proven valuable in other African settings(16), this model was developed in a UK setting, therefore it was important to acknowledge pre-existing bias in perspective and thus to critically assess this framing and how it might impact the interpretation of results (51). Selecting globalised frameworks (over those locally synthesised) can lead to interpretive marginalisation, which we sought to avoid. Only published academic articles were included in this study, which may overlook other forms of evidence, including locally generated and day-to-day working understandings. However, this was minimised through an iterative approach to include multiple databases, targeting local journals and supplementary searches. Another challenge was the vague and broad nature of terms relating to 'accessing' healthcare. There was a need to balance breadth of search with pragmatism to formulate a multi-step search strategy. The interpretation of the evidence was primarily conducted by the lead author, so will reflect their inherent biases in world view (22).

The evidence highlighted financial barriers to cancer treatment access, which interacted with cultural factors and societal influences, such as norms around managing household finances, prioritisation and cultural acceptance of alternative medicines. Globally, catastrophic costs (defined as greater than 40% household effective income) due to non-communicable disease health expenditure are prevalent in LMICs, leading to individuals not taking medicines and impoverishment(52). This exacerbates inequities, having the greatest impact on the poor and leads to detrimental coping strategies(53).

Poor patient clinician relationships have been found to lead people to seek traditional medicines alternatives in Ghana (54). Traditional medicines practitioners were seen to offer more patient-centric, holistic care which was more comforting. A lack of trust was noted for orthodox facilities, which may reflect cultural beliefs as well as past healthcare experiences. An evidence synthesis across LMICs found modern medicines viewed to be harmful and ineffective; suspicion and mistrust of biomedicine lengthened delay and led to alternative use, and the impact of this may be exacerbated in the most vulnerable(50). A qualitative exploration of influences on traditional medicines use in Ghana found their 'pull' by accessibility and alignment with cultural beliefs, whereas scepticism of biomedicine may push people from orthodox healthcare(54).

At a community level, spiritual beliefs about the origin of cancer interacted with personal perceptions at the individual level. Notions and beliefs typically held in the community can be ascribed as lay explanatory models of disease. In accordance with Kleinman(55), lay models of disease can differ from biomedical models based on social experiences and impact how individuals interpret and act on their condition. Community factors influenced explanatory models for hypertension in rural northern Ghana and impact treatment access(56). Similarly, in this review, explanatory models created stigma leading to secrecy and selection of traditional medicines over biomedical intervention.

At a policy level, two key themes where reforms could improve cancer treatment uptake stood out: 1) greater inclusion of cancer treatments within the NHIS, 2) enhanced integration of traditional medicines to provide complementary options to medical care for cancer.

Despite the NHIS aim of achieving universal health coverage for all, there has been notable disparity, with the lowest coverage concentrated in the poor (57,58). Multidimensional barriers due to poverty, dissatisfaction and distrust of the health service and staff may prohibit enrolment (59). Furthermore, catastrophic spending due to out of pocket costs remains high (60). The NHIS plan seeks to cover a

For peer review only - http://bmjopen.bmj.com/site/about/guidelines.xhtml

considerable amount of the local disease burden and since its nascence the inclusion list has been revised in response to transitions in disease burden and advancing treatments (61,62). Despite breast and cervical cancer in theory being covered(63), it is widely noted women still face considerable financial burden, which this study further highlights. The burden in men however remains less clearly mapped. The high expense of cancer medicines poses a challenge to decision-makers, who must weight costs and benefits when deciding on how to invest health budgets. Approaches such as the health technology assessment platform recently established in Ghana could help prioritise high-cost medicines (64,65).

Another policy area identified in this review was coordination between medical services and traditional medicines. Studies in Ghana have shown traditional medicines users are more likely to be poor and not insured on the NHIS (66). The NHIS currently provides services through a plural system of public, faith-based, governmental and private facilities, and includes some traditional medicines (57). Although lack of harmonisation of traditional medicines with the healthcare system (67) was reported in this study, there have been multiple reforms to this end since the Traditional and Alternative Medicine Directorate was established under the Ghanaian Ministry of Health in 2001 (68). Still coordination is hindered by a lack of professional respect from other health professionals (67). Co-current use of alternative forms of care has been found in pregnant women in Ghana. It was highlighted the individual psychosocial and emotional support they provide, which this study found can be lacking patient interactions with orthodox medicine, despite being key for candidacy in cancer treatment (15,69).

The African Union has calls to recognise the importance of traditional medicines and a Global Centre for Traditional Medicines has been established in India (70,71). However, there remain challenges in how traditional and complementary medicines are perceived and deemed efficacious. Barry(72) suggests differently constructed modes of evidence are needed for traditional medicines, as scientific evidence through clinical trials offers a reductionist, narrowly defined view of evidence, that overlooks the role of lived-in social experiences, advocating for the 'expanded epistemology of science' (73). Future policies should seek to improve the affordability and quality of publicly provided medical care whilst harmonising with complementary treatments that align with community beliefs.

Future research is needed to address the research gaps identified. First, to understand the extent of treatment non-adherence across call cancers are what individual, social and health system factors impact this. Second, there are methodological limitations in understanding the views of those who do not attend clinics, which may represent the most vulnerable. Researchers should seek approaches to overcome this which are suitable within the local context.

Acknowledgements

Additionally, the authors would like to thank the School of Health and Related Research Library team for their expertise in designing the search protocol, as well as all researcher staff and graduate at the Universities of Sheffield and Ghana who commented or provided feedback on preliminary results.

Funding

This research was funded by Wellcome as part of a PhD studentship [108903/B/15/Z].

Competing interests

For peer review only - http://bmjopen.bmj.com/site/about/guidelines.xhtml

The authors declare no conflicts of interest in undertaking this research.

Data sharing statement

Data sharing not applicable as this is a review of literature, and no datasets were generated.

Contributorship statement

CT, RAk, LG and RC conceptualised the study. CT led the in designing the study, conducted literature search to collect data, perform the data analysis and wrote the first draft. RAk and LG contributed to selecting literature for inclusion in the study and advised on data collection and analysis. RC, RAk, RAr and LG reviewed the initial and final draft manuscript and made intellectual inputs to improve quality. All authors read and approved the final manuscript.

CT is responsible for the overall content as guarantor and accepts full responsibility for the work and controlled the decision to publish.

The corresponding author (CT) attests that all listed authors meet authorship criteria and that no others meeting the criteria have been omitted.

Ethical approval

This study does not involve human participants and did not require ethical approval.

Figure legends / captions

Figure 1: PRISMA

*Italicised numbers are from search update conducted March 2022 to identify newly published literature. Diagram adapted from From: Page MJ, McKenzie JE, Bossuyt PM, Boutron I, Hoffmann TC, Mulrow CD, et al. The PRISMA 2020 statement: an updated guideline for reporting systematic reviews. BMJ 2021;372:n71. doi: 10.1136/bmj.n71. For more information, visit: http://www.prisma-statement.org/

Figure 2: Key influences on candidacy for cancer treatment mapped against the socio-ecological model(10–12)

Figure 3: Summary of characteristics of included studies

References

1. World Health Organization. WHO report on cancer: setting priorities, investing wisely and providing care for all [Internet]. World Health Organization; 2020 [cited 2020 Mar 28]. Available from: https://apps.who.int/iris/handle/10665/330745

2. Bray F, Ferlay J, Soerjomataram I, Siegel RL, Torre LA, Jemal A. Global cancer statistics 2018: GLOBOCAN estimates of incidence and mortality worldwide for 36 cancers in 185 countries. CA Cancer J Clin. 2018;68(6):394–424.

3. Ayandipo O, Wone I, Kenu E, Fasehun LK, Ayandipo O, Gaye F, et al. Cancer ecosystem assessment in West Africa: health systems gaps to prevent and control cancers in three countries: Ghana, Nigeria and Senegal. Pan Afr Med J. 2020;35(101517926):90.

For peer review only - http://bmjopen.bmj.com/site/about/guidelines.xhtml

4. Adanu K. Household treatment cost of breast cancer disease and cost coping strategy of women, presenting at the Korle Bu Teaching Hospital [Dissertation]. [Accra]: University of Ghana; 2019.

5. Bonsu AB, Aziato L, Clegg-Lamptey JNA. Living with Advanced Breast Cancer among Ghanaian Women: Emotional and Psychosocial Experiences [Internet]. International Journal of Palliative Care. 2014 [cited 2019 Dec 2]. Available from: https://www.hindawi.com/journals/ijpc/2014/403473/abs/

6. Wang, Huihui, Nathaniel Otoo, and Lydia Dsane-Selby. 2017. Ghana National Health Insurance Scheme: Improving Financial Sustainability Based on Expenditure Review. World Bank Studies. Washington, DC: World Bank. doi:10.1596/978-1-4648-1117-3. License: Creative Commons Attribution CC BY 3.0 IGO7.

7. Blanchet NJ, Fink G, Osei-Akoto I. The Effect of Ghana's National Health Insurance Scheme on Health Care Utilisation. Ghana Med J. 2012 Jun;46(2):76–84.

8. Bonsu AB, Ncama BP. Recognizing and appraising symptoms of breast cancer as a reason for delayed presentation in Ghanaian women: A qualitative study. PloS One. 2019;14(1):e0208773.

9. Yarney J, Ohene Oti NO, Calys-Tagoe BNL, Gyasi RK, Agyeman Duah I, Akoto-Aidoo C, et al. Establishing a Cancer Registry in a Resource-Constrained Region: Process Experience From Ghana. JCO Glob Oncol. 2020 Apr 17;6:JGO.19.00387.

10. Bronfenbrenner U. Ecological systems theory. In: Six theories of child development: Revised formulations and current issues. London, England: Jessica Kingsley Publishers; 1992. p. 187–249.

11. The Socioecological Model as a Framework for Exploring Factors Influencing Childhood Immunization Uptake in Lagos State, Nigeria. 2021 Jan 8 [cited 2021 Apr 20]; Available from: https://www.researchsquare.com

12. Ngwenya N, Nkosi B, Mchunu LS, Ferguson J, Seeley J, Doyle AM. Behavioural and socio-ecological factors that influence access and utilisation of health services by young people living in rural KwaZulu-Natal, South Africa: Implications for intervention. PLoS ONE [Internet]. 2020 Apr 14 [cited 2021 Apr 20];15(4). Available from: https://www.ncbi.nlm.nih.gov/pmc/articles/PMC7156071/

13. Dixon-Woods M, Cavers D, Agarwal S, Annandale E, Arthur A, Harvey J, et al. Conducting a critical interpretive synthesis of the literature on access to healthcare by vulnerable groups. BMC Med Res Methodol. 2006 Jul 26;6(1):35.

14. van der Boor CF, White R. Barriers to Accessing and Negotiating Mental Health Services in Asylum Seeking and Refugee Populations: The Application of the Candidacy Framework. J Immigr Minor Health. 2020 Feb;22(1):156–74.

15. Tookey S, Renzi C, Waller J, von Wagner C, Whitaker KL. Using the candidacy framework to understand how doctor-patient interactions influence perceived eligibility to seek help for cancer alarm symptoms: a qualitative interview study. BMC Health Serv Res. 2018 Dec 4;18:937.

For peer review only

16. Adeagbo O, Herbst C, Blandford A, McKendry R, Estcourt C, Seeley J, et al. Exploring People's Candidacy for Mobile Health–Supported HIV Testing and Care Services in Rural KwaZulu-Natal, South Africa: Qualitative Study. J Med Internet Res. 2019 Nov 18;21(11):e15681.

17. Booth A, Noyes J, Flemming K, Gerhardus A, Wahlster P, van der Wilt GJ, et al. Structured methodology review identified seven (RETREAT) criteria for selecting qualitative evidence synthesis approaches. J Clin Epidemiol. 2018 Jul;99:41–52.

18. Vélez CM, Wilson MG, Lavis JN, Abelson J, Florez ID. A framework for explaining the role of values in health policy decision-making in Latin America: a critical interpretive synthesis. Health Res Policy Syst. 2020 Sep 7;18(1):100.

19. Khalid AF, Lavis JN, El-Jardali F, Vanstone M. Supporting the use of research evidence in decision-making in crisis zones in low- and middle-income countries: a critical interpretive synthesis. Health Res Policy Syst. 2020 Feb 18;18(1):21.

20. Jarvis T, Scott F, El-Jardali F, Alvarez E. Defining and classifying public health systems: a critical interpretive synthesis. Health Res Policy Syst. 2020 Jun 16;18(1):68.

21. Booth A, Noyes J, Flemming K, Moore G, Tunçalp Ö, Shakibazadeh E. Formulating questions to explore complex interventions within qualitative evidence synthesis. BMJ Glob Health. 2019 Jan 1;4(Suppl 1):e001107.

22. Bryman A. Social research methods. 4th ed. Oxford ; New York: Oxford University Press; 2012. 766 p.

23. Agbokey F, Kudzawu E, Dzodzomenyo M, Ae-Ngibise KA, Owusu-Agyei S, Asante KP. Knowledge and Health Seeking Behaviour of Breast Cancer Patients in Ghana. Int J Breast Cancer. 2019;2019:5239840.

24. Martei YM, Vanderpuye V, Jones BA. Fear of Mastectomy Associated with Delayed Breast Cancer Presentation Among Ghanaian Women. The oncologist. 2018;23(12):1446–52.

25. O'Brien KS, Soliman AS, Annan K, Lartey RN, Awuah B, Merajver SD. Traditional herbalists and cancer management in Kumasi, Ghana. J Cancer Educ Off J Am Assoc Cancer Educ. 2012;27(3):573–9.

26. Yamoah K, Beecham K, Hegarty SE, Hyslop T, Showalter T, Yarney J. Early results of prostate cancer radiation therapy: an analysis with emphasis on research strategies to improve treatment delivery and outcomes. BMC Cancer. 2013;13(100967800):23.

27. Binka C, Nyarko SH, Awusabo-Asare K, Doku DT. Barriers to the Uptake of Cervical Cancer Screening and Treatment among Rural Women in Ghana. BioMed Res Int. 2019;2019(101600173):6320938.

28. Asoogo C, Duma SE. Factors contributing to late breast cancer presentation for health care amongst women in Kumasi, Ghana. Curationis. 2015;38(1).

29. Sanuade OA, Ayettey H, Hewlett S, Dedey F, Wu L, Akingbola T, et al. Understanding the causes of breast cancer treatment delays at a teaching hospital in Ghana. J Health Psychol. 2021;26(3):357–66.

For peer review only - http://bmjopen.bmj.com/site/about/guidelines.xhtml

30. Yarney J, Donkor A, Opoku SY, Yarney L, Agyeman-Duah I, Abakah AC, et al. Characteristics of users and implications for the use of complementary and alternative medicine in Ghanaian cancer patients undergoing radiotherapy and chemotherapy: a cross- sectional study. BMC Complement Altern Med. 2013;13(101088661):16.

31. Agbeko AE, Arthur J, Bayuo J, Kaburi BB, Kyei I. Seeking healthcare at their 'right' time; the iterative decision process for women with breast cancer. BMC Cancer. 2020 Oct 19;20(1):1011.

32. Dedey F, Wu L, Ayettey H, Sanuade OA, Akingbola TS, Hewlett SA, et al. Factors Associated With Waiting Time for Breast Cancer Treatment in a Teaching Hospital in Ghana. Health Educ Behav Off Publ Soc Public Health Educ. 2016;43(4):420–7.

33. Obrist M, Osei-Bonsu E, Awuah B, Watanabe-Galloway S, Merajver SD, Schmid K, et al. Factors related to incomplete treatment of breast cancer in Kumasi, Ghana. Breast Edinb Scotl. 2014;23(6):821–8.

34. Iddrisu M, Aziato L, Ohene LA. Socioeconomic impact of breast cancer on young women in Ghana: A qualitative study. Nurs Open. 2021 Jan;8(1):29–38.

35. Agyemang LS, Foster C, McLean C, Fenlon D, Wagland R. The cultural and structural influences that 'hide' information from women diagnosed with breast cancer in Ghana: an ethnography. BMC Womens Health. 2021 Oct 15;21(1):364.

36. Atobrah D. When darkness falls at mid-day: young patients' perceptions and meanings of chronic illness and their implications for medical care. Ghana Med J. 2012;46(2 Suppl):46–53.

37. Mburu W, Boamah Mensah AB, Virnig B, Amuasi J, Awuah B, Porta C, et al. Pathways to Breast Cancer Diagnosis and Treatment Among Women in Ghana: A Qualitative Study. Womens Health Rep. 2021 Jul 1;2:234–44.

38. Hobenu KA, Naab F. Accessing specialist healthcare: experiences of women diagnosed with advanced cervical cancer in Ghana. Br J Healthc Manag. 2022 Feb 2;28(2):1–11.

39. Asobayire A, Barley R. Women's cultural perceptions and attitudes towards breast cancer: Northern Ghana. Health Promot Int. 2015;30(3):647–57.

40. Nartey Y, Hill PC, Amo-Antwi K, Nyarko KM, Yarney J, Cox B. Characteristics of Women Diagnosed with Invasive CervicalCancer in Ghana. Asian Pac J Cancer Prev APJCP. 2018 Feb 26;19(2):357–63.

41. Scherber S, Soliman AS, Awuah B, Osei-Bonsu E, Adjei E, Abantanga F, et al. Characterizing breast cancer treatment pathways in Kumasi, Ghana from onset of symptoms to final outcome: Outlook towards cancer control. Breast Dis. 2014 Apr 2;34(4):139–49.

42. Asamoah F a., Yarney J, Vanderpuye V, Awasthi S, Venkat P s., Dadzie M a., et al. Definitive Radiation Treatment Patterns and Outcomes for Low Intermediate Risk Prostate Cancer - A Cross Continental Comparative Study between Ghana and the United States of America. Int J Radiat Oncol Biol Phys. 2018 Nov 2;102:e97–e97.

43. Dunyo P, Effah K, Udofia EA. Factors associated with late presentation of cervical cancer cases at a district hospital: a retrospective study. BMC Public Health. 2018 Oct 3;18(1):1156.

For peer review only - http://bmjopen.bmj.com/site/about/guidelines.xhtml

44. Brinton L, Figueroa J, Adjei E, Ansong D, Biritwum R, Edusei L, et al. Factors contributing to delays in diagnosis of breast cancers in Ghana, West Africa. Breast Cancer Res Treat. 2017;162(1):105–14.

45. Aziato L, Clegg-Lamptey JNA. Breast cancer diagnosis and factors influencing treatment decisions in Ghana. Health Care Women Int. 2015;36(5):543–57.

46. Twahir M, Oyesegun R, Yarney J, Gachii A, Edusa C, Nwogu C, et al. Real-world challenges for patients with breast cancer in sub-Saharan Africa: a retrospective observational study of access to care in Ghana, Kenya and Nigeria. BMJ Open. 2021 Mar;11(3):e041900.

47. Salifu Y, Almack K, Caswell G. 'My wife is my doctor at home': A qualitative study exploring the challenges of home-based palliative care in a resource-poor setting. Palliat Med. 2021;35(1):97–108.

48. Dadzie MA, Aidoo CA, Vanderpuye V. Vulva cancer in Ghana - Review of a hospital based data. Gynecol Oncol Rep. 2017;20(101652231):108–11.

49. Fowler, F.J. Jr. Improving Survey Questions: Design and Evaluation [Internet]. Thousand Oaks, CA: Sage; 1995 [cited 2021 Sep 30]. Available from: https://us.sagepub.com/en-us/nam/improving-survey-questions/book4994

50. McCutchan G, Weiss B, Quinn-Scoggins H, Dao A, Downs T, Deng Y, et al. Psychosocial influences on help-seeking behaviour for cancer in low-income and lower middle-income countries: a mixed-methods systematic review. BMJ Glob Health. 2021 Feb;6(2).

51. Bhakuni H, Abimbola S. Epistemic injustice in academic global health. Lancet Glob Health. 2021 Oct 1;9(10):e1465–70.

52. Murphy A, Palafox B, Walli-Attaei M, Powell-Jackson T, Rangarajan S, Alhabib KF, et al. The household economic burden of non-communicable diseases in 18 countries. BMJ Glob Health [Internet]. 2020 Feb 11 [cited 2020 Apr 22];5(2). Available from: https://www.ncbi.nlm.nih.gov/pmc/articles/PMC7042605/

53. Murphy A, McGowan C, McKee M, Suhrcke M, Hanson K. Coping with healthcare costs for chronic illness in low-income and middle-income countries: a systematic literature review. BMJ Glob Health. 2019 Aug 1;4(4):e001475.

54. Gyasi RM, Asante F, Yeboah JY, Abass K, Mensah CM, Siaw LP. Pulled in or pushed out? Understanding the complexities of motivation for alternative therapies use in Ghana. Int J Qual Stud Health Well-Being. 2016;11:29667.

55. Kleinman A. Patients and Healers in the Context of Culture : an exploration of the borderland between anthropology, medicine, and psychiatry [Internet]. Univ of California Press; 1980 [cited 2020 May 19]. Available from: https://www.ucpress.edu/book/9780520045118/patients-and-healers-in-the-context-of-culture

56. Nyaaba GN, Masana L, Aikins A de-Graft, Stronks K, Agyemang C. Lay community perceptions and treatment options for hypertension in rural northern Ghana: a qualitative analysis. BMJ Open. 2018 28;8(11):e023451.

For peer review only - http://bmjopen.bmj.com/site/about/guidelines.xhtml

57. Dake FAA. Examining equity in health insurance coverage: an analysis of Ghana's National Health Insurance Scheme. Int J Equity Health. 2018 Jun 18;17(1):85.

58. Dixon J, Tenkorang EY, Luginaah I. Ghana's National Health Insurance Scheme: Helping the Poor or Leaving Them Behind? Environ Plan C Gov Policy. 2011 Dec 1;29(6):1102–15.

59. Millicent Kotoh A, Aryeetey GC, der Geest SV. Factors That Influence Enrolment and Retention in Ghana' National Health Insurance Scheme. Int J Health Policy Manag. 2017 Oct 17;7(5):443–54.

60. Okoroh J, Essoun S, Seddoh A, Harris H, Weissman JS, Dsane-Selby L, et al. Evaluating the impact of the national health insurance scheme of Ghana on out of pocket expenditures: a systematic review. BMC Health Serv Res. 2018 Jun 7;18(1):426.

61. Ghana NHIS. Ghana NHIS Medicines List 2020 Version 1.0 [Internet]. 2020. Available from https://www.nhis.gov.gh/files/medicineslist(2020).pdf

62. Ghana NHIS. NHIS Review Recommendation — Provide guaranteed Primary, Maternal and Child healthcare for all [Internet]. National Health Insurance Scheme. 2016 [cited 2022 Aug 11]. Available from: http://www.nhis.gov.gh/nhisreview.https://www.nhis.gov.gh/News/nhis-review-recommendation-%E2%80%94-provide-guaranteed-primary,-maternal-and-child-healthcare-for-all-4087aspx

63. Ministry of Health. NHI Policy Framework for Ghana (Revised Version August 2004) [Internet]. 2004. Available from: https://www.ghanahealthservice.org/downloads/NHI_policy%20framework.pdf

64. Ghana's Minister of Health launches the National HTA Steering Committee and calls for HTA institutionalisation in the country [Internet]. iDSI. 2019 [cited 2019 Nov 12]. Available from: https://www.idsihealth.org/blog/ghanas-minister-of-health-launches-the-national-hta-steering-committee-and-calls-for-hta-institutionalisation-in-the-country/

65. Hollingworth S, Gad M. Health technology assessment case study: Ghana. F1000Research [Internet]. 2018 Jul 2 [cited 2019 Nov 12];7. Available from: https://f1000research.com/slides/7-979

66. Amegbor PM, Kuuire VZ, Bisung E, Braimah JA. Modern or traditional health care? Understanding the role of insurance in health-seeking behaviours among older Ghanaians. Prim Health Care Res Dev. 2019;1:e71.

67. Appiah B, Amponsah IK, Poudyal A, Mensah MLK. Identifying strengths and weaknesses of the integration of biomedical and herbal medicine units in Ghana using the WHO Health Systems Framework: a qualitative study. BMC Complement Altern Med [Internet]. 2018 Oct 22 [cited 2020 Mar 27];18. Available from: https://www.ncbi.nlm.nih.gov/pmc/articles/PMC6196414/

68. Ghana Ministry of Health. Traditional Medicines Policy. 2005;15.

69. Dako-Gyeke P, Aikins M, Aryeetey R, McCough L, Adongo PB. The influence of socio-cultural interpretations of pregnancy threats on health-seeking behavior among pregnant women in urban Accra, Ghana. BMC Pregnancy Childbirth. 2013 Nov 19;13:211.

For peer review only - http://bmjopen.bmj.com/site/about/guidelines.xhtml

70. WHO, Africa CDC in joint push for COVID-19 traditional medicine research in Africa [Internet]. Africa CDC. [cited 2021 Sep 28]. Available from: https://africacdc.org/news-item/who-africa-cdc-in-joint-push-for-covid-19-traditional-medicine-research-in-africa/

71. WHO establishes the Global Centre for Traditional Medicine in India [Internet]. [cited 2022 Apr 1]. Available from: https://www.who.int/news/item/25-03-2022-who-establishes-the-global-centre-for-traditional-medicine-in-india

72. Barry CA. The role of evidence in alternative medicine: Contrasting biomedical and anthropological approaches. Soc Sci Med. 2006 Jun 1;62(11):2646–57.

73. Baer HA. Toward an Integrative Medicine: Merging Alternative Therapies with Biomedicine. Walnut Creek, CA: Altamira Press; 2004. 224 p.

For peer review only

For peer review only - http://bmjopen.bmj.com/site/about/guidelines.xhtml

Figure 1: PRISMA

*Italicised numbers are from search update conducted March 2022 to identify newly published literature. Diagram adapted from From: Page MJ, McKenzie JE, Bossuyt PM, Boutron I, Hoffmann TC, Mulrow CD, et al. The PRISMA 2020 statement: an updated guideline for reporting systematic reviews. BMJ 2021;372:n71. doi: 10.1136/bmj.n71. For more information, visit: http://www.prisma-statement.org/

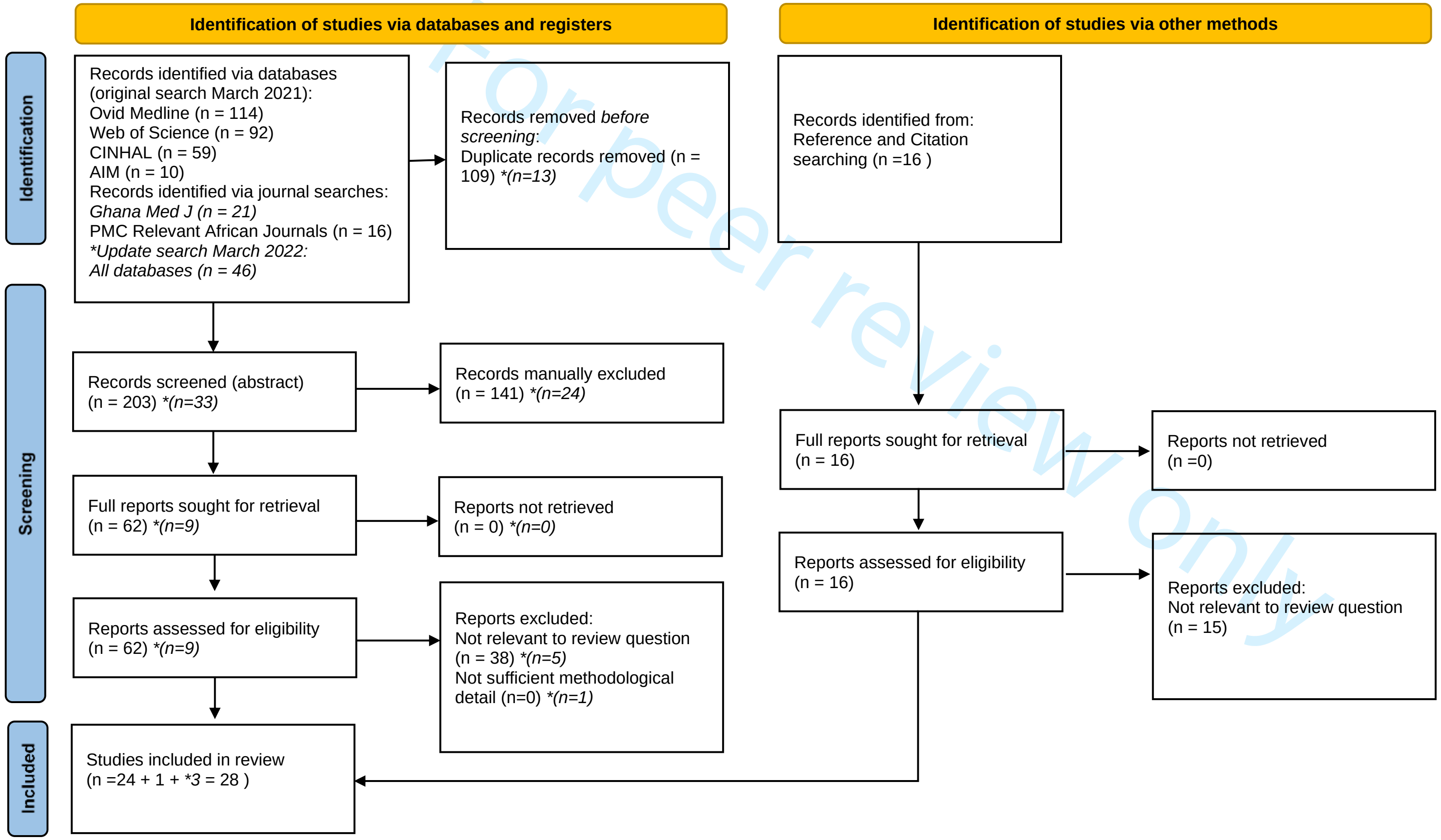

Italicised numbers are from search update conducted March 2022 to identify newly published literature

From: Page MJ, McKenzie JE, Bossuyt PM, Boutron I, Hoffmann TC, Mulrow CD, et al. The PRISMA 2020 statement: an updated guideline for reporting systematic reviews. BMJ 2021;372:n71. doi: 10.1136/bmj.n71. For more information, visit: http://www.prisma-statement.org/

Figure 2: Key influences on candidacy for cancer treatment mapped against the socio-ecological model(10–12)

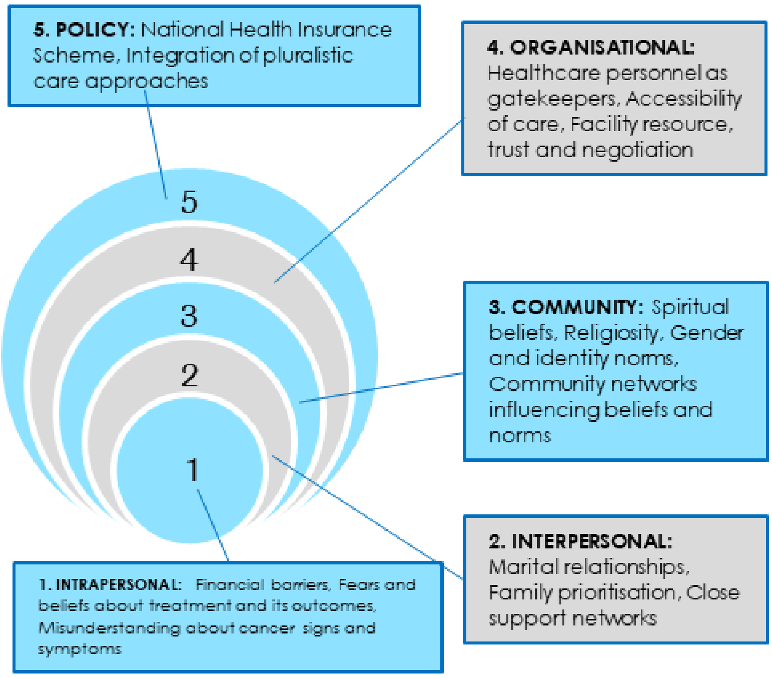

review only

Figure 3: Summary of characteristics of included studies

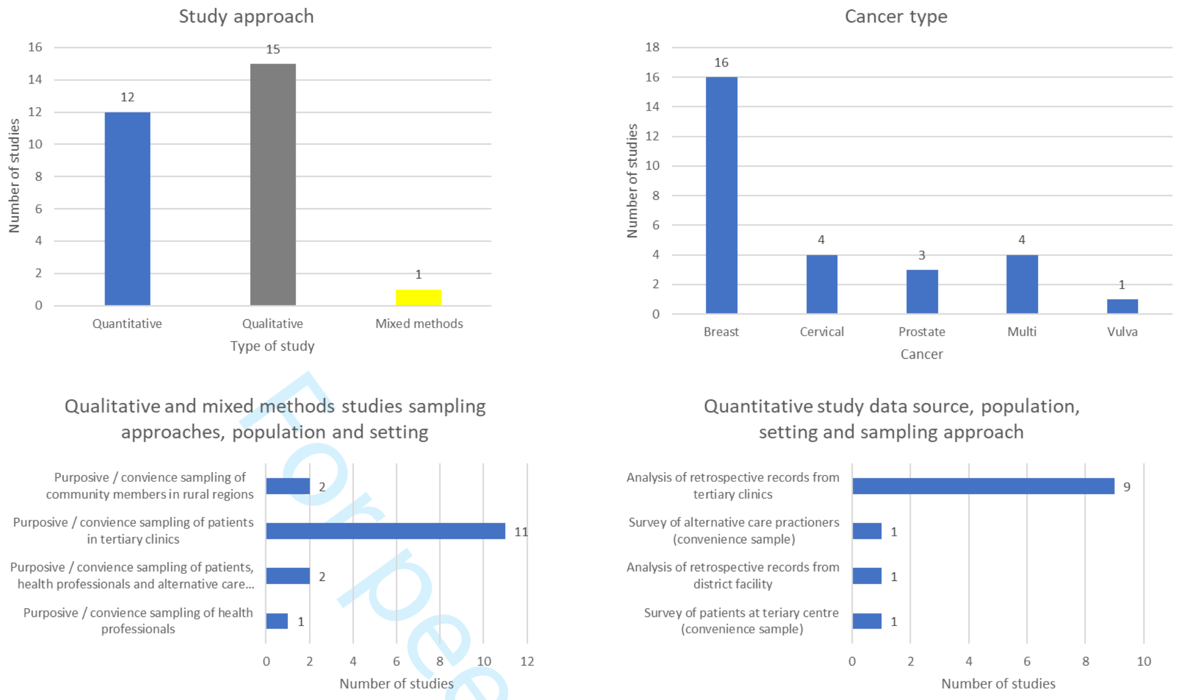

For peer review only

Supplementary Materials

Supplementary Table 1. Systematic search strategy

	**Search topic**		**Ovid MedLine**	**Web of Science**	**CINAHL**	**African Index Medicus**
1	Ghana*		1. exp Ghana/<br>2. Ghana.ti,ab.<br>3. 1 or 2	1. TS=Ghana OR AB=Ghana	1. Ghana	1. Ghana
2	Health service access / patient uptake of services		4. health services accessibility/ or *health services accessibility/ or health equity/ or *healthcare disparities/<br>5.*attitude to health/ or exp health knowledge, attitudes, practice/ or	2. TS=(Health service access)<br>3. AB=((health* NEAR/2 access*) OR (treatment) OR accessibility OR (financ* NEAR/2 (burden* OR impact* OR barrier*)) OR inequalit* OR inequit* OR (social* NEAR/1 economic) OR (social* NEAR/2 (determinant* OR	2. (treatment or therapy or intervention or intervention OR (MH "Health Services Accessibility+"))	-

For peer review only

2

			*treatment adherence and compliance/ or exp patient acceptance of health care/ or exp patient dropouts/ or exp patient satisfaction/ or exp patient preference/ or *treatment refusal/<br>6. ((financ* adj2 (burden or impact or barrier*)) or (health* adj2 access) or inequit* or inequalit* or (social* adj1 economic*) or (social* adj2 (determinant* or disparit* or barrier*)) or accessibility or	disparit* OR barrier*)) OR (catastrophic NEAR/1 cost*))		

For peer review only

			treatment* or (catastrophic adj1 cost*)).ti,ab.<br>7. 4 or 5 or 6			
3	Cancer		8. exp Neoplasms/<br>9. cancer*.ti,ab<br>10. 8 or 9	4. TS=cancer OR AB =cancer*	3. Cancer	2. Cancer
Final search			3 and 7 and 10	1 and (2 or 3) and 4 [Indexes=SCI-EXPANDED, SSCI, A&HCI, CPCI-S, CPCI-SSH, BKCI-S, BKCI-SSH, ESCI, CCR-EXPANDED, IC Timespan=All years]	1 and 2 and 3	1 and 2
Total hits (all time)			145	102	68	10

For peer review only

Total hits in 10 years (since 01/01/2011)		114	92	62 (59 once duplicated removed)	
Date of search		26/03/2021	26/03/2021	26/03/2021	26/03/2021
Repeated search		29/03/2022	29/03/2022	29/03/2022	29/03/2022

Evidence synthesis tables

Supplementary Table 2: Summary of included studies

Author and year	Setting (region)	Study approach	Population and sample	Methods (design, sampling, data analysis)	Phenomenon	Findings	Limitations noted by authors (if any)	Candidacy stage
Binka et al., 2019	Rural community setting and catholic hospital; North Tongu District (Volta region)	Qualitative (deductive)	Sample 1: 15 cervical cancer patients<br>Sample 2: 40 women aged between 30 and 65 registered at the hospital but not screened	Semi-structured interviews and focus group, convenience sampling, deductive thematic analysis	Cervical cancer screening and treatment uptake	- financial burden of treatment due to a lack of government subsidies for those with low household income<br>- expected provision of financial support with husbands' role<br>- Spiritual beliefs about the cause of disease and in the efficacy of traditional medicine led to spiritual and traditional alternatives being used first.<br>Possible institutional-level barriers (privacy, health worker attitudes, potential misdiagnosis) and unavailability of screening and treatment facilities in rural areas	-	Presentation, negotiation, acceptance
Aziato et al., 2015	Tertiary clinic, Accra Region	Qualitative (exploratory descriptive)	12 Ghanaian women who have undergone a mastectomy (speaking English, Twi, or Ewe)	Semi-structured interviews, purposive sampling, thematic analysis	Breast cancer treatment intentions	Treatment decisions are based on knowledge and perceptions and influenced by husbands and family; without their financial and emotion support may led to delays<br>Patients believed that alternative treatment could treat breast cancer, and sought them after diagnosis, which was influenced by husbands' views<br>Patients perceived health systems reason for treatment delays were:	-	Negotiation, acceptance

For peer review only

5

						- incorrect diagnosis at district level<br>- waiting for biopsy results		
Asoogo et al., 2015	Tertiary clinic, Ashanti Region	Qualitative descriptive	30 women diagnosed with breast cancer who presented with Stage II and Stage III	semi-structured interviews, convenience sampling, content data analysis	Breast cancer presentation	- financial barriers, including leading to alternative use<br>- misinformation, considering abnormalities normal development<br>- fear of treatment and outcomes influenced by supports and others in their community's experiences<br>- prioritising care of other<br>- belief in the efficacy of alternative medicines led to seeking first, influenced by affordability<br>- fear of losing female identity and capacity for motherhood	-	Presentation
Martei et al., 2018	Tertiary clinic, Accra Region	Qualitative (inductive)	31 women with breast cancer	Semi structured interviews, purposive sampling, grounded theory	Breast cancer presentation	- financial barriers including prioritisation costs of family and frustration on poor coverage by NHIS<br>- negative husband views<br>- loss of gender identity<br>- misinformation, considering abnormalities harmless, misconceptions that treatment was deadly<br>The church played a supportive role for presentation.	Bias in sample representation (age, religion Christian)<br>Sampling bias	Presentation, acceptance
Agbokey et al., 2019	Tertiary clinic, Ashanti Region	Qualitative descriptive/exploratory	20 patients / 8 caregivers with breast cancer<br>5 health workers<br>2 herbalists	Semi structured in-depth interviews, purposive sampling, thematic analysis	Breast cancer health seeking behaviours	- financial barriers, including prioritising family costs and frustration at poor coverage by NHIS<br>- family support to secure funds<br>- lack of information<br>- beliefs influenced by husbands and family<br>- husbands role in financial decisions<br>- seeking alternative medicines, influenced by affordability and family and friends	Clinic setting may cause participants reluctance to speak openly	Presentation, negotiation, acceptance
Salifu et al., 2021	Tertiary clinic, urban region	Qualitative at 2 time points	Men with advanced prostate cancer (n = 23), family caregivers (n = 23), healthcare professionals (n = 12).	Semi structured interviews at 2 time points, purposive sampling, thematic analysis, Social constructivist theory and interpretivism	Home based palliative care for prostate cancer	- selection of alternative medicines influenced by challenges in accessing medicines and perceived poor supervision by health professionals	Limited sample location<br>Researcher interpretation bias	Acceptance

For peer review only

6

Bonsu et al., 2019	Tertiary clinic, Ashanti Region	Qualitative descriptive/exploratory	11 patients with breast cancer diagnosed at stage II/IV	Semi-structured interviews, purposive sampling, thematic analysis Applying adapted model of health seeking behaviour (Andersen model of total patient delay)	Breast cancer presentation	- poor symptom recognition, considering changes to breasts normal<br>- lack of knowledge on where to seek support<br>-fears and fatalistic perceptions<br>- husbands views<br>- prioritisation of family and work obligations<br>- Misrecognition by health professionals*<br>- Lack of trust in health sector due to perceived mismanagement<br>- spiritual and community held beliefs about cancer led to alternative use (acting at all stages)*<br>- religious leaders supported earlier presentation<br>- some religious messages caused delays<br>- influence of trusted social network (husband, family, religious leaders, health workers in their community, colleagues)	Sample site bias<br>Recall bias	Presentation (predominantly - exceptions*)
Agbeko et al., 2020	Tertiary clinic, Ashanti Region	Qualitative descriptive (deductive)	15 women presenting with stage III/IV breast cancer	Semi-structured interviews, purposively sampling, deductive thematic analysis with priori themes (Andersen Behavioural Model of Health care utilisation)	Breast cancer presentation	- financial barriers to referral process<br>- poor symptom recognition, not regarded as serious, influenced by community beliefs<br>- prioritisation of family roles and finances, influenced by gender identity and roles beliefs<br>- referral delays when first contact not a specialist	Recall bias<br>Unable to follow-up participants for respondent validation<br>Researcher and interviewer bias	Presentation, negotiation, acceptance
Sanuade et al., 2021	Tertiary clinic, Accra Region	Qualitative descriptive (deductive and inductive - exploratory)	20 women who have commenced treatment	4 focus group discussions, purposive sampling following survey, deductive and inductive thematic analysis	Breast cancer treatment delays	- high cost of treatment regardless of social economic status (leading using alternatives)<br>- fear of losing female identity<br>- fear of treatment centre trauma (lack of institution trust)<br>- husbands opinion<br>- impolite treatment by health professionals and perceived corruption<br>- health professionals advising alternative treatments<br>- patients perceived health facility delays including waiting for biopsy results, medicines and workforce shortages, machines breaking<br>- spiritual beliefs led to seeking alternatives, influenced by cost and others (religious leaders, health workers)<br>- community network opinions	Low number of participants	Negotiation and acceptance

For peer review only

7

Atobrah et al., 2012	Tertiary clinic, Accra Region	Qualitative (descriptive)	9 young Ga adults (25-35) with chronic diseases; cancer of the breast, cancer of the ovaries, cancer of the nasopharynx, cancer of the cervix, stroke and chronic renal failure	Multiple narrative interviews and observations (12 each) Purposive sampling (on socio-economic status) Thematic analysis using symbolic interactionism and grief frameworks	Early onset of chronic diseases	- poor symptom recognition given young age<br>- advise to seek alternative therapies from friends/family<br>- misdiagnosis<br>- community beliefs (including of spiritual causes) led to seeking alternative treatments<br>- social networks and community members influenced beliefs and choices	-	Presentation, negotiation, acceptance
Asobayire et al., 2015	(District hospital, unclear) Kassena-Nankana (Navrongo, Upper East Region)	Qualitative (descriptive, inductive)	10 community women (six farmers, two traders and two teachers - aged 25 -56)	Focus group interviews and documentary analysis of current practices, purposive sampling (not stated) for 10 settlements and differing community and socioeconomic status, thematic analysis following an inductive analytical framework	Breast cancer perceptions	- local dialect disease name translation causes misunderstanding<br>- husbands and community leader roles in decision making<br>- community beliefs about the efficacy of traditional medicines and spiritual beliefs lead to alternatives being sought first<br>- potential for positive influence of religious and community leaders and female groups, if engaged	-	Presentation (predominantly)
Iddrisu et al., 2021	Three hospitals in Accra (regional, military and university)	Qualitative (descriptive exploratory)	12 young patients (15-49 year) with breast cancer	Semi structured interviews, purposive and snowball sampling, thematic analysis	Breast cancer socio economic impact	- financial barriers to treatment (including as NHIS did not cover costs)<br>- Negative misbeliefs about treatment, influenced by friends, family and community gossip<br>- spiritual beliefs held in the community led to seeking alternatives, influenced by friends and family	-	Acceptance
Ayandipo et al., 2020	With 3 country analysis: 3 tertiary clinics, four regional, four district facilities (1 private)	Mixed methods cross sectional study	Stakeholders in NCD programme, MoH and CSOs Heads of the selected facilities or units and cancer specialists (120 health workers in Ghana, 10%	Standardised questionnaire, desk based literature review, Thematic analysis Gap analysis using WHO framework for health system strengthening with the 6 pillars	Gaps in cancer control	- patient unaffordability of treatment due to lack of coverage on the NHIS<br>- neglect by families<br>- misdiagnosis, improper documentation and staff shortages, particularly outside of major tertiary centres<br>- shortages of medicines, commodities, and machine breakdown<br>- patient preference for traditional medicines	-	Negotiation and acceptance

For peer review only

8

			oncology qualified)					
Nartey et al., 2018	Tertiary clinics in Accra and Ashanti region	Quantitative (retrospective observational)	1725 confirmed invasive cervical cancer cases	Retrospective review of patient record, hospital population sampling, χ2 and logistic (multivariate)regression of factors influencing stage of diagnosis	Cervical cancer stage at diagnosis	- treatment non acceptance for financial reasons<br>- marital status (widowed, divorced, other), ethnicity and (increased) age associated with late diagnosis	Inconsistency, errors and missing data in record keeping<br>Difficulties following up patients/family	Presentation (predominantly)
Scherber et al., 2014	Tertiary clinic in Ashanti region	Quantitative (retrospective observational)	597 breast cancer patients	Review of patient records hospital population sampling, uni and multivariate logistic regression of factors influencing complete treatment follow up	Breast cancer incomplete follow up	- weak positive trend between age and stage diagnosis<br>- no differences in presentation stage whether attended clinic directly or referred from elsewhere<br>- 12.2% patients sought alternative therapies although this does not explain high rates of treatment interruptions/incompletion (73.1% loss to follow up)<br>- presence of hormone status report associated with complete treatment follow up, inferred to reflect the capacity to pay for tests and thus treatment	Inconsistencies with record keeping and missing data Reported barriers only known from those who return for treatment (those who report barriers were more likely to complete treatment)	Acceptance (predominantly, also presentation)
Asamoah et al., 2018	Tertiary clinic in Accra region	Quantitative (retrospective cohort)	1,074 patients with prostate cancer	Retrospective review of records, hospital population sampling, descriptive statistics (χ2 and Fisher's exact tests, Mann-Whitney U tests) and survival analysis	Prostate cancer treatment	- higher median age for presenting with advanced disease	Missing data<br>Hospital records excluded those not able to access	Presentation
Yamoah et al., 2013	Tertiary clinic in Accra region	Quantitative (retrospective cohort)	379 patients with prostate cancer	Retrospective review of patient history, hospital population sampling, descriptive statistics, non-parametric significance tests (due to data skew), survival analysis	Prostate cancer treatment	- treatment non acceptance due to direct and indirect costs being unaffordable, fear of radiation therapy and spiritual beliefs<br>- age was not significantly associated with diagnosis stage	-	Acceptance

For peer review only

9

Dedey et al., 2016	Tertiary clinic in Accra region	Quantitative (retrospective observational)	205 treated breast cancer patients	Retrospective review of records and patient questionnaire, all patients treated (May - Dec 2013), descriptive statistics, non-parametric tests, Poisson multivariate regression on wait time	Breast cancer waiting time	In a multivariate Poisson regression wait time increased with:<br>- low-income low education, low income, Akan ethnicity, aged 50 or over, marital status (single) but not religion<br>- biopsy time<br>- being insured on NHIS (inferred to be due to delays in recouping funds)<br>- Perceived receipt of adequate information, other perceptions of health workers and service were not significant	Sample representation bias<br>Recall bias	Negotiation
Brinton et al., 2017	Three tertiary clinics in Accra and Ashanti region	Quantitative (retrospective observational)	1,184 patients with malignant breast cancer	Retrospective review of case control study data through structured patient interview, factors influencing tumour mass assessed in multivariate parametric model	Breast cancer diagnosis	In a multivariate model, larger mass at diagnosis correlated with:<br>- low education<br>- marital status (widowed/divorced)<br>- seeking assistance from someone other than a doctor or nurse<br>- use of traditional medicines<br>travel and financial difficulties in seeking care were not significant	Limited sampling catchment may limit possible impact of treatment distance	Presentation
Obrist et al., 2014	Tertiary clinic in Ashanti region	Quantitative (Retrospective case control)	141 women/next of kin with breast cancer	Retrospective review of medical records and semi structured interviews, purposive sampling, statistical analysis by parametric t-tests and non-parametric univariate analysis, multivariate model accounting for confounders using logistic regression	Breast cancer treatment incompletion	- non completing patients were more likely to believe they will not respond, but this was not significant in the LR model<br>- practicing Islam was a significantly higher in non-completing group<br>- age was not significant<br>- explored health systems factors were not significant predictors of completion<br>- seeing a traditional healer after any visit was a significant predictor of not completing after the combined analysis<br>- more non completers believed a traditional healer was better at managing cancer (significant at 10% level)<br>- Understanding what the NHIS cover for breast cancer included was significant predictor of completing treatment	Missing contact information and patient follow up<br>Recall bias by next of kin (more likely in DNC group)	Acceptance
Twahir et al., 2021	Two tertiary clinics in Accra region (as part of a study across	Quantitative (retrospective observational)	299 breast cancer patients undergoing treatment	Retrospective review of patient records in standard collection framework, hospital population sampling, descriptive statistical analysis	Breast cancer access to care	Paying out of pocket costs was associated with receiving more treatment cycles	-	Negotiation and acceptance

10

	Ghana, Kenya and Nigeria)							
O'Brien et al., 2011	Community setting, Ashanti region	Quantitative (descriptive)	42 Traditional Medicine Practice Council (TMPC) registered traditional practitioner	Semi-structured interviews, convenience sampling, descriptive analysis	Traditional herbalists' role in cancer management	Traditional herbalists perceive patient barriers to access include:<br>- cost<br>- lack of knowledge of cancer and fear of treatment<br>- distance and insufficient numbers of health centres<br>- stigma<br>Traditional herbalists poor knowledge of cancer and treatment, and reluctance to refer to other services prevent patients receiving timely care<br>This is influenced by other health professionals' reluctance to collaborate and lack of integration into the health system	-	Presentation, negotiation, acceptance (not specified)
Dadzie et al., 2017	Tertiary clinic in Accra region	Quantitative (retrospective observational)	70 vulva cancer cases	Retrospective review all hospital cases, hospital population sampling, descriptive statistics	Vulva cancer treatment	Cost influenced patient choice (other access barriers were inferred)	-	Acceptance (other access barriers were inferred)
Dunyo et al., 2018	Oncology clinic, Christian hospital (Battor), Volta Region	Quantitative (retrospective observational – cross-sectional analytical)	157 cervical cancer patients	Retrospective review all hospital cases, hospital population sampling, descriptive statistics, non-parametric significance tests, logistic regression of influences on late presentation	Cervical cancer presentation	Education (low) was a significant predictor of late presentation<br>Age was not significant	Missing data, retrospective nature prevented follow up	Presentation
Yarney et al., 2013	Tertiary clinic in Accra region	Quantitative (cross-sectional descriptive)	98 cancer patients	Questionnaire, convenience sampling, descriptive analysis	CAM use in cancer patients	Comparatively high cost of conventional treatment meant only CAM treatment was feasible<br>Lower age, decreased education and being married made it more likely to use CAM in logistic regression model, CAM use influenced by friends and family	Non probabilistic sampling<br>Single institution sample<br>Low response rate<br>Sample bias: excluded those who use CAM but not attending hospital	Acceptance

For peer review only

11

Mburu 2021	Tertiary clinic, Ashanti Region	Qualitative (empircal phenomenological approach)	31 women with breast cancer	Purposive sampling, semi-structured interviews, deductive coding approach	Breast cancer pathway	Misinterpretation of symptoms<br>Initial symptom management with OTC medicines or waiting to see if it improves<br>Women disclosed to friends and relatives, most advised to go to the hospital, but some advised to wait and monitor or see a traditional healer<br>Misdiagnosis and Complex multiple stage referral process led to delays<br>Initial and later stage management through traditional and faith-based healing	Selection bias (not able to include those who did not attend hospital)<br>Remembering of participants and recall bias	Access, negotiation and acceptance
Hobenu and Naab 2022	Teriary clinic, Accra Region	Qualitative (exploratory)	15 Women with cervical cancer	Purposive sampling, in depth interviews, thematic analysis	Cervical cancer from diagnosis to accessing treatment	Misinterpretation of symptoms led to delays in identifying at services<br>Husbands, relatives and friends advised to use herbal treatments<br>Misdiagnosis and Delays due to waiting a long time to get results, having to go to many hospitals to get diagnosed and rescheduling of appointments	Small sample and purposive sampling leading to selection bias	Access and negotiation
Agyemang 2021	Tertiary clinic, Ashanti Region	Qualitative (ethnographic approach)	31 breast cancer patients (up to stage 3), relatives, nurses and doctors observed and 29 took part in interviews	Purposive (max variation) sampling, 2 time point observations, interviews, thematic analysis	Breast cancer formal diagnosis to treatment	Deliberate miscommunication to stop drop out<br>Lack of honest discussion about fertility concerns meant patients fears were not addressed and consequently led to loss to follow up<br>Lack of support around financial issues<br>Costs were barrier to adherence in HCP opinion	Selection bias (not able to include those who did not attend hospital)<br>Small sample	Acceptance

For peer review only

12

Supplementary Table 3: Evidence map using socio ecological model and candidacy as a framework of access to care

Author and year	Design	Location	Setting	Cancer	Intrapersonal	Interpersonal	Institutional	Community	Policy
Agbeko et al., 2020	Qualitative	Kumasi	Clinic	Breast	A, B	A, C	A, B	A	
Agbokey et al., 2019	Qualitative	Kumasi	Clinic	Breast	A, B, C	A, C	B, C	A	
Agyemang 2021	Qualitative	Kumasi	Clinic	Breast			C		C
Asamoah et al., 2018	Quantitative	Accra	Clinic	Prostate	A				
Asobayire et al., 2015	Qualitative	Upper East	Community	Breast	A	A	B	A	
Asoogo et al., 2015	Qualitative	Kumasi	Clinic	Breast	A	A, B		A	
Atobrah et al., 2012	Qualitative	Accra	Clinic	Multi	A, C	A	B	A, C	
Ayandipo et al., 2020	Mixed methods	Multi-site	Multi-site (health facility)	Multi	B, C	B, C	B, C		C
Aziato et al., 2015	Qualitative	Accra	Clinic	Breast	C	C	B	C	
Binka et al., 2019	Qualitative	Volta	Community	Cervical	A	A, C	A, B, C	A	A, C
Bonsu et al., 2019	Qualitative	Kumasi	Clinic	Breast	A, C	A	A, B, C	A, B, C	

Brinton et al., 2017	Quantitative	Accra / Kumasi	Clinic	Breast	A	A	A	A	
Dadzie et al., 2017	Quantitative	Accra	Clinic	Vulva	C		C		
Dedey et al., 2016	Quantitative	Accra	Clinic	Breast	B		B		
Dunyo et al., 2018	Quantitative	Volta	Clinic	Cervical	A				
Hobenu and Naab 2021	Qualitative	Accra	Clinic	Cervical	A	A	B		
Iddrisu et al., 2021	Qualitative	Accra	Clinic	Breast	C	A	C	C	C
Martei et al., 2018	Qualitative	Accra	Clinic	Breast	A, C	A, C		A, C	C
Mburu 2021	Qualitative	Kumasi	Clinic	Breast	A	A	B	A,C	
Nartey et al., 2018	Quantitative	Accra / Kumasi	Clinic	Cervical	A	A		A	
O'Brien et al., 2011	Quantitative	Kumasi	Community	Multi	A, B, C		B, C	C	C
Obrist et al., 2014	Quantitative	Kumasi	Clinic	Breast	C	C	C	C	C
Salifu et al., 2021	Qualitative	Kumasi	Clinic	Prostate			C	C	
Sanuade et al., 2021	Qualitative	Accra	Clinic	Breast	C	C	B, C	C	
Scherber et al., 2014	Quantitative	Kumasi	Clinic	Breast	A, C		A, C	C	
Twahir et al., 2021	Quantitative	Accra	Clinic	Breast			B		C

For peer review only - http://bmjopen.bmj.com/site/about/guidelines.xhtml

14

Yamoah et al., 2013	Quantitative	Accra	Clinic	Prostate	A, C				
Yarney et al., 2013	Quantitative	Accra	Clinic	Multi	C		C	C	

Key: A= candidacy stage ACCESS, B= candidacy stage NEGOTIATION, C= candidacy stage ACCEPTANCE

For peer review only

BMJ Open

What influences cancer treatment service access in Ghana? A critical interpretive synthesis

Journal:	*BMJ Open*
Manuscript ID	bmjopen-2022-065153.R2
Article Type:	Original research
Date Submitted by the Author:	13-Sep-2022
Complete List of Authors:	Tuck, Chloe; The University of Sheffield, School of Health and Related Research (ScHARR)<br>Akparibo, Robert; The University of Sheffield, School of Health and Related Research (ScHARR)<br>Gray, Laura; The University of Sheffield, School of Health and Related Research (SCHARR)<br>Aryeetey, Richmond; University of Ghana, Population Family and Reproductive Health<br>Cooper, Richard; The University of Sheffield, School of Health and Related Research (ScHARR)
**Primary Subject Heading**:	Global health
Secondary Subject Heading:	Oncology, Health policy, Health services research
Keywords:	Health economics < HEALTH SERVICES ADMINISTRATION & MANAGEMENT, PUBLIC HEALTH, Adult oncology < ONCOLOGY, Health policy < HEALTH SERVICES ADMINISTRATION & MANAGEMENT

SCHOLARONE™
Manuscripts

I, the Submitting Author has the right to grant and does grant on behalf of all authors of the Work (as defined in the below author licence), an exclusive licence and/or a non-exclusive licence for contributions from authors who are: i) UK Crown employees; ii) where BMJ has agreed a CC-BY licence shall apply, and/or iii) in accordance with the terms applicable for US Federal Government officers or employees acting as part of their official duties; on a worldwide, perpetual, irrevocable, royalty-free basis to BMJ Publishing Group Ltd ("BMJ") its licensees and where the relevant Journal is co-owned by BMJ to the co-owners of the Journal, to publish the Work in this journal and any other BMJ products and to exploit all rights, as set out in our licence.

The Submitting Author accepts and understands that any supply made under these terms is made by BMJ to the Submitting Author unless you are acting as an employee on behalf of your employer or a postgraduate student of an affiliated institution which is paying any applicable article publishing charge ("APC") for Open Access articles. Where the Submitting Author wishes to make the Work available on an Open Access basis (and intends to pay the relevant APC), the terms of reuse of such Open Access shall be governed by a Creative Commons licence – details of these licences and which Creative Commons licence will apply to this Work are set out in our licence referred to above.

Other than as permitted in any relevant BMJ Author's Self Archiving Policies, I confirm this Work has not been accepted for publication elsewhere, is not being considered for publication elsewhere and does not duplicate material already published. I confirm all authors consent to publication of this Work and authorise the granting of this licence.

For peer review only

What influences cancer treatment service access in Ghana? A critical interpretive synthesis

Chloe Tuck[1], Robert Akparibo[1], Laura Gray[1], Richmond Aryeetey[2], and Richard Cooper[1]

1. School of Health and Related Research, University of Sheffield, Sheffield, UK
2. School of Public Health, University of Ghana, Legon, Accra, Ghana

Corresponding author:

Chloe Tuck, School of Health and Related Research, University of Sheffield, 30 Regent Street, Sheffield, S1 4DA, UK

Chloeztuck@gmail.com

Cztuck1@sheffield.ac.uk

Phone: +447402110248

Word count: 5147

Keywords: critical interpretive synthesis, Ghana, access to cancer treatment, socio-ecological model, health policy

For peer review only

ABSTRACT

Objectives: Multiple social-cultural and contextual factors influence access to and acceptance of cancer treatment in Ghana. The aim of this research was to assess existing literature on how these factors interplay and could be susceptible to local and national policy changes.

Design: This study uses a critical interpretive synthesis approach to review qualitative and quantitative evidence about access to adult cancer treatment services in Ghana, applying the socio-ecological model and candidacy framework.

Results: Our findings highlighted barriers to accessing cancer services within each level of the socio-ecological model (intrapersonal, interpersonal community, organisational and policy levels), that are dynamic and interacting, e.g, community level factors influenced individual perceptions and how they managed financial barriers. Evidence was lacking in relation to determinants of treatment non-acceptance across all cancers and in the most vulnerable societal groups due to methodological limitations.

Conclusions: Future policy should prioritise multi-level approaches, for example improving the quality and affordability of medical care while also providing collaboration with traditional and complementary care systems to refer patients. Research should seek to overcome methodological limitations to understand the determinants of accessing treatment in the most vulnerable populations.

Strengths and limitations of this study

- A strength of this study is the combination of purposive and systematic searches, and the reflexive approach to developing the search strategy which enabled it to cover a wide range of articles.
- Additionally, the critical interpretive synthesis involved a critique of the literature to identify methodological limitations and research gaps.
- However, as only published academic articles are included in this study, it may overlook other forms of evidence, including locally generated and day-to-day working understandings.
- The interpretation of the evidence will reflect the inherent biases in world view of the lead author.

INTRODUCTION

Cancer is a growing burden in low and middle income countries (LMICs)(1,2). Despite efforts by the World Health Organisation (WHO) to prioritise tackling cancer inequity, hurdles remain due to the limited evidence to inform cost-effective decision-making and the high expense of cancer control(1). In Ghana, cancer treatment is focused in large referral centres in major cities, with disparity in resources and health worker expertise in rural areas, and limited coverage of the National Health Insurance Scheme (NHIS)(3–7). Policy efforts to expand cancer services are further hindered when patients prioritise traditional alternatives over orthodox cancer services(8). Multiple social-cultural, economic and health system factors can influence how patients access, navigate, and choose suitable cancer care in Ghana(3). In addressing this, there is a relative lack of public health surveillance data. There have been recent attempts to reconcile this(9), but a comprehensive understanding remains elusive. One alternative approach is to consider the relevance of theory.

For peer review only - http://bmjopen.bmj.com/site/about/guidelines.xhtml

Socio-economic model and candidacy framework perspectives on cancer treatment access

One important way of understanding the complexity and various factors previous described in relation to cancer treatment service access is to consider this in terms of the socio-ecological model (10). This has been used in many settings to map barriers to healthcare engagement from a systems perspective (11,12). The socio-ecological model considers the individual within an ecosystem of intrapersonal, interpersonal, community, health organisational, and policy influences. This has been applied extensively to map systems factors since it was developed by Bronfenbrenner (10), including health behaviours in several African settings (11,12). The process of an individual accessing cancer services is dynamic and delays in access can occur at multiple stages. Patients may experience barriers presenting at services, negotiating the care pathway, being offered and accepting treatment. A Critical interpretive synthesis (CIS) exploring health access in the UK (13) highlighted that this involves dynamic interactions between the individual, the health environment and health professionals. The term 'access' often overlooks this dynamism, whilst terms such as 'uptake' provide a narrow view that overlooks patient demand and service navigation afterwards.

The candidacy framework provides a multi-stage interacting process of patient access holistically. For example, negotiation of services is overlooked in asylum seekers and refugees(14). Van der Boor and colleagues describe candidacy in two broad stages 'access' (identification of eligibility, navigation of services) and 'negotiation' (permeation of services, appearance, adjudications, offers and resistance, and dynamic interactions with local services)(14). Candidacy has been applied to understand patient-doctor interactions influencing cancer health seeking behaviours(15) and in an African context(16).

This paper builds on the issue of factors relating to cancer treatment service access in LMIC settings such as Ghana by presenting the findings of an evidence review that was informed by theory. The primary aim was to systematically review and critique literature from a systems perspective to understand factors influencing cancer treatment service access in Ghana. A further aim was to assess the strengths and limitations of methods associated with existing research relating to this topic.

METHODS

Using the RETREAT (Review question, Epistemology, Timescale, Resources, Expertise, Audience/purpose, Type of data) framework(17), critical interpretive synthesis (CIS) was considered to be the most suitable approach (13). CIS has been used in a variety of policy and health service settings(18–20) and combines systematic and purposive approaches to identify multiple types of evidence and identify themes following an evidence critique. This involves considering how the problem has been constructed, underlying assumptions and epistemology, and how this has influenced the methodology and conclusions (13).

Search strategy and literature search

The search strategy to find articles on access of adult cancer treatment in Ghana was developed using the question framework PerSPECTiF (Perspective, Setting, Phenomenon of interest, Environment (optional Comparison) Timing, Findings) (21) in consultation with an information specialist. Firstly, primary systematic searches were undertaken. This was tested and refined through pilot searches, before conducting comprehensive searches in Medline (via OVID), Web of Science, CINAHL and African Index Medicus. These databases were chosen following University of Sheffield librarian advice, and after the initial database scoping exercise in Medline (via OVID) and Google Scholar. The database search strategy and terms used can be found in Supplementary Table 1. Initial searches were

For peer review only - http://bmjopen.bmj.com/site/about/guidelines.xhtml

conducted on 26/03/2021 and the databases were searched for updates on 29/03/2022. Search terms were composed of multiple equivalent thesaurus terms and phrases to cover three elements: Ghana, health service access/uptake of services, and cancer. Hand searches were performed using citation follow-up, identified relevant individual journals, and in the reference listed of included papers.

Study selection

The lead author (CT) screened all titles and abstracts using an agreed inclusion criteria whilst two other authors (RA, RC) conducted quality checks of 10% of the sample screened. Any disagreement was discussed and settled among authors. Initial screening highlighted ambiguity in the screening criteria, which was further refined to ensure consistency prior to formal selection. Inclusion criteria included only primary research conducted with a 10-year time frame to align with Ghana's increased policy interest in efficiently expand national health insurance packages (6). Initial screening highlighted the need for a focused exclusion criteria which was again informed by the question framework PerSPECTiF (21). Applying the PerSPECTiF framework , the phenomenon of interest ('access') was defined holistically through the candidacy framework (13). Thus, article screening sought to include articles relating to access throughout the entire patient pathway. The setting included all levels of the socio-ecological model to provide a systems perspective. Potentially qualifying abstracts were read in full, and only the full texts papers that meet the review inclusion criteria were included and reviewed.

Data extraction and synthesis

Data was extracted from included papers to facilitate decision making and an audit trail (See data extraction in Supplementary Material). The lead author (CT) used a standardised data collection form to extract data from the included studies. To eliminate data extraction bias, two reviewers (RA and RC) two other authors (RA, RC) checked 10% of the extraction. There was no discrepancy observed between the lead author extraction and the sample reviewed. Key data extracted were setting, approach, population and sample, methods design, sampling, data analysis, cancer and stage studied, and the corresponding texts cross-tabulated against the socio-ecological model (10) and the candidacy framework. Data was collected as line of arguments (13). First order constructs (taken directly from the data in articles) and second order constructs (author reports from articles) were extracted and separately noted within the framework. It was noted where study authors made further (secondary) inferences and assumptions from data that were not primary findings, but relevant to the themes. Researchers' limitations were recorded. The data were segregated into qualitative, quantitative, and mixed methods studies and each interpreted qualitatively. A synthesising argument(13) was applied to map first and second order findings across studies to interpret the evidence and create new concepts that draw on the whole body of evidence (third order/synthetic constructs). Inferences were mapped across the studies to enable the body of evidence to be critiqued by research question construction, methods, and conclusions and how these fit into the general findings, to identify trends in the literature and limitations with current approaches. To bring together themes in candidacy, the candidacy framework was summarised into three main stages. Van der Boor use two stages(14), however, as themes were identified, it was noted that treatment acceptance and the interactions around this over time play a pertinent role in the patient pathway in Ghana. Thus, the adapted three stage model also notes the importance of the dynamics of treatment acceptance. As part of the synthesis process, the primary literature from the data extraction were revisited and reinterpreted with emerging evidence to ensure critical details or limitations were not missed.

For peer review only - http://bmjopen.bmj.com/site/about/guidelines.xhtml

Critical appraisal

A streamline critical appraisal for major and methodological flaws was conducted using the critical appraisal checklist published in Dixon Woods et al.,(13). The quality of quantitative and qualitative findings was assessed in terms of reliability and trustworthiness (22). In line with the CIS, articles were not favoured based on quality alone but contribution of rich insights. The CIS deals with weak evidence through including a critique of methods and approach. Lead author (CT) appraised all the included papers with 10% of the sample being crossed checked by RC.

Patient and Public Involvement statement

No members of the public or patients were involved in this research.

RESULTS

Search results

Systematic searches in 4 databases and in 6 journals performed in March 2021 (updated in March 2022) identified 312 citation. After duplicate removal, 203 potentially relevant abstracts were screened, subsequently 78 articles were identified for full text screening. A further 16 abstracts were identified for full text screening following citation and reference searching. Twenty-eight articles were selected for inclusion (see PRISMA flow diagram in Figure 1). These comprised 15 qualitative, 12 quantitative studies and 1 mixed methods study.

A summary of the articles reviewed is included in Supplementary Material (Table 2). Applying the candidacy framework and socio-ecological model, key themes were identified and are presented below. The evidence mapping table (Supplementary Material, Table 3) highlight the candidacy stage and level within the socio-ecological model that the articles addressed, as judged by lead author.

Accessibility defined through a 'candidacy' lens

The candidacy framework (13) proved valuable in assessing how healthcare access has been approached, from a holistic perspective. Treatment acceptance was a key emerging issue where there was a gap in understanding. Within treatment acceptance there were multiple aspects – delays accepting, interruptions, choosing alternatives and incompletion or loss to follow up, non-compliance, refusal for referral and non-acceptance of diagnosis. This was a dynamic process.

Although the full candidacy process has been considered in research, there were key gaps in how it had been approached and some aspects warranted further exploration. Seven studies aimed to explore delays with initial contact with cancer services on identification of need (presentation), yet the reasons for eventual non-presentation could not be explored in most studies that were clinic based, as all patients surveyed eventually reached treatment centres. Two studies quantified acceptance as having complete follow up and treatment completion. A further four noted delays and high non-acceptance/loss to follow up but did not formally explore them at an individual psychosocial level as this was not within the research aims. Twelve qualitative studies explored individual barriers to accepting care. However, this was not always the primary focus but emerged in the findings (23,24). As these were sampled from a clinic, they represented patients who eventually (despite delays or interruptions) accessed treatment, so the enablers and barriers in those who ultimately dropped out was unknown. Therefore, in depth qualitative psychosocial information on treatment incompletion was not collected. Although the literature on acceptance was predominantly breast cancer related,

For peer review only

there was some limited evidence it occurred in other cancers, but the extent and reasons for this were not explored.

Barriers and enablers of cancer service access interpreted through the socio-ecological model

The findings on enablers and barriers to candidacy for cancer treatment were mapped using the socio-ecological model to consider the Ghanaian health ecosystem. These are summarised in Figure 2.

Intrapersonal

Financial barriers

Inability to afford treatment was reported as a barrier leading to delays in, and non-acceptance of care. This was also noted by traditional herbalists (25) and health workers (3). Yamoah (26) found that it encompassed socioeconomic factors, travel costs and lost work. Although two studies indicated it was a greater barrier in those from a low income,(27,28) Sanuade (29) suggested this was regardless of socioeconomic status. Four studies(23,28–30) demonstrated that high cost of medical treatment led to use of herbal and traditional alternatives. Prioritisation of finances on family led to delays in accepting treatment (23,24,31). Financial barriers impacted negotiation of care(31); those from a lower income were more likely to experience longer wait times (32).

Fears and beliefs about treatment and its outcomes

Obrist(33) found patients who believed in the efficacy of treatment were more likely to complete treatment in single variable analysis, although this was not significant in multivariate models where potential confounding factors were controlled for. Fears and beliefs pose individual level barriers from qualitative finding. This included fear of treatment, medicines, and the outcomes (8,24,28,29,34). For example for breast cancer, loosing breasts, womanhood, female identity (24,28,29). Fears around institutional trauma suggested lack of trust health facilities(29). Healthcare professions deliberately miscommunicated to avoid patient fear and drop out (35).

Misunderstanding about cancer signs and symptoms

Lack of knowledge about cancer signs and symptoms led to delays in seeking medical treatment (8,24,28,31,36–38). With breast cancer, lumps were not regarded as serious when painless and sometimes considered part of normal tissue (8,24,28,31). This was influenced by beliefs held in the community and within patients' social networks (8,31,36). Lay beliefs were influenced by the terminology used for breast cancer in the local dialect, which led to poor understanding (39).

Social demographics associations

Evidence was conflicting as to whether age, religion and ethnicity impacted stage of diagnosis, wait times and treatment completion, which may associate differently in different cancers given different demographics and natural histories (26,32,33,40–43). As ethnicities often cluster predominantly in different regions, the potential confounding of local health system and environmental should be checked in future studies. Some evidence indicates that lower education is associated with the presenting of larger tumour masses(43,44) and waiting longer for treatment(32), but this was not a consistent influencer, and whether it was associated with treatment acceptance was not explored. One study exploring treatment pathways for young people with chronic diseases suggested community beliefs were more influential than educational status (36). Another found that community beliefs and norms influenced the perceptions of breast cancer regardless of socio-economic status (39). Despite clear financial barriers to treatment; there was no evidence that income status and

For peer review only - http://bmjopen.bmj.com/site/about/guidelines.xhtml

occupation were associated with presentation and acceptance of treatment, but low income status was associated with increased wait times in one study (32).

Interpersonal

Marital relationships influence treatment seeking

Qualitative evidence in female cancer suggests husbands influence their wives' treatment seeking behaviours and acceptance(8,24,27,29,31,38,39,45), by controlling financial decisions about treatment.

Family prioritisation delayed treatment

Women prioritised other activities linked to their economic, family and social roles, such as working for more money, treating children, and paying school fees. These lead to delays presenting for, negotiating and accepting treatment (8,23,24,28,31). Caring for others meant patients put the needs of others first, neglecting their own health. This aligned with findings on the unaffordability of treatment.

Close support networks influence treatment access

Patients understanding about cancer, its causes, and how they engage with care was influenced by close friends and family (8,23,28,34,36–38,45). Misinformation could lead to late presentation, delay help seeking, and use of alternatives (8,23,36,37). For women, lack of husband, family and friend support delayed treatment seeking (27,45). Familial financial support was an enabler for some to seek treatment(23,45) whereas family neglect may impede access (3).

Institutional

Healthcare personnel as gatekeepers to medical and alternative care

Poor detection at primary health facilities, community pharmacy and private settings, may have delayed diagnosis (3,8,23,31,36,44–46). Some women sought over the counter (OTC) medications for pain management (37). Seeking assistance from someone other than a nurse or doctor was associated with a larger mass at diagnosis for breast cancer, which could include a diverse mix of pluralist and community supports (44). Some health professionals also advised herbal alternatives, delaying medical cancer treatment (29). A mixed method study found an inability for facilities to diagnose cancer, improper documentation and filing of patient folders and workload – likely exacerbated by a shortage of healthcare workers trained in oncology outside of major tertiary centres (3). In agreement, qualitative studies with patients found misdiagnosis was common (37,38). There were delays due to: the complex referral process, waiting a long time to get results, having to go to many hospitals and laboratories to be diagnosed, and consultant rescheduling (37,38). Delays between referral and starting radiotherapy were suggested to be due to resource availability, whilst irregular medicine supply also meant patients had to source medicine outside hospitals at high cost (23,47). Patients showed negative perceptions of the care system and professionals (27,29,29,45). Patients perceived treatment delays due to workforce shortage, hospital machines breaking, and medicines shortages (29). These beliefs appear to contribute to a lack of confidence and trust in the health system. Non-completing patients were more likely to harbour negative views such as that the unavailability of cancer medicines delayed their treatment (33). Doubts in the efficacy and disappointment with conventional treatment created barriers to seeking treatment (27) and influenced use of pluralistic treatments (30). Fear of radiation led some not to receive clinically recommended treatment (30).

For peer review only - http://bmjopen.bmj.com/site/about/guidelines.xhtml

Community

The body of literature showed the strong role community beliefs and norms played in shaping access to cancer care. These were interconnected with personal perceptions and health system factors.

Spiritual and traditional beliefs about cancer causes and treatment

In an overwhelming majority of the literature, patients assigned their cancer diagnosis to spiritual causes, which led patients to seek traditional herbal and spiritual treatments, delaying presentation and interrupting medical treatment at multiple stages. This was associated with financial barriers to conventional treatment,(23,28,29) advice from supports such as spouse(45), health workers(29), religious messages(8,29), community networks and beliefs (8,29,31,34,36,39). Additionally alternative therapies were often perceived as more available and acceptable, seen as efficacious (27,28,39,47).

Religiosity plays a diachronous role

Religious beliefs, messages and leaders influenced alternative therapy use(8,29) and caused delays (8,29,37). Yet, religious leaders were identified within patients' trusted support networks(8) and their advice facilitated medical presentation(8,24). Some studies found the church played a supportive role, encouraging women to present at services and providing financial assistance to low-income families (24).

Gender and identity norms

For women with breast cancer, mastectomy was associated with a fear of 'diminished sexuality and femininity' which led many women to delay treatment after seeing an oncologist (24,28,29,31). Unaddressed fears about fertility loss may have increased drop out (35). However, the barriers around identity may differ in other tumours and population groups.

Community networks influenced beliefs and norms

Common misconceptions, beliefs and behaviours held by patients were reinforced by community and social network beliefs (8,29,31,36). Interlinked with community beliefs about cancer is self and socially experienced stigma due to the cause of the disease being spiritual: a curse, mis-endeavour, or the patient being a witch(24,27,39). This led to patients seeking traditional herbal and spiritual treatments, whilst creating shame and secrecy. A retrospective survey found patients who did not complete treatment were more likely to answer they do not know if they were fearful of their community response. However, this likely reflects uncertainty by next of kin respondents, who were substantially higher in 'non-completing treatment' groups (33).

Policy

National Health Insurance Scheme (NHIS) inclusion of cancer care

Based on the financial barriers to treatment reported by patients and healthcare workers, lack of cancer care inclusion within the NHIS was inferred to lead to treatment refusal and delays (3). This was the case for cancers not covered by the scheme(26,42,48), as well as breast(23,24) and cervical cancer (27,40). Breast cancer patients were unanimously frustrated that the NHIS did not cover substantial amounts of treatment and discussed the huge financial burden, especially of chemotherapy drugs (23,24). This meant some women could not start treatment on time (34). This was aggravated by medicines stock-outs(3,23,29,47), requiring purchase elsewhere at additional cost (23,47). Not being insured was significantly associated with a shorter wait time for breast cancer treatment(32), which could reflect preferential treatment to those paying upfront due to delays in the

For peer review only - http://bmjopen.bmj.com/site/about/guidelines.xhtml

administrative process of reimburse NHIS funding. Knowledge of hormone receptor status predicted complete treatment follow up, as this service is offered at a cost (41). Healthcare professionals acknowledged costs were barriers for patients but struggled to broach such topics (35).

Integration of pluralistic care approaches

Given the prominent roles of traditional, herbal and spiritual care, improved integration with the orthodox health system could improve patient access (25,28).

Thirty eight percent of clinical workers surveyed in Ghana attribute treatment disruptions to traditional medicines use (3). Reported use of traditional healers was a significant predictor of late presentation after other variables were controlled for (44). An assessment of factors associated with treatment completion found visiting a traditional healer was a significant predictor of not completing treatment (33). Although alternative support could be concurrent to orthodox medical intervention, over 50% of complementary and alternative medicine practitioners surveyed indicated they did not let patients seek other care alongside(25) and 63% of customers said they declined orthodox therapy while using such therapies (30). Nevertheless, one study found that although 12.2% seek alternative therapies, this only partially explains high rates(73.1%) of loss to follow up (41). Whilst traditional herbalists are considered health professionals, with some services integrated into the Ghanaian health system, poor knowledge of cancer causes and symptoms, and treatment and reluctance to refer to other services are barriers to providing their patients with timely appropriate care (25). This is influenced by a perceived reluctance collaborate from other health system components (25). Mburu suggests the interaction between treatment approaches is non-linear as acts at multiple pathway stages (37).

Critique of the evidence

As part of the CIS approach, a critique of the literature was conducted to identify themes in methods, assumptions, theories, and analysis to identify methodological limitations and research gaps for further studies. A summary of the studies characteristics is displayed in Figure 3.

This review accurately represents evidence in Ghana, which has a high breast cancer contribution. Most studies focus either on presentation delays or treatment interruptions, barriers and treatment delays. Although many studies note treatment incompletion and loss of follow up, only two assess this directly and they note challenges in data collection,(33,41) there are no qualitative studies exploring definitive incompletion. Eighty eight percent (25/28) of studies were based in tertiary treatment clinics in the Greater Accra and Ashanti region, so may not reflect those in other regions. As 89% of qualitative studies sampled purposively from tertiary clinics, this led to biased sampling. They omit those who did not attend, those who dropped out without contact or cannot reach treatment centres. Sampling patients in clinics represents those who eventually presented for and accepted treatment. Barriers could be experienced differently in the under-represented population. As some studies had a small sample and were predominantly Christian, there was limited ethnographic diversity, which may influence generalisability. Findings on the social demographic traits linked to barriers accessing treatment were inconclusive.

Hypothetical inquiry was found to be common across studies. This may lead to error if an individual is not able to accurately predict their behaviour in an unknown situation(49), which may be the case as cancer is stigmatised and not talked about openly (50). Further work should explore and seek to

For peer review only - http://bmjopen.bmj.com/site/about/guidelines.xhtml

understand the impact cognitive bias may have when using hypothetical situations. The gaps in research identified are summarised in box 1 below.

Box 1: Themes and research gaps identified through critiquing the evidence

- Most studies are situated at tertiary clinics, so may not represent rural regions
- Sampling from tertiary clinics means populations who do not present, negotiate the referral pathway from local services and eventually choose treatment are not represented. Understanding what influences access in these missing populations warrants further study
- Treatment drop-out is frequently observed but the reasons for this are not sufficiently explored.
- As most studies focus on breast cancer, there is need to understand the extent of treatment drop out across all cancers and which factors influence this

For peer review only

DISCUSSION

This study used CIS to review multiple types of qualitative and quantitative evidence from literature on access to cancer treatment services in Ghana. Applying 'candidacy' enabled the dynamic and continuous process of accessing, negotiating, and accepting treatment to be explored within the Ghanaian social, economic and policy environment. It highlighted determinants of cancer treatment service access in Ghana are interlinked and within each stage of the socio-ecological model. There is a research gap in understanding the determinants of accessing treatment in the most vulnerable populations due to methodological limitations.

Through this approach, we were able to critique the literature, highlighting trends in methodology and gaps in evidence for future study. The CIS enabled detailed context-specific insights as well as identifying limitations in research approaches, data collection and acquisition challenges to inform future research. A reflective and iterative approach to broaden the breadth of evidence, interpretation and assimilation, was taken. This was particularly valuable to for integrity in research in West African, due to epistemic injustice in how knowledge is perceived and interpreted (51). This is the first study to have explored the applicability of the candidacy framework of healthcare access to a Ghanaian setting. Although this has proven valuable in other African settings(16), this model was developed in a UK setting, therefore it was important to acknowledge pre-existing bias in perspective and thus to critically assess this framing and how it might impact the interpretation of results (51). Selecting globalised frameworks (over those locally synthesised) can lead to interpretive marginalisation. Another limitation of the framework was that we found differences in conceptualising and describing patient engagement pathway with cancer services between studies meant that ascertaining candidacy categories for each required researcher interpretation.

Only published academic articles were included in this study, which may overlook other forms of evidence, including locally generated and day-to-day working understandings. However, this was minimised through an iterative approach to include multiple databases, targeting local journals and supplementary searches.

A methodological challenge was the vague and broad nature of terms relating to 'accessing' healthcare. Thus, there was a need to balance breadth of search with pragmatism to formulate a multi-step search strategy. However, this may mean not all relevant literature was uncovered. Additionally, the critical interpretive nature of the review meant the evidence interpretation, conducted by the lead author,

For peer review only - http://bmjopen.bmj.com/site/about/guidelines.xhtml

will reflect their inherent biases in world view (22). This review was focused on access to cancer treatment in Ghana exclusively, so it is uncertain whether the findings translate to other contexts.

The evidence highlighted financial barriers to cancer treatment access, which interacted with cultural factors and societal influences, such as norms around managing household finances, prioritisation and cultural acceptance of alternative medicines. Globally, catastrophic costs (defined as greater than 40% household effective income) due to non-communicable disease health expenditure are prevalent in LMICs, leading to individuals not taking medicines and impoverishment(52). This exacerbates inequities, having the greatest impact on the poor and leads to detrimental coping strategies(53).

Poor patient clinician relationships have been found to lead people to seek traditional medicines alternatives in Ghana (54). Traditional medicines practitioners were seen to offer more patient-centric, holistic care which was more comforting. A lack of trust was noted for orthodox facilities, which may reflect cultural beliefs as well as past healthcare experiences. An evidence synthesis across LMICs found modern medicines viewed to be harmful and ineffective; suspicion and mistrust of biomedicine lengthened delay and led to alternative use, and the impact of this may be exacerbated in the most vulnerable(50). A qualitative exploration of influences on traditional medicines use in Ghana found their 'pull' by accessibility and alignment with cultural beliefs, whereas scepticism of biomedicine may push people from orthodox healthcare(54).

At a community level, spiritual beliefs about the origin of cancer interacted with personal perceptions at the individual level. Notions and beliefs typically held in the community can be ascribed as lay explanatory models of disease. In accordance with Kleinman(55), lay models of disease can differ from biomedical models based on social experiences and impact how individuals interpret and act on their condition. Community factors influenced explanatory models for hypertension in rural northern Ghana and impact treatment access(56). Similarly, in this review, explanatory models created stigma leading to secrecy and selection of traditional medicines over biomedical intervention.

At a policy level, two key themes where reforms could improve cancer treatment uptake stood out: 1) greater inclusion of cancer treatments within the NHIS, 2) enhanced integration of traditional medicines to provide complementary options to medical care for cancer.

Despite the NHIS aim of achieving universal health coverage for all, there has been notable disparity, with the lowest coverage concentrated in the poor (57,58). Multidimensional barriers due to poverty, dissatisfaction and distrust of the health service and staff may prohibit enrolment (59). Furthermore, catastrophic spending due to out of pocket costs remains high (60). The NHIS plan seeks to cover a considerable amount of the local disease burden and since its nascence the inclusion list has been revised in response to transitions in disease burden and advancing treatments (61,62). Despite breast and cervical cancer in theory being covered(63), it is widely noted women still face considerable financial burden, which this study further highlights. The burden in men however remains less clearly mapped. The high expense of cancer medicines poses a challenge to decision-makers, who must weight costs and benefits when deciding on how to invest health budgets. Approaches such as the health technology assessment platform recently established in Ghana could help prioritise high-cost medicines (64,65).

Another policy area identified in this review was coordination between medical services and traditional medicines. Studies in Ghana have shown traditional medicines users are more likely to be

For peer review only - http://bmjopen.bmj.com/site/about/guidelines.xhtml

poor and not insured on the NHIS (66). The NHIS currently provides services through a plural system of public, faith-based, governmental and private facilities, and includes some traditional medicines (57). Although lack of harmonisation of traditional medicines with the healthcare system (67) was reported in this study, there have been multiple reforms to this end since the Traditional and Alternative Medicine Directorate was established under the Ghanaian Ministry of Health in 2001 (68). Still coordination is hindered by a lack of professional respect from other health professionals (67). Co-current use of alternative forms of care has been found in pregnant women in Ghana. It was highlighted the individual psychosocial and emotional support they provide, which this study found can be lacking patient interactions with orthodox medicine, despite being key for candidacy in cancer treatment (15,69).

The African Union has calls to recognise the importance of traditional medicines and a Global Centre for Traditional Medicines has been established in India (70,71). However, there remain challenges in how traditional and complementary medicines are perceived and deemed efficacious. Barry(72) suggests differently constructed modes of evidence are needed for traditional medicines, as scientific evidence through clinical trials offers a reductionist, narrowly defined view of evidence, that overlooks the role of lived-in social experiences, advocating for the 'expanded epistemology of science' (73). Future policies should seek to improve the affordability and quality of publicly provided medical care whilst harmonising with complementary treatments that align with community beliefs.

Future research is needed to address the research gaps identified. First, to understand the extent of treatment non-adherence across call cancers are what individual, social and health system factors impact this. Second, there are methodological limitations in understanding the views of those who do not attend clinics, which may represent the most vulnerable. Researchers should seek approaches to overcome this which are suitable within the local context.

Acknowledgements
Additionally, the authors would like to thank the School of Health and Related Research Library team for their expertise in designing the search protocol, as well as all researcher staff and graduate at the Universities of Sheffield and Ghana who commented or provided feedback on preliminary results.

Funding

This research was funded by Wellcome as part of a PhD studentship [108903/B/15/Z].

Competing interests

The authors declare no conflicts of interest in undertaking this research.

Data sharing statement

Data sharing not applicable as this is a review of literature, and no datasets were generated.

Contributorship statement

CT, RAk, LG and RC conceptualised the study. CT led the in designing the study, conducted literature search to collect data, perform the data analysis and wrote the first draft. RAk and LG contributed to selecting literature for inclusion in the study and advised on data collection and analysis. RC, RAk, RAr

For peer review only - http://bmjopen.bmj.com/site/about/guidelines.xhtml

and LG reviewed the initial and final draft manuscript and made intellectual inputs to improve quality. All authors read and approved the final manuscript.

CT is responsible for the overall content as guarantor and accepts full responsibility for the work and controlled the decision to publish.

The corresponding author (CT) attests that all listed authors meet authorship criteria and that no others meeting the criteria have been omitted.

Ethical approval

This study does not involve human participants and did not require ethical approval.

Figure legends / captions

Figure 1: PRISMA

*Italicised numbers are from search update conducted March 2022 to identify newly published literature. Diagram adapted from From: Page MJ, McKenzie JE, Bossuyt PM, Boutron I, Hoffmann TC, Mulrow CD, et al. The PRISMA 2020 statement: an updated guideline for reporting systematic reviews. BMJ 2021;372:n71. doi: 10.1136/bmj.n71. For more information, visit: http://www.prisma-statement.org/

Figure 2: Key influences on candidacy for cancer treatment mapped against the socio-ecological model(10–12)

Figure 3: Summary of characteristics of included studies

References

1. World Health Organization. WHO report on cancer: setting priorities, investing wisely and providing care for all [Internet]. World Health Organization; 2020 [cited 2020 Mar 28]. Available from: https://apps.who.int/iris/handle/10665/330745

2. Bray F, Ferlay J, Soerjomataram I, Siegel RL, Torre LA, Jemal A. Global cancer statistics 2018: GLOBOCAN estimates of incidence and mortality worldwide for 36 cancers in 185 countries. CA Cancer J Clin. 2018;68(6):394–424.

3. Ayandipo O, Wone I, Kenu E, Fasehun LK, Ayandipo O, Gaye F, et al. Cancer ecosystem assessment in West Africa: health systems gaps to prevent and control cancers in three countries: Ghana, Nigeria and Senegal. Pan Afr Med J. 2020;35(101517926):90.

4. Adanu K. Household treatment cost of breast cancer disease and cost coping strategy of women, presenting at the Korle Bu Teaching Hospital [Dissertation]. [Accra]: University of Ghana; 2019.

5. Bonsu AB, Aziato L, Clegg-Lamptey JNA. Living with Advanced Breast Cancer among Ghanaian Women: Emotional and Psychosocial Experiences [Internet]. International Journal of Palliative Care. 2014 [cited 2019 Dec 2]. Available from: https://www.hindawi.com/journals/ijpc/2014/403473/abs/

6. Wang, Huihui, Nathaniel Otoo, and Lydia Dsane-Selby. 2017. Ghana National Health Insurance Scheme: Improving Financial Sustainability Based on Expenditure Review. World Bank Studies.

For peer review only - http://bmjopen.bmj.com/site/about/guidelines.xhtml

For peer review only

Washington, DC: World Bank. doi:10.1596/978-1-4648-1117-3. License: Creative Commons Attribution CC BY 3.0 IGO7.

7. Blanchet NJ, Fink G, Osei-Akoto I. The Effect of Ghana's National Health Insurance Scheme on Health Care Utilisation. Ghana Med J. 2012 Jun;46(2):76–84.

8. Bonsu AB, Ncama BP. Recognizing and appraising symptoms of breast cancer as a reason for delayed presentation in Ghanaian women: A qualitative study. PloS One. 2019;14(1):e0208773.

9. Yarney J, Ohene Oti NO, Calys-Tagoe BNL, Gyasi RK, Agyeman Duah I, Akoto-Aidoo C, et al. Establishing a Cancer Registry in a Resource-Constrained Region: Process Experience From Ghana. JCO Glob Oncol. 2020 Apr 17;6:JGO.19.00387.

10. Bronfenbrenner U. Ecological systems theory. In: Six theories of child development: Revised formulations and current issues. London, England: Jessica Kingsley Publishers; 1992. p. 187–249.

11. The Socioecological Model as a Framework for Exploring Factors Influencing Childhood Immunization Uptake in Lagos State, Nigeria. 2021 Jan 8 [cited 2021 Apr 20]; Available from: https://www.researchsquare.com

12. Ngwenya N, Nkosi B, Mchunu LS, Ferguson J, Seeley J, Doyle AM. Behavioural and socio-ecological factors that influence access and utilisation of health services by young people living in rural KwaZulu-Natal, South Africa: Implications for intervention. PLoS ONE [Internet]. 2020 Apr 14 [cited 2021 Apr 20];15(4). Available from: https://www.ncbi.nlm.nih.gov/pmc/articles/PMC7156071/

13. Dixon-Woods M, Cavers D, Agarwal S, Annandale E, Arthur A, Harvey J, et al. Conducting a critical interpretive synthesis of the literature on access to healthcare by vulnerable groups. BMC Med Res Methodol. 2006 Jul 26;6(1):35.

14. van der Boor CF, White R. Barriers to Accessing and Negotiating Mental Health Services in Asylum Seeking and Refugee Populations: The Application of the Candidacy Framework. J Immigr Minor Health. 2020 Feb;22(1):156–74.

15. Tookey S, Renzi C, Waller J, von Wagner C, Whitaker KL. Using the candidacy framework to understand how doctor-patient interactions influence perceived eligibility to seek help for cancer alarm symptoms: a qualitative interview study. BMC Health Serv Res. 2018 Dec 4;18:937.

16. Adeagbo O, Herbst C, Blandford A, McKendry R, Estcourt C, Seeley J, et al. Exploring People's Candidacy for Mobile Health–Supported HIV Testing and Care Services in Rural KwaZulu-Natal, South Africa: Qualitative Study. J Med Internet Res. 2019 Nov 18;21(11):e15681.

17. Booth A, Noyes J, Flemming K, Gerhardus A, Wahlster P, van der Wilt GJ, et al. Structured methodology review identified seven (RETREAT) criteria for selecting qualitative evidence synthesis approaches. J Clin Epidemiol. 2018 Jul;99:41–52.

18. Vélez CM, Wilson MG, Lavis JN, Abelson J, Florez ID. A framework for explaining the role of values in health policy decision-making in Latin America: a critical interpretive synthesis. Health Res Policy Syst. 2020 Sep 7;18(1):100.

For peer review only

19. Khalid AF, Lavis JN, El-Jardali F, Vanstone M. Supporting the use of research evidence in decision-making in crisis zones in low- and middle-income countries: a critical interpretive synthesis. Health Res Policy Syst. 2020 Feb 18;18(1):21.

20. Jarvis T, Scott F, El-Jardali F, Alvarez E. Defining and classifying public health systems: a critical interpretive synthesis. Health Res Policy Syst. 2020 Jun 16;18(1):68.

21. Booth A, Noyes J, Flemming K, Moore G, Tunçalp Ö, Shakibazadeh E. Formulating questions to explore complex interventions within qualitative evidence synthesis. BMJ Glob Health. 2019 Jan 1;4(Suppl 1):e001107.

22. Bryman A. Social research methods. 4th ed. Oxford ; New York: Oxford University Press; 2012. 766 p.

23. Agbokey F, Kudzawu E, Dzodzomenyo M, Ae-Ngibise KA, Owusu-Agyei S, Asante KP. Knowledge and Health Seeking Behaviour of Breast Cancer Patients in Ghana. Int J Breast Cancer. 2019;2019:5239840.

24. Martei YM, Vanderpuye V, Jones BA. Fear of Mastectomy Associated with Delayed Breast Cancer Presentation Among Ghanaian Women. The oncologist. 2018;23(12):1446–52.

25. O'Brien KS, Soliman AS, Annan K, Lartey RN, Awuah B, Merajver SD. Traditional herbalists and cancer management in Kumasi, Ghana. J Cancer Educ Off J Am Assoc Cancer Educ. 2012;27(3):573–9.

26. Yamoah K, Beecham K, Hegarty SE, Hyslop T, Showalter T, Yarney J. Early results of prostate cancer radiation therapy: an analysis with emphasis on research strategies to improve treatment delivery and outcomes. BMC Cancer. 2013;13(100967800):23.

27. Binka C, Nyarko SH, Awusabo-Asare K, Doku DT. Barriers to the Uptake of Cervical Cancer Screening and Treatment among Rural Women in Ghana. BioMed Res Int. 2019;2019(101600173):6320938.

28. Asoogo C, Duma SE. Factors contributing to late breast cancer presentation for health care amongst women in Kumasi, Ghana. Curationis. 2015;38(1).

29. Sanuade OA, Ayettey H, Hewlett S, Dedey F, Wu L, Akingbola T, et al. Understanding the causes of breast cancer treatment delays at a teaching hospital in Ghana. J Health Psychol. 2021;26(3):357–66.

30. Yarney J, Donkor A, Opoku SY, Yarney L, Agyeman-Duah I, Abakah AC, et al. Characteristics of users and implications for the use of complementary and alternative medicine in Ghanaian cancer patients undergoing radiotherapy and chemotherapy: a cross- sectional study. BMC Complement Altern Med. 2013;13(101088661):16.

31. Agbeko AE, Arthur J, Bayuo J, Kaburi BB, Kyei I. Seeking healthcare at their 'right' time; the iterative decision process for women with breast cancer. BMC Cancer. 2020 Oct 19;20(1):1011.

32. Dedey F, Wu L, Ayettey H, Sanuade OA, Akingbola TS, Hewlett SA, et al. Factors Associated With Waiting Time for Breast Cancer Treatment in a Teaching Hospital in Ghana. Health Educ Behav Off Publ Soc Public Health Educ. 2016;43(4):420–7.

For peer review only - http://bmjopen.bmj.com/site/about/guidelines.xhtml

33. Obrist M, Osei-Bonsu E, Awuah B, Watanabe-Galloway S, Merajver SD, Schmid K, et al. Factors related to incomplete treatment of breast cancer in Kumasi, Ghana. Breast Edinb Scotl. 2014;23(6):821–8.

34. Iddrisu M, Aziato L, Ohene LA. Socioeconomic impact of breast cancer on young women in Ghana: A qualitative study. Nurs Open. 2021 Jan;8(1):29–38.

35. Agyemang LS, Foster C, McLean C, Fenlon D, Wagland R. The cultural and structural influences that 'hide' information from women diagnosed with breast cancer in Ghana: an ethnography. BMC Womens Health. 2021 Oct 15;21(1):364.

36. Atobrah D. When darkness falls at mid-day: young patients' perceptions and meanings of chronic illness and their implications for medical care. Ghana Med J. 2012;46(2 Suppl):46–53.

37. Mburu W, Boamah Mensah AB, Virnig B, Amuasi J, Awuah B, Porta C, et al. Pathways to Breast Cancer Diagnosis and Treatment Among Women in Ghana: A Qualitative Study. Womens Health Rep. 2021 Jul 1;2:234–44.

38. Hobenu KA, Naab F. Accessing specialist healthcare: experiences of women diagnosed with advanced cervical cancer in Ghana. Br J Healthc Manag. 2022 Feb 2;28(2):1–11.

39. Asobayire A, Barley R. Women's cultural perceptions and attitudes towards breast cancer: Northern Ghana. Health Promot Int. 2015;30(3):647–57.

40. Nartey Y, Hill PC, Amo-Antwi K, Nyarko KM, Yarney J, Cox B. Characteristics of Women Diagnosed with Invasive CervicalCancer in Ghana. Asian Pac J Cancer Prev APJCP. 2018 Feb 26;19(2):357–63.

41. Scherber S, Soliman AS, Awuah B, Osei-Bonsu E, Adjei E, Abantanga F, et al. Characterizing breast cancer treatment pathways in Kumasi, Ghana from onset of symptoms to final outcome: Outlook towards cancer control. Breast Dis. 2014 Apr 2;34(4):139–49.

42. Asamoah F a., Yarney J, Vanderpuye V, Awasthi S, Venkat P s., Dadzie M a., et al. Definitive Radiation Treatment Patterns and Outcomes for Low Intermediate Risk Prostate Cancer - A Cross Continental Comparative Study between Ghana and the United States of America. Int J Radiat Oncol Biol Phys. 2018 Nov 2;102:e97–e97.

43. Dunyo P, Effah K, Udofia EA. Factors associated with late presentation of cervical cancer cases at a district hospital: a retrospective study. BMC Public Health. 2018 Oct 3;18(1):1156.

44. Brinton L, Figueroa J, Adjei E, Ansong D, Biritwum R, Edusei L, et al. Factors contributing to delays in diagnosis of breast cancers in Ghana, West Africa. Breast Cancer Res Treat. 2017;162(1):105–14.

45. Aziato L, Clegg-Lamptey JNA. Breast cancer diagnosis and factors influencing treatment decisions in Ghana. Health Care Women Int. 2015;36(5):543–57.

46. Twahir M, Oyesegun R, Yarney J, Gachii A, Edusa C, Nwogu C, et al. Real-world challenges for patients with breast cancer in sub-Saharan Africa: a retrospective observational study of access to care in Ghana, Kenya and Nigeria. BMJ Open. 2021 Mar;11(3):e041900.

For peer review only

For peer review only - http://bmjopen.bmj.com/site/about/guidelines.xhtml

47. Salifu Y, Almack K, Caswell G. 'My wife is my doctor at home': A qualitative study exploring the challenges of home-based palliative care in a resource-poor setting. Palliat Med. 2021;35(1):97–108.

48. Dadzie MA, Aidoo CA, Vanderpuye V. Vulva cancer in Ghana - Review of a hospital based data. Gynecol Oncol Rep. 2017;20(101652231):108–11.

49. Fowler, F.J. Jr. Improving Survey Questions: Design and Evaluation [Internet]. Thousand Oaks, CA: Sage; 1995 [cited 2021 Sep 30]. Available from: https://us.sagepub.com/en-us/nam/improving-survey-questions/book4994

50. McCutchan G, Weiss B, Quinn-Scoggins H, Dao A, Downs T, Deng Y, et al. Psychosocial influences on help-seeking behaviour for cancer in low-income and lower middle-income countries: a mixed-methods systematic review. BMJ Glob Health. 2021 Feb;6(2).

51. Bhakuni H, Abimbola S. Epistemic injustice in academic global health. Lancet Glob Health. 2021 Oct 1;9(10):e1465–70.

52. Murphy A, Palafox B, Walli-Attaei M, Powell-Jackson T, Rangarajan S, Alhabib KF, et al. The household economic burden of non-communicable diseases in 18 countries. BMJ Glob Health [Internet]. 2020 Feb 11 [cited 2020 Apr 22];5(2). Available from: https://www.ncbi.nlm.nih.gov/pmc/articles/PMC7042605/

53. Murphy A, McGowan C, McKee M, Suhrcke M, Hanson K. Coping with healthcare costs for chronic illness in low-income and middle-income countries: a systematic literature review. BMJ Glob Health. 2019 Aug 1;4(4):e001475.

54. Gyasi RM, Asante F, Yeboah JY, Abass K, Mensah CM, Siaw LP. Pulled in or pushed out? Understanding the complexities of motivation for alternative therapies use in Ghana. Int J Qual Stud Health Well-Being. 2016;11:29667.

55. Kleinman A. Patients and Healers in the Context of Culture : an exploration of the borderland between anthropology, medicine, and psychiatry [Internet]. Univ of California Press; 1980 [cited 2020 May 19]. Available from: https://www.ucpress.edu/book/9780520045118/patients-and-healers-in-the-context-of-culture

56. Nyaaba GN, Masana L, Aikins A de-Graft, Stronks K, Agyemang C. Lay community perceptions and treatment options for hypertension in rural northern Ghana: a qualitative analysis. BMJ Open. 2018 28;8(11):e023451.

57. Dake FAA. Examining equity in health insurance coverage: an analysis of Ghana's National Health Insurance Scheme. Int J Equity Health. 2018 Jun 18;17(1):85.

58. Dixon J, Tenkorang EY, Luginaah I. Ghana's National Health Insurance Scheme: Helping the Poor or Leaving Them Behind? Environ Plan C Gov Policy. 2011 Dec 1;29(6):1102–15.

59. Millicent Kotoh A, Aryeetey GC, der Geest SV. Factors That Influence Enrolment and Retention in Ghana' National Health Insurance Scheme. Int J Health Policy Manag. 2017 Oct 17;7(5):443–54.

For peer review only

For peer review only - http://bmjopen.bmj.com/site/about/guidelines.xhtml

60. Okoroh J, Essoun S, Seddoh A, Harris H, Weissman JS, Dsane-Selby L, et al. Evaluating the impact of the national health insurance scheme of Ghana on out of pocket expenditures: a systematic review. BMC Health Serv Res. 2018 Jun 7;18(1):426.

61. Ghana NHIS. Ghana NHIS Medicines List 2020 Version 1.0 [Internet]. 2020. Available from https://www.nhis.gov.gh/files/medicineslist(2020).pdf

62. Ghana NHIS. NHIS Review Recommendation — Provide guaranteed Primary, Maternal and Child healthcare for all [Internet]. National Health Insurance Scheme. 2016 [cited 2022 Aug 11]. Available from: http://www.nhis.gov.gh/nhisreview.https://www.nhis.gov.gh/News/nhis-review-recommendation-%E2%80%94-provide-guaranteed-primary,-maternal-and-child-healthcare-for-all-4087aspx

63. Ministry of Health. NHI Policy Framework for Ghana (Revised Version August 2004) [Internet]. 2004. Available from: https://www.ghanahealthservice.org/downloads/NHI_policy%20framework.pdf

64. Ghana's Minister of Health launches the National HTA Steering Committee and calls for HTA institutionalisation in the country [Internet]. iDSI. 2019 [cited 2019 Nov 12]. Available from: https://www.idsihealth.org/blog/ghanas-minister-of-health-launches-the-national-hta-steering-committee-and-calls-for-hta-institutionalisation-in-the-country/

65. Hollingworth S, Gad M. Health technology assessment case study: Ghana. F1000Research [Internet]. 2018 Jul 2 [cited 2019 Nov 12];7. Available from: https://f1000research.com/slides/7-979

66. Amegbor PM, Kuuire VZ, Bisung E, Braimah JA. Modern or traditional health care? Understanding the role of insurance in health-seeking behaviours among older Ghanaians. Prim Health Care Res Dev. 2019;1:e71.

67. Appiah B, Amponsah IK, Poudyal A, Mensah MLK. Identifying strengths and weaknesses of the integration of biomedical and herbal medicine units in Ghana using the WHO Health Systems Framework: a qualitative study. BMC Complement Altern Med [Internet]. 2018 Oct 22 [cited 2020 Mar 27];18. Available from: https://www.ncbi.nlm.nih.gov/pmc/articles/PMC6196414/

68. Ghana Ministry of Health. Traditional Medicines Policy. 2005;15.

69. Dako-Gyeke P, Aikins M, Aryeetey R, McCough L, Adongo PB. The influence of socio-cultural interpretations of pregnancy threats on health-seeking behavior among pregnant women in urban Accra, Ghana. BMC Pregnancy Childbirth. 2013 Nov 19;13:211.

70. WHO, Africa CDC in joint push for COVID-19 traditional medicine research in Africa [Internet]. Africa CDC. [cited 2021 Sep 28]. Available from: https://africacdc.org/news-item/who-africa-cdc-in-joint-push-for-covid-19-traditional-medicine-research-in-africa/

71. WHO establishes the Global Centre for Traditional Medicine in India [Internet]. [cited 2022 Apr 1]. Available from: https://www.who.int/news/item/25-03-2022-who-establishes-the-global-centre-for-traditional-medicine-in-india

72. Barry CA. The role of evidence in alternative medicine: Contrasting biomedical and anthropological approaches. Soc Sci Med. 2006 Jun 1;62(11):2646–57.

For peer review only

73. Baer HA. Toward an Integrative Medicine: Merging Alternative Therapies with Biomedicine. Walnut Creek, CA: Altamira Press; 2004. 224 p.

For peer review only

Figure 1: PRISMA

*Italicised numbers are from search update conducted March 2022 to identify newly published literature. Diagram adapted from From: Page MJ, McKenzie JE, Bossuyt PM, Boutron I, Hoffmann TC, Mulrow CD, et al. The PRISMA 2020 statement: an updated guideline for reporting systematic reviews. BMJ 2021;372:n71. doi: 10.1136/bmj.n71. For more information, visit: http://www.prisma-statement.org/

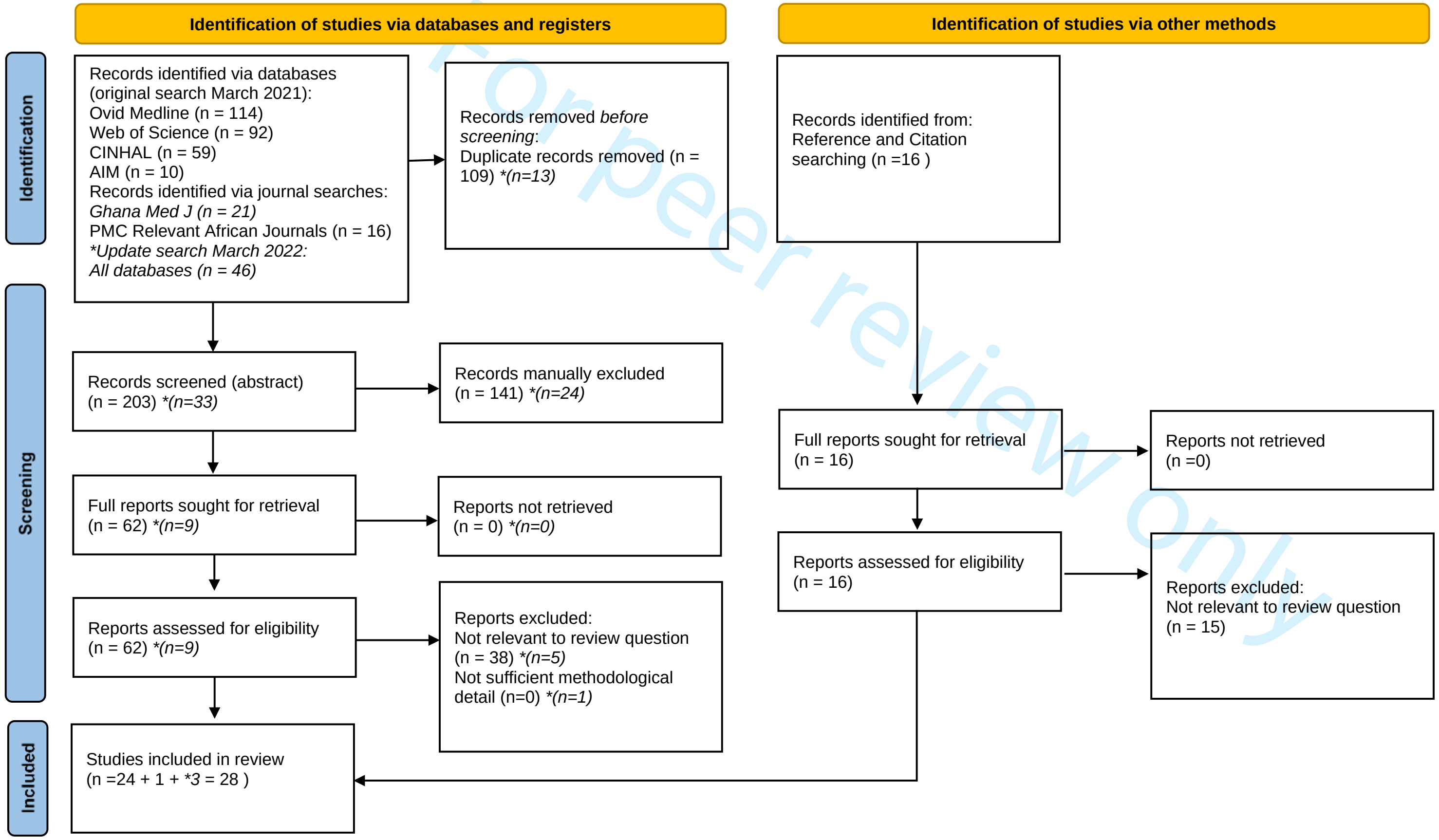

**Italicised numbers are from search update conducted March 2022 to identify newly published literature*

From: Page MJ, McKenzie JE, Bossuyt PM, Boutron I, Hoffmann TC, Mulrow CD, et al. The PRISMA 2020 statement: an updated guideline for reporting systematic reviews. BMJ 2021;372:n71. doi: 10.1136/bmj.n71. For more information, visit: http://www.prisma-statement.org/

For peer review only

Figure 2: Key influences on candidacy for cancer treatment mapped against the socio-ecological model(10–12)

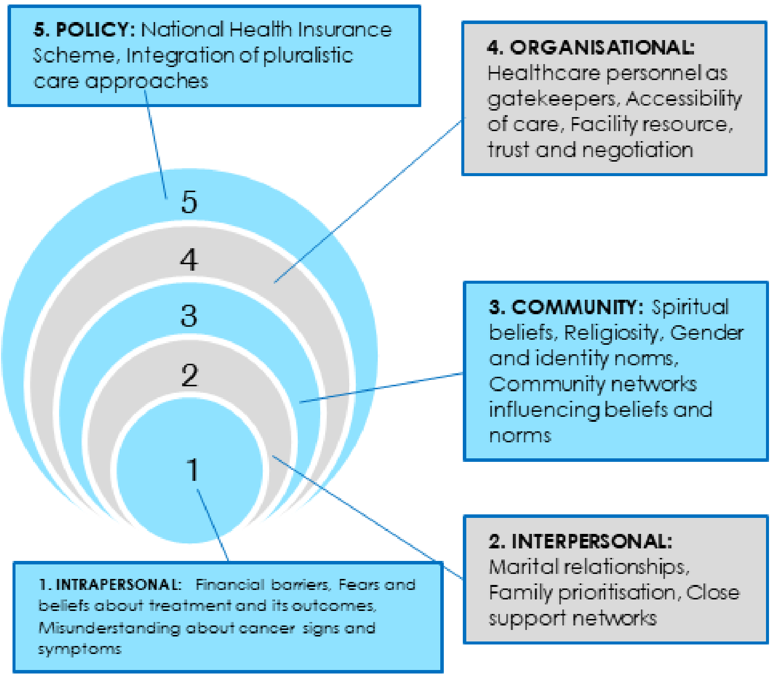

review only

Figure 3: Summary of characteristics of included studies

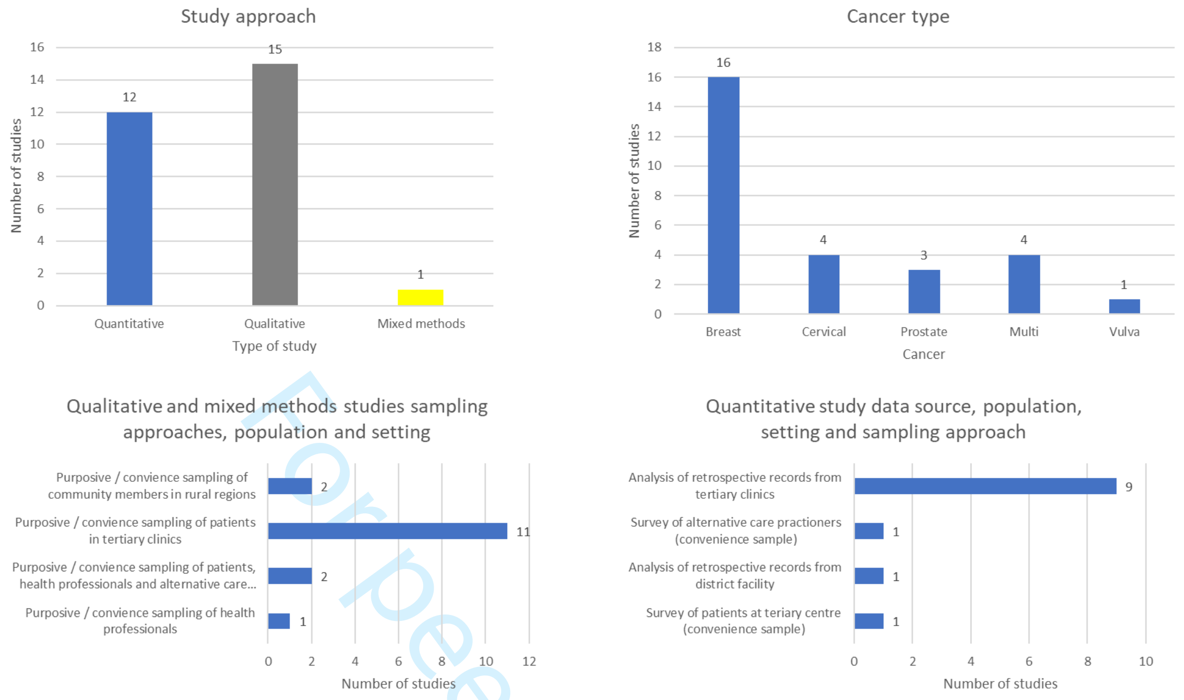

For peer review only

Supplementary Materials

Supplementary Table 1. Systematic search strategy

	**Search topic**		**Ovid MedLine**	**Web of Science**	**CINAHL**	**African Index Medicus**
1	Ghana*		1. exp Ghana/<br>2. Ghana.ti,ab.<br>3. 1 or 2	1. TS=Ghana OR AB=Ghana	1. Ghana	1. Ghana
2	Health service access / patient uptake of services		4. health services accessibility/ or *health services accessibility/ or health equity/ or *healthcare disparities/<br>5.*attitude to health/ or exp health knowledge, attitudes, practice/ or	2. TS=(Health service access)<br>3. AB=((health* NEAR/2 access*) OR (treatment) OR accessibility OR (financ* NEAR/2 (burden* OR impact* OR barrier*)) OR inequalit* OR inequit* OR (social* NEAR/1 economic) OR (social* NEAR/2 (determinant* OR	2. (treatment or therapy or intervention or intervention OR (MH "Health Services Accessibility+"))	-

For peer review only

2

			*treatment adherence and compliance/ or exp patient acceptance of health care/ or exp patient dropouts/ or exp patient satisfaction/ or exp patient preference/ or *treatment refusal/<br>6. ((financ* adj2 (burden or impact or barrier*)) or (health* adj2 access) or inequit* or inequalit* or (social* adj1 economic*) or (social* adj2 (determinant* or disparit* or barrier*)) or accessibility or	disparit* OR barrier*)) OR (catastrophic NEAR/1 cost*))		

For peer review only

3

			treatment* or (catastrophic adj1 cost*)).ti,ab.<br>7. 4 or 5 or 6			
3	Cancer		8. exp Neoplasms/<br>9. cancer*.ti,ab<br>10. 8 or 9	4. TS=cancer OR AB =cancer*	3. Cancer	2. Cancer
Final search			3 and 7 and 10	1 and (2 or 3) and 4 [Indexes=SCI-EXPANDED, SSCI, A&HCI, CPCI-S, CPCI-SSH, BKCI-S, BKCI-SSH, ESCI, CCR-EXPANDED, IC Timespan=All years]	1 and 2 and 3	1 and 2
Total hits (all time)			145	102	68	10

For peer review only

4

Total hits in 10 years (since 01/01/2011)		114	92	62 (59 once duplicated removed)	
Date of search		26/03/2021	26/03/2021	26/03/2021	26/03/2021
Repeated search		29/03/2022	29/03/2022	29/03/2022	29/03/2022

Evidence synthesis tables

Supplementary Table 2: Summary of included studies

Author and year	Setting (region)	Study approach	Population and sample	Methods (design, sampling, data analysis)	Phenomenon	Findings	Limitations noted by authors (if any)	Candidacy stage
Binka et al., 2019	Rural community setting and catholic hospital; North Tongu District (Volta region)	Qualitative (deductive)	Sample 1: 15 cervical cancer patients Sample 2: 40 women aged between 30 and 65 registered at the hospital but not screened	Semi-structured interviews and focus group, convenience sampling, deductive thematic analysis	Cervical cancer screening and treatment uptake	- financial burden of treatment due to a lack of government subsidies for those with low household income<br>- expected provision of financial support with husbands' role<br>- Spiritual beliefs about the cause of disease and in the efficacy of traditional medicine led to spiritual and traditional alternatives being used first.<br>Possible institutional-level barriers (privacy, health worker attitudes, potential misdiagnosis) and unavailability of screening and treatment facilities in rural areas	-	Presentation, negotiation, acceptance
Aziato et al., 2015	Tertiary clinic, Accra Region	Qualitative (exploratory descriptive)	12 Ghanaian women who have undergone a mastectomy (speaking English, Twi, or Ewe)	Semi-structured interviews, purposive sampling, thematic analysis	Breast cancer treatment intentions	Treatment decisions are based on knowledge and perceptions and influenced by husbands and family; without their financial and emotion support may led to delays<br>Patients believed that alternative treatment could treat breast cancer, and sought them after diagnosis, which was influenced by husbands' views<br>Patients perceived health systems reason for treatment delays were:	-	Negotiation, acceptance

5

						- incorrect diagnosis at district level<br>- waiting for biopsy results		
Asoogo et al., 2015	Tertiary clinic, Ashanti Region	Qualitative descriptive	30 women diagnosed with breast cancer who presented with Stage II and Stage III	semi-structured interviews, convenience sampling, content data analysis	Breast cancer presentation	- financial barriers, including leading to alternative use<br>- misinformation, considering abnormalities normal development<br>- fear of treatment and outcomes influenced by supports and others in their community's experiences<br>- prioritising care of other<br>- belief in the efficacy of alternative medicines led to seeking first, influenced by affordability<br>- fear of losing female identity and capacity for motherhood	-	Presentation
Martei et al., 2018	Tertiary clinic, Accra Region	Qualitative (inductive)	31 women with breast cancer	Semi structured interviews, purposive sampling, grounded theory	Breast cancer presentation	- financial barriers including prioritisation costs of family and frustration on poor coverage by NHIS<br>- negative husband views<br>- loss of gender identity<br>- misinformation, considering abnormalities harmless, misconceptions that treatment was deadly<br>The church played a supportive role for presentation.	Bias in sample representation (age, religion Christian)<br>Sampling bias	Presentation, acceptance
Agbokey et al., 2019	Tertiary clinic, Ashanti Region	Qualitative descriptive/exploratory	20 patients / 8 caregivers with breast cancer<br>5 health workers<br>2 herbalists	Semi structured in-depth interviews, purposive sampling, thematic analysis	Breast cancer health seeking behaviours	- financial barriers, including prioritising family costs and frustration at poor coverage by NHIS<br>- family support to secure funds<br>- lack of information<br>- beliefs influenced by husbands and family<br>- husbands role in financial decisions<br>- seeking alternative medicines, influenced by affordability and family and friends	Clinic setting may cause participants reluctance to speak openly	Presentation, negotiation, acceptance
Salifu et al., 2021	Tertiary clinic, urban region	Qualitative at 2 time points	Men with advanced prostate cancer (n = 23), family caregivers (n = 23), healthcare professionals (n = 12).	Semi structured interviews at 2 time points, purposive sampling, thematic analysis, Social constructivist theory and interpretivism	Home based palliative care for prostate cancer	- selection of alternative medicines influenced by challenges in accessing medicines and perceived poor supervision by health professionals	Limited sample location<br>Researcher interpretation bias	Acceptance

For peer review

6

Bonsu et al., 2019	Tertiary clinic, Ashanti Region	Qualitative descriptive/exploratory	11 patients with breast cancer diagnosed at stage II/IV	Semi-structured interviews, purposive sampling, thematic analysis Applying adapted model of health seeking behaviour (Andersen model of total patient delay)	Breast cancer presentation	- poor symptom recognition, considering changes to breasts normal<br>- lack of knowledge on where to seek support<br>-fears and fatalistic perceptions<br>- husbands views<br>- prioritisation of family and work obligations<br>- Misrecognition by health professionals*<br>- Lack of trust in health sector due to perceived mismanagement<br>- spiritual and community held beliefs about cancer led to alternative use (acting at all stages)*<br>- religious leaders supported earlier presentation<br>- some religious messages caused delays<br>- influence of trusted social network (husband, family, religious leaders, health workers in their community, colleagues)	Sample site bias<br>Recall bias	Presentation (predominantly - exceptions*)
Agbeko et al., 2020	Tertiary clinic, Ashanti Region	Qualitative descriptive (deductive)	15 women presenting with stage III/IV breast cancer	Semi-structured interviews, purposively sampling, deductive thematic analysis with priori themes (Andersen Behavioural Model of Health care utilisation)	Breast cancer presentation	- financial barriers to referral process<br>- poor symptom recognition, not regarded as serious, influenced by community beliefs<br>- prioritisation of family roles and finances, influenced by gender identity and roles beliefs<br>- referral delays when first contact not a specialist	Recall bias<br>Unable to follow-up participants for respondent validation<br>Researcher and interviewer bias	Presentation, negotiation, acceptance
Sanuade et al., 2021	Tertiary clinic, Accra Region	Qualitative descriptive (deductive and inductive - exploratory)	20 women who have commenced treatment	4 focus group discussions, purposive sampling following survey, deductive and inductive thematic analysis	Breast cancer treatment delays	- high cost of treatment regardless of social economic status (leading using alternatives)<br>- fear of losing female identity<br>- fear of treatment centre trauma (lack of institution trust)<br>- husbands opinion<br>- impolite treatment by health professionals and perceived corruption<br>- health professionals advising alternative treatments<br>- patients perceived health facility delays including waiting for biopsy results, medicines and workforce shortages, machines breaking<br>- spiritual beliefs led to seeking alternatives, influenced by cost and others (religious leaders, health workers)<br>- community network opinions	Low number of participants	Negotiation and acceptance

For peer review only

7

Atobrah et al., 2012	Tertiary clinic, Accra Region	Qualitative (descriptive)	9 young Ga adults (25-35) with chronic diseases; cancer of the breast, cancer of the ovaries, cancer of the nasopharynx, cancer of the cervix, stroke and chronic renal failure	Multiple narrative interviews and observations (12 each) Purposive sampling (on socio-economic status) Thematic analysis using symbolic interactionism and grief frameworks	Early onset of chronic diseases	- poor symptom recognition given young age<br>- advise to seek alternative therapies from friends/family<br>- misdiagnosis<br>- community beliefs (including of spiritual causes) led to seeking alternative treatments<br>- social networks and community members influenced beliefs and choices	-	Presentation, negotiation, acceptance
Asobayire et al., 2015	(District hospital, unclear) Kassena-Nankana (Navrongo, Upper East Region)	Qualitative (descriptive, inductive)	10 community women (six farmers, two traders and two teachers - aged 25 -56)	Focus group interviews and documentary analysis of current practices, purposive sampling (not stated) for 10 settlements and differing community and socioeconomic status, thematic analysis following an inductive analytical framework	Breast cancer perceptions	- local dialect disease name translation causes misunderstanding<br>- husbands and community leader roles in decision making<br>- community beliefs about the efficacy of traditional medicines and spiritual beliefs lead to alternatives being sought first<br>- potential for positive influence of religious and community leaders and female groups, if engaged	-	Presentation (predominantly)
Iddrisu et al., 2021	Three hospitals in Accra (regional, military and university)	Qualitative (descriptive exploratory)	12 young patients (15-49 year) with breast cancer	Semi structured interviews, purposive and snowball sampling, thematic analysis	Breast cancer socio economic impact	- financial barriers to treatment (including as NHIS did not cover costs)<br>- Negative misbeliefs about treatment, influenced by friends, family and community gossip<br>- spiritual beliefs held in the community led to seeking alternatives, influenced by friends and family	-	Acceptance
Ayandipo et al., 2020	With 3 country analysis: 3 tertiary clinics, four regional, four district facilities (1 private)	Mixed methods cross sectional study	Stakeholders in NCD programme, MoH and CSOs Heads of the selected facilities or units and cancer specialists (120 health workers in Ghana, 10%	Standardised questionnaire, desk based literature review, Thematic analysis Gap analysis using WHO framework for health system strengthening with the 6 pillars	Gaps in cancer control	- patient unaffordability of treatment due to lack of coverage on the NHIS<br>- neglect by families<br>- misdiagnosis, improper documentation and staff shortages, particularly outside of major tertiary centres<br>- shortages of medicines, commodities, and machine breakdown<br>- patient preference for traditional medicines	-	Negotiation and acceptance

For peer review only - http://bmjopen.bmj.com/site/about/guidelines.xhtml

			oncology qualified)					
Nartey et al., 2018	Tertiary clinics in Accra and Ashanti region	Quantitative (retrospective observational)	1725 confirmed invasive cervical cancer cases	Retrospective review of patient record, hospital population sampling, χ2 and logistic (multivariate)regression of factors influencing stage of diagnosis	Cervical cancer stage at diagnosis	- treatment non acceptance for financial reasons<br>- marital status (widowed, divorced, other), ethnicity and (increased) age associated with late diagnosis	Inconsistency, errors and missing data in record keeping<br>Difficulties following up patients/family	Presentation (predominantly)
Scherber et al., 2014	Tertiary clinic in Ashanti region	Quantitative (retrospective observational)	597 breast cancer patients	Review of patient records hospital population sampling, uni and multivariate logistic regression of factors influencing complete treatment follow up	Breast cancer incomplete follow up	- weak positive trend between age and stage diagnosis<br>- no differences in presentation stage whether attended clinic directly or referred from elsewhere<br>- 12.2% patients sought alternative therapies although this does not explain high rates of treatment interruptions/incompletion (73.1% loss to follow up)<br>- presence of hormone status report associated with complete treatment follow up, inferred to reflect the capacity to pay for tests and thus treatment	Inconsistencies with record keeping and missing data Reported barriers only known from those who return for treatment (those who report barriers were more likely to complete treatment)	Acceptance (predominantly, also presentation)
Asamoah et al., 2018	Tertiary clinic in Accra region	Quantitative (retrospective cohort)	1,074 patients with prostate cancer	Retrospective review of records, hospital population sampling, descriptive statistics (χ2 and Fisher's exact tests, Mann-Whitney U tests) and survival analysis	Prostate cancer treatment	- higher median age for presenting with advanced disease	Missing data<br>Hospital records excluded those not able to access	Presentation
Yamoah et al., 2013	Tertiary clinic in Accra region	Quantitative (retrospective cohort)	379 patients with prostate cancer	Retrospective review of patient history, hospital population sampling, descriptive statistics, non-parametric significance tests (due to data skew), survival analysis	Prostate cancer treatment	- treatment non acceptance due to direct and indirect costs being unaffordable, fear of radiation therapy and spiritual beliefs<br>- age was not significantly associated with diagnosis stage	-	Acceptance

For peer review only

Dedey et al., 2016	Tertiary clinic in Accra region	Quantitative (retrospective observational)	205 treated breast cancer patients	Retrospective review of records and patient questionnaire, all patients treated (May - Dec 2013), descriptive statistics, non-parametric tests, Poisson multivariate regression on wait time	Breast cancer waiting time	In a multivariate Poisson regression wait time increased with:<br>- low-income low education, low income, Akan ethnicity, aged 50 or over, marital status (single) but not religion<br>- biopsy time<br>- being insured on NHIS (inferred to be due to delays in recouping funds)<br>- Perceived receipt of adequate information, other perceptions of health workers and service were not significant	Sample representation bias<br>Recall bias	Negotiation
Brinton et al., 2017	Three tertiary clinics in Accra and Ashanti region	Quantitative (retrospective observational)	1,184 patients with malignant breast cancer	Retrospective review of case control study data through structured patient interview, factors influencing tumour mass assessed in multivariate parametric model	Breast cancer diagnosis	In a multivariate model, larger mass at diagnosis correlated with:<br>- low education<br>- marital status (widowed/divorced)<br>- seeking assistance from someone other than a doctor or nurse<br>- use of traditional medicines<br>travel and financial difficulties in seeking care were not significant	Limited sampling catchment may limit possible impact of treatment distance	Presentation
Obrist et al., 2014	Tertiary clinic in Ashanti region	Quantitative (Retrospective case control)	141 women/next of kin with breast cancer	Retrospective review of medical records and semi structured interviews, purposive sampling, statistical analysis by parametric t-tests and non-parametric univariate analysis, multivariate model accounting for confounders using logistic regression	Breast cancer treatment incompletion	- non completing patients were more likely to believe they will not respond, but this was not significant in the LR model<br>- practicing Islam was a significantly higher in non-completing group<br>- age was not significant<br>- explored health systems factors were not significant predictors of completion<br>- seeing a traditional healer after any visit was a significant predictor of not completing after the combined analysis<br>- more non completers believed a traditional healer was better at managing cancer (significant at 10% level)<br>- Understanding what the NHIS cover for breast cancer included was significant predictor of completing treatment	Missing contact information and patient follow up<br>Recall bias by next of kin (more likely in DNC group)	Acceptance
Twahir et al., 2021	Two tertiary clinics in Accra region (as part of a study across	Quantitative (retrospective observational)	299 breast cancer patients undergoing treatment	Retrospective review of patient records in standard collection framework, hospital population sampling, descriptive statistical analysis	Breast cancer access to care	Paying out of pocket costs was associated with receiving more treatment cycles	-	Negotiation and acceptance

For peer review only

10

	Ghana, Kenya and Nigeria)							
O'Brien et al., 2011	Community setting, Ashanti region	Quantitative (descriptive)	42 Traditional Medicine Practice Council (TMPC) registered traditional practitioner	Semi-structured interviews, convenience sampling, descriptive analysis	Traditional herbalists' role in cancer management	Traditional herbalists perceive patient barriers to access include:<br>- cost<br>- lack of knowledge of cancer and fear of treatment<br>- distance and insufficient numbers of health centres<br>- stigma<br>Traditional herbalists poor knowledge of cancer and treatment, and reluctance to refer to other services prevent patients receiving timely care<br>This is influenced by other health professionals' reluctance to collaborate and lack of integration into the health system	-	Presentation, negotiation, acceptance (not specified)
Dadzie et al., 2017	Tertiary clinic in Accra region	Quantitative (retrospective observational)	70 vulva cancer cases	Retrospective review all hospital cases, hospital population sampling, descriptive statistics	Vulva cancer treatment	Cost influenced patient choice (other access barriers were inferred)	-	Acceptance (other access barriers were inferred)
Dunyo et al., 2018	Oncology clinic, Christian hospital (Battor), Volta Region	Quantitative (retrospective observational – cross-sectional analytical)	157 cervical cancer patients	Retrospective review all hospital cases, hospital population sampling, descriptive statistics, non-parametric significance tests, logistic regression of influences on late presentation	Cervical cancer presentation	Education (low) was a significant predictor of late presentation<br>Age was not significant	Missing data, retrospective nature prevented follow up	Presentation
Yarney et al., 2013	Tertiary clinic in Accra region	Quantitative (cross-sectional descriptive)	98 cancer patients	Questionnaire, convenience sampling, descriptive analysis	CAM use in cancer patients	Comparatively high cost of conventional treatment meant only CAM treatment was feasible<br>Lower age, decreased education and being married made it more likely to use CAM in logistic regression model, CAM use influenced by friends and family	Non probabilistic sampling<br>Single institution sample<br>Low response rate<br>Sample bias: excluded those who use CAM but not attending hospital	Acceptance

For peer review only

11

Mburu 2021	Tertiary clinic, Ashanti Region	Qualitative (empircal phenomenological approach)	31 women with breast cancer	Purposive sampling, semi-structured interviews, deductive coding approach	Breast cancer pathway	Misinterpretation of symptoms<br>Initial symptom management with OTC medicines or waiting to see if it improves<br>Women disclosed to friends and relatives, most advised to go to the hospital, but some advised to wait and monitor or see a traditional healer<br>Misdiagnosis and Complex multiple stage referral process led to delays<br>Initial and later stage management through traditional and faith-based healing	Selection bias (not able to include those who did not attend hospital)<br>Remembering of participants and recall bias	Access, negotiation and acceptance
Hobenu and Naab 2022	Teriary clinic, Accra Region	Qualitative (exploratory)	15 Women with cervical cancer	Purposive sampling, in depth interviews, thematic analysis	Cervical cancer from diagnosis to accessing treatment	Misinterpretation of symptoms led to delays in identifying at services<br>Husbands, relatives and friends advised to use herbal treatments<br>Misdiagnosis and Delays due to waiting a long time to get results, having to go to many hospitals to get diagnosed and rescheduling of appointments	Small sample and purposive sampling leading to selection bias	Access and negotiation
Agyemang 2021	Tertiary clinic, Ashanti Region	Qualitative (ethnographic approach)	31 breast cancer patients (up to stage 3), relatives, nurses and doctors observed and 29 took part in interviews	Purposive (max variation) sampling, 2 time point observations, interviews, thematic analysis	Breast cancer formal diagnosis to treatment	Deliberate miscommunication to stop drop out<br>Lack of honest discussion about fertility concerns meant patients fears were not addressed and consequently led to loss to follow up<br>Lack of support around financial issues<br>Costs were barrier to adherence in HCP opinion	Selection bias (not able to include those who did not attend hospital)<br>Small sample	Acceptance

For peer review only

12

Supplementary Table 3: Evidence map using socio ecological model and candidacy as a framework of access to care

Author and year	Design	Location	Setting	Cancer	Intrapersonal	Interpersonal	Institutional	Community	Policy
Agbeko et al., 2020	Qualitative	Kumasi	Clinic	Breast	A, B	A, C	A, B	A	
Agbokey et al., 2019	Qualitative	Kumasi	Clinic	Breast	A, B, C	A, C	B, C	A	
Agyemang 2021	Qualitative	Kumasi	Clinic	Breast			C		C
Asamoah et al., 2018	Quantitative	Accra	Clinic	Prostate	A				
Asobayire et al., 2015	Qualitative	Upper East	Community	Breast	A	A	B	A	
Asoogo et al., 2015	Qualitative	Kumasi	Clinic	Breast	A	A, B		A	
Atobrah et al., 2012	Qualitative	Accra	Clinic	Multi	A, C	A	B	A, C	
Ayandipo et al., 2020	Mixed methods	Multi-site	Multi-site (health facility)	Multi	B, C	B, C	B, C		C
Aziato et al., 2015	Qualitative	Accra	Clinic	Breast	C	C	B	C	
Binka et al., 2019	Qualitative	Volta	Community	Cervical	A	A, C	A, B, C	A	A, C
Bonsu et al., 2019	Qualitative	Kumasi	Clinic	Breast	A, C	A	A, B, C	A, B, C	

Brinton et al., 2017	Quantitative	Accra / Kumasi	Clinic	Breast	A	A	A	A	
Dadzie et al., 2017	Quantitative	Accra	Clinic	Vulva	C		C		
Dedey et al., 2016	Quantitative	Accra	Clinic	Breast	B		B		
Dunyo et al., 2018	Quantitative	Volta	Clinic	Cervical	A				
Hobenu and Naab 2021	Qualitative	Accra	Clinic	Cervical	A	A	B		
Iddrisu et al., 2021	Qualitative	Accra	Clinic	Breast	C	A	C	C	C
Martei et al., 2018	Qualitative	Accra	Clinic	Breast	A, C	A, C		A, C	C
Mburu 2021	Qualitative	Kumasi	Clinic	Breast	A	A	B	A,C	
Nartey et al., 2018	Quantitative	Accra / Kumasi	Clinic	Cervical	A	A		A	
O'Brien et al., 2011	Quantitative	Kumasi	Community	Multi	A, B, C		B, C	C	C
Obrist et al., 2014	Quantitative	Kumasi	Clinic	Breast	C	C	C	C	C
Salifu et al., 2021	Qualitative	Kumasi	Clinic	Prostate			C	C	
Sanuade et al., 2021	Qualitative	Accra	Clinic	Breast	C	C	B, C	C	
Scherber et al., 2014	Quantitative	Kumasi	Clinic	Breast	A, C		A, C	C	
Twahir et al., 2021	Quantitative	Accra	Clinic	Breast			B		C

Yamoah et al., 2013	Quantitative	Accra	Clinic	Prostate	A, C				
Yarney et al., 2013	Quantitative	Accra	Clinic	Multi	C		C	C	

Key: A= candidacy stage ACCESS, B= candidacy stage NEGOTIATION, C= candidacy stage ACCEPTANCE

For peer review only
